# The Impact of Genetic Polymorphisms in Glutamate-Cysteine Ligase, a Key Enzyme of Glutathione Biosynthesis, on Ischemic Stroke Risk and Brain Infarct Size

**DOI:** 10.3390/life12040602

**Published:** 2022-04-18

**Authors:** Alexey Polonikov, Iuliia Bocharova, Iuliia Azarova, Elena Klyosova, Marina Bykanova, Olga Bushueva, Anna Polonikova, Mikhail Churnosov, Maria Solodilova

**Affiliations:** 1 Laboratory of Statistical Genetics and Bioinformatics, Research Institute for Genetic and Molecular Epidemiology, Kursk State Medical University, 18 Yamskaya Street, 305041 Kursk, Russia; 2 Department of Biology, Medical Genetics and Ecology, Kursk State Medical University, 3 Karl Marx Street, 305041 Kursk, Russia; klesovaeu@kursksmu.net (E.K.); bikanovama@kursksmu.net (M.B.); bushuevaoy@kursksmu.net (O.B.); anna-polonikova@rambler.ru (A.P.); solodilovama@kursksmu.net (M.S.); 3 Department of Medical Biological Disciplines, Belgorod State University, 85 Pobedy Street, 308015 Belgorod, Russia; y_u_l_i_a_03@mail.ru (I.B.); churnosov@bsu.edu.ru (M.C.); 4 Division of Neurosurgery, Kursk Regional Clinical Hospital, 45a Sumskaya, 305027 Kursk, Russia; 5 Department of Biological Chemistry, Kursk State Medical University, 3 Karl Marx Street, 305041 Kursk, Russia; azzzzar@yandex.ru; 6 Laboratory of Biochemical Genetics and Metabolomics, Research Institute for Genetic and Molecular Epidemiology, Kursk State Medical University, 18 Yamskaya Street, 305041 Kursk, Russia; 7 Laboratory of Genomic Research, Research Institute for Genetic and Molecular Epidemiology, Kursk State Medical University, 18 Yamskaya Street, 305041 Kursk, Russia

**Keywords:** ischemic stroke, brain infarction, oxidative stress, glutathione, *GCLC*, *GCLM*, single nucleotide polymorphism, gene–gene interactions, gene–environment interactions

## Abstract

The purpose of this pilot study was to explore whether polymorphisms in genes encoding the catalytic (*GCLC*) and modifier (*GCLM*) subunits of glutamate-cysteine ligase, a rate-limiting enzyme in glutathione synthesis, play a role in the development of ischemic stroke (IS) and the extent of brain damage. A total of 1288 unrelated Russians, including 600 IS patients and 688 age- and sex-matched healthy subjects, were enrolled for the study. Nine common single nucleotide polymorphisms (SNPs) of the *GCLC* and *GCLM* genes were genotyped using the MassArray-4 system. SNP rs2301022 of *GCLM* was strongly associated with a decreased risk of ischemic stroke regardless of sex and age (OR = 0.39, 95%CI 0.24–0.62, *p* < 0.0001). Two common haplotypes of *GCLM* possessed protective effects against ischemic stroke risk (*p* < 0.01), but exclusively in nonsmoker patients. Infarct size was increased by polymorphisms rs636933 and rs761142 of *GCLC*. The *mbmdr* method enabled identifying epistatic interactions of *GCLC* and *GCLM* gene polymorphisms with known IS susceptibility genes that, along with environmental risk factors, jointly contribute to the disease risk and brain infarct size. Understanding the impact of genes and environmental factors on glutathione metabolism will allow the development of effective strategies for the treatment of ischemic stroke and disease prevention.

## 1. Introduction

The stroke is the world’s fourth leading cause of long-term disability and death [1,2]. Ischemic stroke (IS) is the most common type of stroke attributed to either a thrombotic or embolic event, causing a decrease in blood flow to the brain. Despite significant advances in acute stroke management over recent decades, stroke-related morbidity and mortality continue to be major public health concerns [3]. In this regard, there is a great deal of interest in identifying new potential targets that may improve the quality of ischemic stroke treatment and prevention.

Ischemic stroke is a multifactorial disease determined by complex interactions between genetic, epigenetic, and environmental factors such as cigarette smoking, air pollution, sedentary lifestyle, and other factors [4,5]. Recent advances in omics techniques have considerably improved our understanding of the molecular mechanisms underlying ischemic stroke, discovered numerous genetic polymorphisms associated with disease susceptibility, and identified novel therapeutic targets [4,6]. Despite notable success in this field of research, the genetic contribution to some molecular mechanisms of cerebrovascular disease remains poorly investigated. A disruption in redox homeostasis, a cellular reduction-oxidation status, and resulted oxidative stress are recognized as major common conditions underlying the pathogenesis of cardiometabolic, cardiovascular, and cerebrovascular disorders [7,8,9]. A growing body of evidence indicates that oxidative stress, attributed to excessive production of reactive oxygen species and/or poor antioxidant defense, contributes to the multistep pathogenetic mechanisms of cerebrovascular disease, starting with atherogenic changes in the carotid arteries such as endothelial dysfunction, macrophage recruitment and adhesion, following atherosclerotic plaque progression and rupture through intraplaque hemorrhage, neovascularization, and fibrous cap thickness—events ultimately leading to artery occlusion and ischemic damage to the brain [9,10].

Glutathione (GSH), a tripeptide consisting of cysteine, glycine, and glutamate, is the main antioxidant and most prevalent thiol-containing peptide regulating the cellular redox homeostasis. GSH exerts numerous physiological functions such as cellular proliferation, mitochondrial maintenance, autophagy, apoptosis, cell cycle regulation, signal transduction, conjugation of xenobiotics, epigenetic regulation, DNA and protein synthesis, protein folding, and glutathionylation of proteins [11,12]. It is important to note that glutathione plays a key role in the protection of cells against oxidative damage attributed to enhanced production of reactive oxygen species (ROS). ROS-associated depletion of GSH contributes to endothelial dysfunction and atherosclerotic plaque rupture, followed by arterial thrombosis [13,14], thereby leading to organ/tissue ischemia and necrosis. Thus, a plethora of physiological functions makes glutathione a critical regulator of a variety of metabolic processes in the cardiovascular and nervous systems, and the dysregulation in GSH biosynthesis may have an etiological role in atherosclerosis, including in carotid arteries. Despite the clear importance of glutathione in vasculature and brain metabolism [15,16], existing data on the roles of genes encoding enzymes involved in glutathione metabolism are surprisingly limited. A few studies have so far investigated the relationship between genes encoding glutathione-metabolizing enzymes and the risk of ischemic stroke and its outcomes. These genetic association studies have been focused on enzymes such as glutathione S-transferases [17,18,19,20,21] and glutathione peroxidases [22,23] that use glutathione as a substrate for the detoxification of foreign compounds and hydrogen peroxide, respectively [19,20,21,23]. No studies have so far been undertaken to investigate the contribution of polymorphic genes involved in glutathione biosynthesis to the development and severity of ischemic stroke, although a deficiency of glutathione in IS patients has been reported by several studies [24,25,26]. The purpose of this pilot study was to evaluate whether genetic variation in glutamate-cysteine ligase, an enzyme that catalyzes the first and rate-limiting step in the production of glutathione, contributes to the risk of ischemic stroke and brain infarct size.

## 2. Materials and Methods

### 2.1. Study Participants and Clinical Examination

Written informed consent was obtained from each patient before enrollment in this study. The study protocol was approved by the Ethical Review Committee of Kursk State Medical University. A total of 1288 unrelated Russians, including 600 patients with a diagnosis of acute ischemic stroke and 688 healthy subjects, were enrolled from Kursk hospitals over two periods, such as between 2007 and 2013 [19,23] and between 2015 and 2017, as described previously [27]. Subjects for the control group were enrolled for the same time periods and included healthy blood volunteers and also hospital-based patients with no history or clinical signs of cerebrovascular, cardiovascular, endocrine, or other chronic diseases. Cerebral infarction was verified by qualified neurologists based on clinical examination, CT, and/or MRI of the brain. The cerebral infarct volume was measured by MRI and expressed in mm (the maximum diameter of the brain damage). We did not include stroke patients in the study with a positive history of cardiac arrhythmias. Baseline, clinical, and laboratory characteristics of the study participants are described in Table 1. The control group was matched to the patient group for sex, age, and body mass index (*p* > 0.05). Study participants completed a validated interviewer-administered questionnaire assessing cigarette smoking, alcohol consumption, physical activity, life stress, dietary intake of fresh fruits and vegetables, and other environmental risk factors, as described previously [28,29]. Information on smoking habits was obtained from all patients with ischemic stroke and 637 healthy subjects. Data on alcohol consumption were available from all IS patients and 251 healthy controls. Information on the intake of fresh fruits and vegetables was obtained from 598 IS patients and 255 healthy controls. As can be seen from Table 1, the number of smokers (ever/never) and alcohol abusers was higher among patients with ischemic stroke than among healthy individuals. No difference in patients’ intake of fresh fruits and vegetables was found between the case and control groups. As can be seen from Table 1, plasma ROS concentrations were significantly higher in patients with ischemic stroke than in healthy subjects (*p* = 0.004), whereas the levels of plasma GSSG were significantly lower in IS patients than in controls (*p* = 0.008). 

### 2.2. SNP Selection

For the study, nine common tagged single nucleotide polymorphisms (SNPs) within both catalytic (rs12524494, rs17883901, rs606548, rs636933, rs648595, and rs761142 of *GCLC*) and modifier (rs2301022, rs3827715, and rs7517826 of *GCLM*) subunits constituting glutamate-cysteine ligase were selected according to the functional properties of the polymorphisms and linkage disequilibrium between them (HapMap data, European population). We aimed at the best coverage of common functional SNPs per a gene: tagSNPs in each haplotype block were selected according to the criteria of r^2^ ≥ 0.8 and MAF (minor allele frequency) > 5%. SNP selection was performed using the Candidate Gene SNP Selection (GenePipe) tool available online at SNPinfo Web Server (https://snpinfo.niehs.nih.gov/snpinfo/selegene.html (accessed on 12 March 2021)).

### 2.3. Genetic Analysis

Approximately 5 mL of venous blood was collected from the cubital vein of each participant into EDTA-coated tubes and maintained at −20 °C until processed. Genomic DNA was extracted from thawed blood samples by the standard procedure of phenol/chloroform extraction and ethanol precipitation. To investigate whether SNPs in glutamate-cysteine ligase genes interact with known susceptibility genes for ischemic stroke, we selected eight SNPs that have previously shown strong associations with disease risk in several genome-wide association studies in European populations, as described previously [27]. The SNPs associated with ischemic stroke risk included rs2417957 of *SLCO1B1*, rs6511720 of *LDLR*, rs4322086 of *RASEF*, rs12449964 of *PEMT*, rs12646447 of *PITX2*, rs899997 (LOC105370913), rs11556924 of *ZC3HC1*, and rs783396 of *AIM1*. The MALDI-TOF mass spectrometry iPLEX platform (Agena Bioscience, Inc., San Diego, CA, USA) was used for genotyping the SNPs. Primer sequences used for genotyping are available upon request. To ensure quality control, 5% of DNA samples were genotyped in duplicates blinded to the case-control status. The concordance rate was >99%. DNA analysis was carried out at the Research Institute for Genetic and Molecular Epidemiology of Kursk State Medical University (Kursk, Russia).

### 2.4. Biochemical Investigations

Fasting venous blood samples were collected from 139 patients with ischemic stroke and 58 healthy subjects in lithium heparin sterile tubes and immediately centrifuged at 1200× *g* using assay kits according to the manufacturer’s instructions (Cell Biolabs, San Diego, CA, USA). Plasma samples were aliquoted and stored at −80 °C until further use. Two alternative biochemical parameters reflecting redox homeostasis such as reactive oxygen species (ROS) and oxidized glutathione (GSSG) levels were measured by Varioscan Flash microplate reader (Thermo Fisher Scientific, Waltham, MA, USA) in all plasma samples of IS patients and healthy subjects (GSSG levels were assessed in 91 patients with IS and 44 controls), as described previously [30]. 

### 2.5. Statistical and Bioinformatics Analysis

Allele and genotype frequencies in the case and control groups were counted and compared by the Fisher exact test to identify significant departures from Hardy–Weinberg equilibrium. Allele, genotype, and haplotype frequencies in the study groups and their associations with disease risk and brain infarct size were analyzed using the SNPStats software (https://www.snpstats.net/start.htm (accessed on 14 April 2021)) [31]. Multiple logistic regression analysis was performed to evaluate the associations of *GCLC* and *GCLM* genotypes with the risk of ischemic events. Continuous variables were analyzed for normal distribution by the Kolmogorov–Smirnov test. Since brain infarct size was not normally distributed, it was presented as a median with an interquartile range (Q1–Q3). Linear regression analysis was used to evaluate the association between SNPs and brain infarct size, which was previously transformed to a normal variable through the procedure of inverse transformation of ranks. All associations were adjusted for age and gender. Replication for associations between SNPs and ischemic stroke was performed using genotype datasets available from the Cardiovascular Cerebrovascular Disease Knowledge Portal (https://cd.hugeamp.org (accessed on 25 March 2022)) and the Gene ATLAS database of UK Biobank (http://geneatlas.roslin.ed.ac.uk (accessed on 26 March 2022)). Linkage disequilibrium (LD) measures such as Lewontin’s *D* and *D*′ [32] were calculated with the LDpair Tool (https://ldlink.nci.nih.gov (accessed on 16 April 2021)) using genotype data from the 1000 Genomes Project and GRCh37 human genome assembly.

The multifactor dimensionality reduction (MDR) method was used to investigate gene–gene and gene–environment (GxE) interactions underlying susceptibility to ischemic stroke and influencing brain infarct size [33]. MDR is a popular data-mining and model-free bioinformatics approach developed to identify high-order interactions between genes and environmental factors contributing to complex traits and multifactorial diseases. The model-based multifactor dimensionality reduction method (*mbmdr*), an extension of the MDR method implemented in a mbmdr package for R [34,35,36], was used to identify a set of statistically significant GxG and GxE interactions instead of a single best model provided by the traditional MDR analysis. The mbmdr analysis algorithm we used has been described in detail previously [28]. Two-, three-, four- and five order GxG and GxE interaction models were evaluated, and their statistical significance *p*-values (P_perm_) were assessed for each *n*-order model through a permutation procedure (1000 permutation tests). Then we counted the number of n-models in which each attribute was involved, and the resultant value was considered as a measure of the contribution of an attribute (a variable such as SNP or risk factor) to the polygenic background, as estimated by the mbmdr method. This methodology was described in detail recently [37]. Post hoc logistic regression was used to assess the associations of pairwise genotype combinations between the lead SNPs involved in the best 2-order mbmdr models of GxG interactions with the risk of ischemic stroke, and the resulting *p*-values were corrected for multiple testing using the false discovery rate (FDR).

Since the investigated SNPs are located in noncoding sequences, we carried out a comprehensive functional annotation of them using various bioinformatics methods. In particular, the SNP Function Prediction tool (FuncPred, https://snpinfo.niehs.nih.gov (accessed on 2 April 2021)) [38] and the Regulome database (https://regulomedb.org (accessed on 3 April 2021)) annotating SNPs with known and predicted regulatory elements in the intergenic regions of the Homo sapiens genome [39] were used to assess the regulatory potential of the polymorphisms. The following bioinformatics recourses were used for assessing the impact of the SNPs on expression levels of *GCLC* and *GCLM* genes in blood, arteries, and brain tissues: the GTEx (Genotype-Tissue Expression) project is a comprehensive public resource for studying tissue-specific gene expression and regulation (https://gtexportal.org (accessed on 4 April 2021)) [40]; eQTLGen is a database on the downstream consequences of trait-related genetic variants (https://www.eqtlgen.org (accessed on 6 April 2021)) [41]. QTLbase curates and compiles genome-wide QTL (Quantitative Trait Loci) summary statistics for many human molecular traits across over 70 tissue/cell types (http://www.mulinlab.org/qtlbase (accessed on 6 April 2021)) [42]. The epigenetic regulation of SNPs through histone marks, open chromatin DNAse, and CTCF binding sites was assessed by the SNPnexus tools utilizing both ENCODE and Roadmap Epigenomics databases (https://www.snp-nexus.org (accessed on 7 April 2021)) [43]. DNA methylation related to the SNPs was assessed through the mQTL analysis provided by QTLbase [42]. Transcription factor binding sites (TFBS) were in silico predicted by Ensembl Variant Effect Predictor [44], Transfac database [45] tools provided by the SNPnexus database, and the atSNP search [46] bioinformatics tools (http://atsnp.biostat.wisc.edu (accessed on 9 July 2021)).

## 3. Results

### 3.1. The Impact of the Studied Polymorphisms on the Risk of Ischemic Stroke and Brain Infarct Size

The genotype and allele frequencies of glutamate-cysteine ligase gene polymorphisms in the study groups are presented in Table 2. Two SNPs, such as rs12524494 of *GCLC* and rs2301022 of *GCLM*, showed a deviation from Hardy–Weinberg equilibrium in both study groups (*p* < 0.01), whereas SNP rs648595 of *GCLC* showed a deviation from HWE only in the IS patients (*p* < 0.001), as assessed by Fisher’s exact test. No departure from HWE was found for other polymorphisms in either the case or control groups (*p* > 0.05). 

The allele frequencies in the study patients were comparable with those reported in other European populations, according to the data published in the 1000 Genomes Project, Phase 3 (http://www.ensembl.org (accessed on 3 April 2021)). As shown in Table 2, SNP rs2301022 of *GCLM* was found to be strongly associated with a decreased risk of ischemic stroke even after adjustment for sex and age (*p* < 0.0001). In particular, genotype rs2301022-T/T showed an association with a decreased risk of ischemic stroke (_cor_OR = 0.36, 95%CI 0.23–0.57, *p* < 0.0001, recessive effect of SNP). In contrast, a heterozygous genotype rs2301022-C/T of *GCLM* was associated with an increased disease risk (_cor_OR = 1.35, 95%CI 1.07–1.69, *p* = 0.01, overdominant effect of SNP). Moreover, an SNP rs648595 of the *GCLC* gene showed an association with ischemic stroke risk but at a borderline statistical level (*p* = 0.049) in a codominant genotypic model. Genotype rs648595-G/T of *GCLC* was associated with an increased risk of ischemic stroke after adjustment for sex and age _(cor_OR = 1.28, 95%CI 1.03–1.60, *p* = 0.029, overdominant effect of SNP). No statistically significant associations of other polymorphisms with disease risk were observed (*p* > 0.05).

Table 3 shows the results of the association analysis between *GCLC* and *GCLM* haplotypes and the risk of ischemic stroke. Nine and six common haplotypes with a frequency greater than 1% were identified in the *GCLC* and GCLM genes, respectively. 

No significant differences in the haplotype distribution for the genes were found between the groups (global haplotype association *p* > 0.05). However, haplotype *H8* of *GCLC* (A-G-G-C-C-G) was associated with an increased risk of ischemic stroke (_cor_OR = 3.37, 95%CI 1.18–9.62, *p* = 0.024). In addition, haplotype *H3* of *GCLM* (C-T-T) possessed a protective effect against the risk of ischemic stroke (_cor_OR = 0.76, 95%CI 0.61–0.95, *p* = 0.016). The haplotype association analysis stratified by risk factors such as tobacco smoking, alcohol consumption, physical activity, life stress, and dietary intake of fresh fruits and vegetables showed that smoking status is a risk factor modifying the relationship between *GCLM* haplotypes and ischemic stroke susceptibility (Table 4). It is found that nonsmoker carriers of two common haplotypes such as A-C-C and C-T-T (each haplotype frequency greater than 20%) of the *GCLM* gene possess a significantly decreased risk of ischemic stroke (*p* < 0.005). Meanwhile, the protective effects of these haplotypes against disease risk were not seen in tobacco smokers. Other environmental risk factors and genetic variants studied did not have any statistically significant synergistic effects on the risk of ischemic stroke. 

The majority of investigated SNPs were in linkage disequilibrium with each other to various degrees, and there were differences in the *D*-values between the Russian population and other populations. Table 5 and Table 6 show the linkage disequilibrium values between SNPs in the *GCLC* and *GCLM* genes, respectively. As can be seen from Table 5, *D*-values between polymorphisms rs636933 and rs12524494 were negative in the studied Russian population and positive in both the European and mixed populations of the 1000 Genomes Project. The principal interpopulation differences in the *D*-values were observed in SNP pairs of the *GCLC* gene such as rs12524494-rs648595, rs636933-rs761142, rs636933-rs606548, rs648595-rs761142, rs648595-rs606548, rs761142-rs17883901, and rs606548-rs17883901 (Table 5). Interpopulation differences regarding D-values were also found between SNPs of the *GCLM* gene. In particular, SNP rs2301022 was in a negative LD with polymorphisms rs7517826 and rs3827715 in the Russian population, whereas LD-values between these SNPs were positive in the European populations (Table 6).

**Figure 1:**
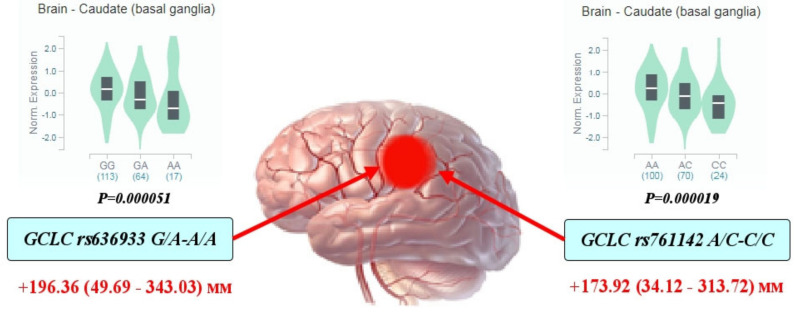
Impact of *GCLC* genotypes on brain infarct size in patients with ischemic stroke: Red arrows indicate the effects of SNPs on the size of brain infarction as assessed by computed tomography (the maximal diameter of brain damage measured by CT and expressed in mm with 95% confidence intervals). The values show an increase in the volume of brain damage in carriers of the assessed genotypes in comparison with the reference genotype. Histograms reflect the influence of genotypes on the normalized level of *GCLC* gene expression in the brain (basal ganglia of the caudal region): data obtained from the GTEx portal (https://www.gtexportal.org (accessed on 2 April 2021)).

**Figure 2:**
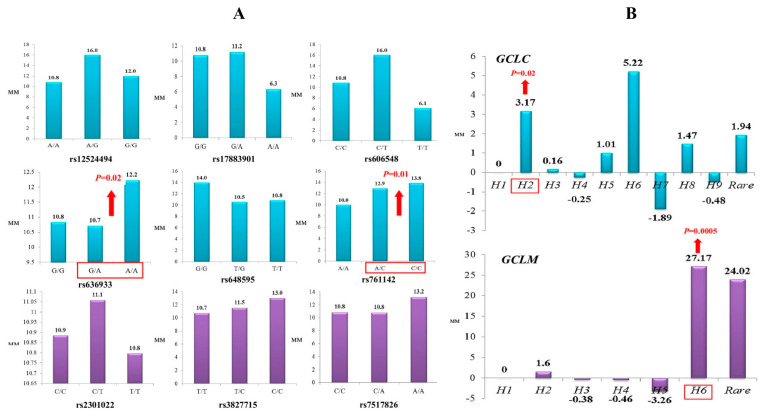
Impact of *GCLC* and *GCLM* genotypes (**A**) and haplotypes (**B**) on brain infarct size in patients with ischemic stroke (linear regression analysis of normalized values of infarct size): (**A**) Histograms represent median values of infarct size (mm) in ischemic stroke patients with various *GCLC* (blue color) and *GCLM* (violet color) genotypes. Significant impact of the polymorphisms on infarct lesion size is indicated by red arrows. (**B**) Histograms represent changes in infarct lesion size (mm) in the carriers of various *GCLC* (blue color) and *GCLM* (violet color) haplotypes relative to the *H*1 haplotype. Significant impact of the haplotypes on brain infarct size is indicated by red arrows.

Through a multiple linear regression with adjustment for sex and age, we analyzed the impact of glutamate-cysteine ligase genotypes and haplotypes on the normalized brain infarct size. As can be seen from Figure 1, the carriage of genotypes rs636933 G/A-A/A of *GCLC*, in comparison with the G/G genotype, was associated with a statistically significant increase in the size of brain infarction by 196.36 mm (*p* = 0.0089, dominant effect of SNP). The rs761142 A/C-C/C genotypes of the *GCLC* gene were also characterized by a significant effect on brain infarct volume compared to the A/A genotype (*p* = 0.015, dominant effect of SNP). Figure 2 summarizes the impact of *GCLC* and *GCLM* genotypes (A) and haplotypes (B) on infarct size in the brains of patients with ischemic stroke. Common haplotype *H2* of *GCLC* (A-A-G-C-C-G) was found to be associated with an increased size of brain infarction (*p* = 0.02). Haplotype *H6* of *GCLM* (A-T-T) showed a strong impact on the size of the damaged brain in patients with ischemic stroke: carriage of this haplotype increased the volume of brain damage by 27 mm more than carriage of the common *H1* haplotype (*p* = 0.0005). Other haplotypes of the *GCLC* and *GCLM* genes had no effect on brain infarct size.

### 3.2. Replication Analysis for SNP-Disease Associations in Independent Populations

A replication study for SNP-ischemic stroke associations in independent population cohorts was carried out using large-scale genotype datasets available at the Cerebrovascular Knowledge Portal and the UK biobank. The following stroke phenotypes of the Cerebrovascular Knowledge Portal were analyzed for the replication analysis: “large artery atherosclerosis” (TOAST classification) and “all ischemic stroke”. Moreover, stroke-related phenotypes such as “transient cerebral ischaemic attacks and related syndromes” and “stroke, not specified as haemorrhage or infarction” were used for the replication analysis in the UK Biobank cohort. Table 7 shows associations between the *GCLC* and *GCLM* polymorphisms and the analyzed ischemic stroke phenotypes. The association between SNP rs2301022 of *GCLM* and the risk of ischemic stroke (large artery atherosclerosis) was replicated (*p* = 0.03) in the Spanish cohort (the VHIR FMT dataset: 515 cases and 316 controls). However, the polymorphism was negatively associated with stroke risk in the Russian population and positively in Hispanics. As we found in the present study, the carriers of allele rs761142-C of the *GCLC* gene had a larger infarct size than those with the alternative allele. Interestingly, the allele rs761142-C was found to be associated with an increased risk of large artery atherosclerosis (*p* = 0.04) in the mixed population cohort (MEGASTROKE GWAS dataset). Interestingly, polymorphisms of *GCLC* and *GCLM* genes that did not show associations with ischemic stroke risk in our population were found to be associated with disease risk in other populations. In particular, SNPs such as rs12524494 (*p* = 0.006) and rs606548 (*p* = 0.036) of the *GCLC* gene were associated with the risk of large artery atherosclerosis in the MEGASTROKE GWAS dataset, including European populations with sample sizes of 190,513 and 189,632 subjects, respectively. In addition, polymorphism rs17883901 of *GCLC* showed a significant association with ischemic stroke (*p* = 0.023) in the Spanish population (VHIR FMT 2018). In the Spanish population, polymorphisms rs3827715 (*p* = 0.02) and rs7517826 (*p* = 0.026) of the *GCLM* gene were found to be associated with an increased risk of ischemic stroke.

### 3.3. Gene–Gene and Gene–Environment Interactions, Ischemic Stroke Risk, and Brain InfarctSize 

Since ischemic stroke is a multifactorial disorder, it is important to unravel the nature of complex gene–gene and gene–environment interactions that determine a polygenic susceptibility to disease. Following this aim, the model-based multifactor dimensionality reduction method (*mbmdr*) was applied to the dataset to identify a set of high-order GxG and GxE interactions underlying disease susceptibility and influencing the severity of brain damage. The mbmdr analysis was performed on a dataset including, besides *GCLC* and *GCLM* SNPs, information on smoking habits, alcohol consumption, and fruit and vegetable intake (environmental risk factors of stroke), as well as genotypes of eight SNPs associated with disease risk as a result of genome-wide association studies in European populations (GWAS loci), as we described previously [27].

**Figure 3:**
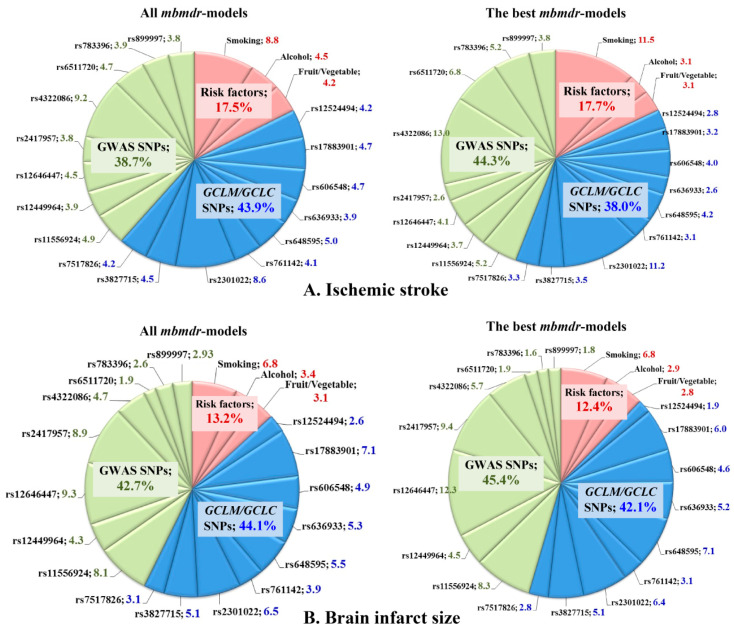
Representation of SNPs and risk factors in the GxG and GxE interaction models associated with susceptibility to ischemic stroke (**A**) and brain infarct size (**B**). Diagrams show the number of *mbmdr*-models (expressed in weighted average percentages with weights 2, 3, 4, and 5 for models of the respective order) in which the investigated SNPs and risk factors are involved. (**A**) (top diagrams): models associated with the risk of ischemic stroke; (**B**) (bottom diagrams): models associated with brain infarct size (in mm). Left diagrams show representation of each SNP and risk factor among all *mbmdr*-models; right diagrams show representation of each SNP and risk factor among the best *mbmdr*-models (i.e., among the 25% of models with the lowest permutation *p*-values). GxG (SNP×SNP) and GxE (SNP×risk factor) interactions were analyzed by the model-based multifactor dimensionality reduction (*mbmdr*) method [35,36].

Summary statistics for all *mbmdr*-models and the best *mbmdr*-models (include the 25% of models with the lowest permutation *p*-values) associated with IS susceptibility are shown in Supplementary Tables S1 and S2, respectively. Figure 3A represents diagrams with the number of *mbmdr*-models per SNP/risk factor associated with the risk of ischemic stroke. In total, 75 two-order, 492 three-order, 1999 four-order, and 8541 five-order SNP-SNP and SNP-risk factor interactions models were found to be significantly (*P*_perm_ < 0.05) associated with the risk of ischemic stroke. Notably, polymorphisms of *GCLC* and *GCLM* were presented in 38% of two-order models, 43% of three-order models, 44% of four-order models, and 45% of five-order models obtained by the *mbmdr* analysis. For comparison, SNPs that are known to be strongly (*p* < 5 × 10^−^^8^) associated with ischemic stroke (i.e., GWAS loci) were presented in 35% of two-order models, 37% of three-order models, 39% of four-order models, and 40% of five-order *mbmdr*-models. In contrast, a percentage of these models (42% of two-order, 40% of three-order, 41% of four-order, and 50% of five-order models) exceeded that for glutamate cysteine ligase SNPs (34% of two-order, 34% of three-order, 42% of four-order, and 38% of five-order models) among the best models with the lowest permutation *p*-values. These data show that the GWAS loci are more strongly linked to the risk of ischemic stroke than the *GCLC* and *GCLM* gene polymorphisms. 

Table 8 shows the best *n*-order gene–gene and gene–environment interactions significantly associated with the risk of ischemic stroke (only the top four models per order are shown). Notably, a significant number of the best *mbmdr*-models comprised polymorphisms of *GCLC* and *GCLM* genes. This was especially noticeable among the five-order *mbmdr*-models shown in Table 8. In addition, GxE interactions, including risk factors such as smoking, alcohol abuse, and low vegetable and fruit intake, may point out a trigger role of the environmental factors in the development of ischemic stroke in individuals with unfavorable genotype combinations of the studied SNPs. Interestingly, a large portion of the *mbmdr*-models associated with disease risk represent interactions between GWAS loci and glutamate-cysteine ligase SNPs, suggesting the involvement of both groups of genes in the polygenic mechanisms of ischemic stroke. Another important finding was that the percentage of models comprising *GCLC/GCLM* genetic variants, and GWAS loci increased progressively as model complexity increased (from two-order to five-order interaction models), while the percentage of mbmdr-models containing environmental risk factors decreased dramatically.

A post hoc analysis of associations between pairwise genotype combinations (diplotypes) and the risk of ischemic stroke was performed for polymorphisms comprising the majority of the two-order *mbmdr*-models. These SNPs include: *GCLM* rs2301022, *RASEF* rs4322086, and *PEMT* rs12449964. Table 9 summarizes the identified statistically significant associations of diplotypes with a predisposition to ischemic stroke. After adjusting for multiple testing by the FDR procedure, we identified diplotypes associated with both increased and decreased disease risk. Carrying *RASEF* rs4322086-G/G × *GCLM* rs2301022-C/T (FDR = 0.04), *RASEF* rs4322086-G/A × *GCLM* rs2301022-C/C (FDR = 0.015), and *RASEF* rs4322086-G/A × *GCLM* rs2301022-C/T (FDR = 0.015) diplotypes reduced the risk of ischemic stroke. On the contrary, diplotypes such as *RASEF* rs4322086-G/G × *GCLM* rs2301022-T/T (FDR = 0.04), *RASEF* rs4322086-G/A × *GCLM* rs2301022-T/T (FDR = 0.015), *RASEF* rs4322086-A/A × *GCLM* rs2301022-C/C (FDR = 0.015), *PEMT* rs12449964-C/C × *GCLM* rs2301022-T/T (FDR = 0.015), and *PEMT* rs12449964-C/T × *GCLM* rs2301022-T/T (FDR = 0.0018) showed protective effects against disease risk.

Summary statistics for all mbmdr-models and the best *mbmdr*-models associated with brain infarct size are shown in Supplementary Tables S3 and S4, respectively. Figure 2B represents diagrams with the number of mbmdr-models per SNP/risk factor associated with brain infarct size. In total, 25 two-order, 109 three-order, 524 four-order, and 2143 five-order gene–gene and gene–environment interactions models were found to be significantly (P_perm_ < 0.05) associated with brain infarct size. Thus, the number of models significantly associated with the volume of brain damage is significantly less than the models associated with the risk of ischemic stroke. Apparently, the studied genes play a greater role in the predisposition to ischemic stroke than in determining the volume of brain infarct in stroke patients. Among all the mbmdr-models, polymorphisms of *GCLC* and *GCLM* were presented in 21% of two-order models, 37% of three-order models, 46% of four-order models, and 47% of five-order models obtained by the mbmdr analysis. This means that the percentage of models representing *GCLC* and *GCLM* SNPs increased progressively with the increasing complexity of the models, while the percentage of models comprising GWAS loci decreased progressively (64% for two-order models, 48% for three-order models, 44% for four-order models, and 40% for five-order models). Table 10 shows the best *n*-order gene–gene and gene–environment interactions significantly associated with brain infarct size (only the top four models per order are shown). Polymorphisms of *GCLC* and *GCLM* genes represented a significant number of five-order models. The great majority of the best *n*-order models comprised interactions between the GWAS loci and glutamate-cysteine ligase SNPs. As a whole, the contributions of the GWAS loci and *GCLC/GCLM* SNPs to ischemic damage in the brain were comparable. 

### 3.4. Functional Annotation of GCLC and GCLM Polymorphisms

Since the investigated polymorphisms of *GCLC* and *GCLM* genes are located in noncoding sequences (introns) of the genes, there is a difficulty in deciphering their biological functions in the pathogenesis of ischemic stroke. In recent years, the increasing multiomics databases and resources, providing integrated access to large-scale genomic, transcriptomic, epigenomic, and other omics datasets, have made it possible to clarify the function of noncoding variants in the human genome [47,48]. Here, we conducted a comprehensive functional annotation for SNPs of genes encoding catalytic and modifier subunits of glutamate-cysteine ligase. Table 11 summarizes the results of functional annotations of *GCLM* and *GCLC* gene polymorphisms using various bioinformatics tools and resources. The regulatory potential of SNPs was analyzed in silico using two bioinformatics tools, such as FuncPred and the Regulome database. 

It was predicted that SNP rs17883901 of *GCLC* had a regulatory potential value of 0.249 and a score of 3a (TF binding + any motif + DNase peak) according to FuncPred assessment and functional annotation of the Regulome database, respectively. An SNP rs648595 of *GCLC* possessed a regulatory potential value of 0.187 and a regulome score of 5 (presence of TF binding or DNase hypersensitivity sites). The polymorphism rs2301022 of the *GCLM* gene associated with ischemic stroke had a Regulome score of 4, suggesting the presence of both TF binding and DNase hypersensitivity sites at this locus.

The GTEx portal, eQTLGen *Consortium*, and QTLbase were used to identify SNP-associated eQTLs (expression quantitative trait loci, genomic loci explaining all or a fraction of the variation in expression levels of mRNAs) in arteries or aorta, brain tissues, and blood. The results of the eQTL analysis are summarized in Supplementary Table S5. All SNPs, except rs17883901 of *GCLC,* represent significant *cis*-eQTLs in the blood according to the eQTLGen *Consortium* and QTLbase resources. All SNPs, except rs12524494, rs17883901, and rs606548 of *GCLC*, are involved in *cis*-eQTLs in the brain tissues. According to the GTEx portal, two SNPs in the *GCLM* gene, rs7517826 and rs3827715, were associated with cis-eQTLs in arteries (data from the GTEx portal, v7, https://gtexportal.org (accessed on 1 April 2021)). In particular, SNP rs7517826 of *GCLM* represents two eQTLs in the tibial arteries (*p* = 2.1 × 10^−6^) and the aorta (*p* = 8.6 × 10^−6^). In addition, SNP rs3827715 was associated with a change in the *GCLM* gene expression in the tibial arteries (*p* = 0.000002) and aorta (*p* = 0.0001). 

Furthermore, we used QTLbase to look for QTLs associated with tissue/cell type-specific molecular functions such as DNA methylation (mQTL), histone modification (hQTL), splicing events (sQTL), and other molecular traits that have been incorporated into the current version of QTLbase (http://mulinlab.tmu.edu.cn (accessed on 3 April 2021)). The results of this analysis are summarized in Supplementary Table S6. Thirteen significant methylation quantitative trait loci (mQTL) in brain tissues were found for polymorphisms rs2301022 (one mQTL), rs3827715 (six mQTLs), and rs7517826 (six mQTLs). No significant mQTLs for the studied SNPs were observed in the arteries or aorta. Interestingly, two polymorphisms, such as rs636933 (*p* = 3.7 × 10^−19^) and rs648595 (*p* = 3.0 × 10^−8^), were associated with tissue-specific splicing events of the *GCLC* gene in the brain.

The SNPnexus resource was used to search for histone modification and open chromatin DNase I hypersensitive sites in brain tissues, arteries, and blood from the ENCODE and Roadmap Epigenomics projects to predict which SNPs were enriched in what kinds of histone marks and DNase peaks (Supplementary Table S7). We found that several SNPs represent subjects to epigenetic modifications, influencing gene activity in normal human astrocytes, aorta, blood, and other tissues. In particular, several polymorphisms have been found to be related to histone marks, such as rs2301022 of the *GCLM* gene (H4K20me1, H3K4me2, and H3K4me1), rs12524494 (H3K36me3), and rs761142 (H4K20me1) of the *GCLC* gene in normal human astrocytes. The rs17883901 polymorphism was associated with histone marked regions H3K4me2 and H3K4me3, hypersensitivity of chromatin to DNase1, and poised promoter activity of the gene in normal human astrocytes. Moreover, the SNP of *GCLM* rs3827715 was related to histone mark H4K20me1 and repressed the promoter flanking region in astrocytic glial cells in humans. The *GCLC* polymorphism rs17883901 was found to be epigenetically regulated via histone H3K4me1 modifications and gene promoter activity in the aorta.

Then we evaluated whether the studied SNPs might regulate enhancer activity by affecting the binding ability of various transcription factors (TF). Bioinformatics resources such as VEP, Transfac, and atSNP have revealed that the investigated polymorphisms are located within DNA motifs that are targets for the binding of multiple transcription factors or transcription factor binding sites (TFBS). In particular, Ensembl Variant Effect Predictor identified that SNP rs17883901 of *GCLC* is located within a DNA motif which is a target for binding of transcription factor complexes such as GCM1:CEBPB, TFAP4:MAX, and OXB2:RFX5, whereas SNP rs606548 creates binding sites for transcription factor complexes GCM1::FIGLA and ETV2:RFX5. 

We also found that the substitution from T to C of rs3827715 of *GCLM* would affect the binding affinity of TFs RUNX3 and RUNX2. According to the TRANSFAC database, polymorphism rs17883901 was predicted to be a subject for a number of transcription factors such as AP1, AP4, AREB6, CEBPA, PPARA, PPARG, and SREBP1.

Finally, the atSNP Search bioinformatics tool was used to identify and quantify best DNA sequence matches to the transcription factor position weight matrices with both the reference and the SNP alleles of the *GCLC* and *GCLM* genes, following evaluation of the statistical significance of the match scores with each allele and calculating the *p*-value of the score difference between the best matches with the reference and SNP alleles [46]. Detailed information on TFBS in the regions of SNPs predicted by the atSNP Search tool is shown in Supplementary Table S8. Numerous transcription factor binding sites regions have been predicted around SNPs with the potential to impact on the expression levels of genes. Molecular functions of transcription factors such as activators, repressors, or dual effects on transcription were assessed by Gene Ontology enrichment analysis hosted by the Enrichr online resource [49] and through manual annotation of the UNIPROT database. Attention should be paid to the polymorphisms of the *GCLC* and *GCLM* genes, which were found to influence the risk of ischemic stroke and brain infarct size. For instance, allele T at SNP rs2301022 of *GCLM* protecting against ischemic stroke was associated with creating the binding sites for numerous transcription factors such as PDX1, ZSCAN26, ELF3, ZNF652, PAX4, SMARCC1, JDP2, FOXP3, ZBTB33, PRRX2, MEF2A, OTX2, DMBX1, RHOXF2, JUND, PITX1, JUNB, CUX1, HNF4A, UNCX, PAX5, FOSL2, HOXB5, BARX1, TFAP2A, SRF, VSX2, FOS, and POU6F2. However, as predicted by the atSNP Search tool, an alternative allele rs2301022-C increasing disease risk was discovered to potentially disrupt the binding sires for the TFs listed above. This allele was found to create TFBS for SPIC, TP53, STAT2: STAT1, RUNX2, SPI1, IRF3, SPI1, PAX3, GABPA, IRF1, HDAC2, IRF5, MYB, IRF1, HDAC2, IRF8, HNF4A, TFCP2, SETDB1, and IRF5.

## 4. Discussion

A problem with the genetic regulation of glutathione metabolism still remains outside the mainstream of research on the molecular mechanisms of cerebrovascular diseases. This happens despite numerous obvious facts clearly demonstrating that glutathione is the most important biologically active molecule, critically required for maintaining cellular redox homeostasis and antioxidant protection as well as for the normal functioning of metabolic processes in the brain. Surprisingly, a few studies have been conducted to assess the relationship between polymorphisms in genes encoding glutathione metabolism enzymes and the risk of ischemic stroke and its complications. In particular, in a Chinese population of 189 IS patients and 169 healthy patients, Man et al. [50] did not reveal statistically significant differences between the groups in the frequency of alleles and genotypes of the −129C/T promoter polymorphism (rs17883901) of the *GCLC* gene. Another study in an Italian population of 100 patients showed that the −129C > T SNP of the *GCLC* gene is associated with cardiovascular events, including the development of cerebral stroke [51]. In a study by Baum and colleagues [52], the −588C > T polymorphic variant (rs41303970), also located in the 5’-flanking region of the *GCLM* gene, showed an association with cognitive impairment that occurs against the background of ischemic stroke, although the association of this SNP with the risk of ischemic stroke has not been investigated. In addition, these *GCLC* and *GCLM* gene polymorphisms showed statistically significant associations with an increased risk of myocardial infarction and endothelial dysfunction [53,54]. Much more often, the objects of genetic association studies in ischemic stroke were polymorphic variants of glutathione-S-transferase genes, which showed associations with disease risk [17,19,21]. Furthermore, in a previous study, we looked at the associations between polymorphisms in glutathione peroxidase genes such as GPX1 (rs1050450), GPX3 (rs2070593), and GPX4 (rs713041) and the risk of cerebral stroke in hypertensive patients, and we observed that SNP rs713041 of the GPX4 gene is associated with an increased risk of stroke [23].

The present study was undertaken for the first time to investigate the contribution of polymorphic genes encoding catalytic and modifier subunits of glutamate-cysteine ligase, a key and rate-limiting enzyme for biosynthesis of glutathione, to the development of ischemic stroke and brain infarct volume. It is observed that polymorphism rs2301022 of the *GCLM* gene is strongly associated with a decreased risk of ischemic stroke regardless of sex and age. In addition, SNP rs648595 of the *GCLC* gene was associated with increased stroke risk, but at a borderline statistical level. Two haplotypes, such as A-G-G-C-C-G of *GCLC* and C-T-T of *GCLM,* were associated with an increased and decreased risk of ischemic stroke, respectively. However, haplotype association analysis stratified by tobacco smoking status showed that two common haplotypes such as A-C-C and C-T-T of *GCLM* possess protective effects against the risk of ischemic stroke, but exclusively in nonsmokers. *GCLC* polymorphisms rs636933 and rs761142, as well as two haplotypes of *GCLM,* were associated with an increased volume of brain infarction in patients with ischemic stroke. The relationship between polymorphism rs2301022 of *GCLM* and the risk of large artery atherosclerosis was revealed in the Spanish cohort. Furthermore, SNP rs761142, which was associated with brain infarct size, has been found to be associated with the risk of large artery atherosclerosis in a mixed cohort (MEGASTROKE) GWAS. Some polymorphisms of *GCLC* (rs12524494, rs606548, and rs17883901) and *GCLM* (rs3827715 and rs7517826) genes that did not show associations with risk of ischemic stroke in our population were associated with disease risk at least in one independent population. The analysis of linkage disequilibrium between DNA polymorphisms revealed that interpopulation differences in the association of alleles with ischemic stroke risk appear to be attributed to features of the haplotype structure of genes in the studied populations.

Modeling gene–gene and gene–environment interactions by the *mbmdr* method showed that polymorphisms at *GCLC* and *GCLM* are involved in epistatic interactions with known candidate genes for ischemic stroke, and their effects on disease susceptibility are comparable with those loci identified by large-scale, genome-wide association studies. However, the contribution of *GCLC* and *GCLM* gene polymorphisms to the volume of brain infarction is greater than the studied GWAS loci. Differences in the associations of *GCLC* and *GCLM* gene polymorphisms with ischemic stroke risk and brain infarct size may be explained by the fact that SNPs have different functional effects in the carotid arteries and brain, and these effects can be modulated through epigenetic mechanisms such as methylation and histone modification, as has been identified by the bioinformatics analysis. The strongest gene–gene interactions contributing to the risk of ischemic stroke were found between SNP rs2301022 of the *GCLM* gene and two GWAS loci, such as rs4322086 of *RASEF* and rs12449964 of *PEMT* (exact biological functions of these genes remain unknown), suggesting that the studied genes are part of a complex genetic predisposition to ischemic stroke.

It is also important to note that the synergic effects of the genes on ischemic stroke risk and brain infarct size are triggered by environmental factors such as tobacco smoking, alcohol abuse, hypodynamia, and fruit and vegetable intake that are well-recognized risk factors for cerebrovascular disease. Bioinformatics analysis showed that polymorphisms of the *GCLC* and *GCLM* genes are functionally important regions involved in the regulation of gene expression in the vasculature and brain through epigenetic mechanisms and allele-specific binding with transcription factors. The rs2301022 polymorphism of the *GCLM* gene showed the most significant association with the risk of ischemic stroke. Bioinformatics analysis showed no phenotypic effects of this polymorphism on gene expression, and there are no studies that have investigated the impact of this SNP on the expression levels of *GCLM*. However, we found that the rs2301022 SNP is part of a common haplotype rs2301022T-rs3827715T-rs7517826C of the *GCLM* gene, which was associated with a protective effect against the risk of ischemic stroke. Since the rs3827715-T and rs7517826-C alleles are associated with a higher level of transcriptional activity of the *GCLM* gene in the arteries and in some parts of the brain, this indicates that the rs2301022-T allele, apparently, is also associated with increased expression of the gene, thereby enhancing the synthesis of glutathione. It is noteworthy that a strong protective effect of the rs2301022-T/T *GCLM* genotype on the risk of ischemic stroke was seen in the suppression of the negative effects of polymorphic genes such as rs4322086 of *RASEF* and rs12449964 of *PEMT* that have been found to be associated with an increased risk of ischemic stroke [55,56]. In addition, bioinformatics analysis allowed us to show that SNPs rs636933 and rs761142 of *GCLC* and rs2301022 of *GCLM* are significant eQTLs with *loss-of-function* effects of alternative alleles on gene expression in the brain, suggesting that an increase in brain infarct size might be the result of a decrease in the expression and/or activity of the genes that lead to a reduced synthesis of glutathione. A study in the British population [57] confirmed our finding that the rs2301022 SNP of the *GCLM* gene is associated with the volume of brain damage following stroke. Thus, despite interpopulation differences in the associations between the gene polymorphisms and stroke phenotypes identified by replication analysis, the present study clearly shows that genetic variation in both catalytic and modifier subunits of glutamate-cysteine ligase determines ischemic stroke susceptibility and brain infarct size, highlighting the importance of *GCLC* and *GCLM* gene polymorphisms for both the molecular pathogenesis of ischemic stroke and disease severity. Thus, functional annotation of SNPs showed that SNPs rs636933 and rs761142 of the *GCLC* gene and rs2301022 of the *GCLM* gene are characterized by statistically significant loss-of-function effects of minor alleles on the expression of the corresponding genes in brain and vasculature tissues. The increase in the area of ischemic damage appears to be the result of a low glutathione level caused by the decrease in the expression and/or activity of the *GCLC* and *GCLM* genes.

It is known that interactions between genetic and environmental factors are jointly involved in the development of ischemic stroke. The findings obtained by our study support this concept. The *mbmdr* method discovered that well-known environmental risk factors for ischemic stroke, such as chronic stress [58], physical inactivity [59], cigarette smoking [60], alcohol abuse [61], and insufficient consumption of fresh vegetables and fruits [62], showed the strongest contribution to disease development in combination with polymorphisms of the *GCLC* and *GCLM* genes, as well as some GWAS loci. The vast majority of GxE models comprise combinations of one or more of these environmental factors. An in-depth analysis of the literature has led us to the assumption that endogenous glutathione deficiency may be a shared condition that occurs as a result of the influence of these risk factors. This assumption is based on the fact that chronic stress, alcohol abuse, and smoking might be responsible for the depletion of glutathione [63,64,65,66,67,68], whereas moderate physical activity and high levels of consumption of fresh vegetables and fruits contribute to an increase in endogenous biosynthesis of glutathione [69,70,71]. In particular, it is known that short-term emotional stress can cause positive changes in the activity of enzymes involved in the synthesis of glutathione [72]. However, chronic stress is associated with the depletion of endogenous glutathione, as has been clearly demonstrated by experimental studies, including in brain tissues [73,74]. Regular consumption of fresh vegetables and fruits, which contain up to 70% of dietary glutathione and its precursors, including a balanced amount of amino acids, vitamins, minerals, and phytochemicals, contributes to a significant increase in endogenous glutathione levels [71,75]. Interestingly, increasing the intake of fresh fruits and vegetables (e.g., green leafy vegetables and citrus fruits, including juice) by one serving per day is associated with a 6% reduction in the risk of ischemic stroke [62]. 

Glutamate-cysteine ligase is a heterodimeric enzyme consisting of a heavy catalytic subunit and a light modifier subunit that are expressed by different genes [76], and interactions between the subunits determine the catalytic efficiency of this enzyme. Activity of glutamate-cysteine ligase and upregulation of *GCLC* gene expression may be induced in response to oxidative stress and GSH depletion through the activation of regulatory elements in the gene promoter [77]. It is known that biosynthesis of GSH in brain cells depends on their ability to uptake extracellular amino acid precursors of glutathione (glutamate, cysteine, and glycine) and/or synthesize sufficient substrates to produce their own GSH. Notably, uptake and metabolism of GSH precursors differ by brain cell type. Unlike neurons, astrocytes can use a variety of amino acids and peptides, which are taken up by excitatory amino acid carrier 1 and converted into substrate amino acids for glutamate-cysteine ligase and glutathione synthetase [78].

The present and previous studies [79,80,81] clearly show that polymorphisms of key genes involved in glutathione biosynthesis such as *GCLC*, *GCLM*, *GSS* (glutathione synthase), and *GGCT* (gamma-glutamylcyclotransferase) are important contributors to the pathogenesis of ischemic stroke. Together with the environmental factors discussed above, these genes determine the level of glutathione metabolism and thus can modulate intracellular GSH content. The study results point out that loss-of-function variants in genes for glutathione metabolizing enzymes along with environmental factors increase the risk of development and severity of cerebrovascular disease through the development of glutathione deficiency. What is the pathophysiological significance of impaired glutathione synthesis for the development and progression of cerebrovascular disease? Glutathione deficiency in the vasculature and brain tissues may contribute to the multistep pathogenesis of cerebrovascular disease. Glutathione deficiency may have a pathogenetic role in all three stages of cerebrovascular disease, such as (1) atherosclerosis of cerebral arteries, (2) acute occlusion of the cerebral artery, and (3) ischemic injury of the brain. Glutathione has been shown to be an important intravascular regulator involved in the control of endothelial growth and the protection of endothelial cells from oxidative damage, inflammation, and atherosclerosis [82,83]. 

Atherosclerosis has been associated with decreased expression of GSH-dependent antioxidant enzymes and an associated decline in glutathione [82,84,85]. A prospective study performed on mice showed that reduced levels of GSH in the aorta are detected both before the onset of atherosclerosis and during its development and progression [86]. It has also been found that the expression level of the *GCLM* gene is decreased in carotid arteries affected by atherosclerosis compared to intact internal mammary arteries [87]. In addition, hypomethylation of CpG sites at the *GCLM* gene in blood leukocytes was found to be associated with acute cerebral stroke [88]. Carotid intima-media thickness, an indicator of carotid and generalized atherosclerosis [89,90], is negatively associated with blood GSH levels [91], providing additional evidence for the role of glutathione deficiency in atherosclerosis of cerebral arteries. Cho and co-workers observed that the increase in GSH by oxidized low density lipoproteins (OxLDL) may afford cellular protection against OxLDL-induced oxidative stress, impairing endothelial cells as an initial step in atherogenesis [92]. Moreover, it has been established that glutathione deficiency reduces the bioavailability of nitric oxide, resulting in the development of endothelial dysfunction, which is a key pathophysiological change in atherosclerosis [93]. Deficiency of reduced glutathione has been revealed in cerebrovascular disease [24] and in the acute phase of ischemic stroke [94], thereby contributing to more severe brain damage, as has been demonstrated by numerous studies [95,96,97,98,99]. It has been demonstrated that the severity of ischemic stroke correlates directly with the level of glutathione deficiency [100].

The brain has a moderate antioxidant capacity, and this limited ability to neutralize ROS makes brain tissues more susceptible to oxidative stress than other organs and tissues, contributing to the development of pathological processes including ischemic stroke [9,101]. Cerebral vessels are the main targets of oxidative stress, which actually plays a decisive role in the formation of ischemic damage to brain tissues and determines the severity of stroke [102]. High levels of glutathione are essential not only for the protection of the central nervous system against oxidative stress but also for normal brain functioning [78]. First, glutathione may represent a physiologic reservoir of glutamate, the most abundant excitatory neurotransmitter in cortical synapses, which is known to participate in many physiologic and pathologic processes, including stroke [103]. Second, glutathione has been proven to play a crucial role in brain ischemia resistance by reducing the loss of nerve function and apoptosis of neuronal cells [104,105]. In humans, an age-dependent decline in GSH has also been found to increase the extent of neuronal injury following ischemic stroke [106,107]. It has been established that astrocytes with glutathione deficiency lose their neuroprotective function, resulting in a decrease in neuron viability and death [108]. Elevation of GSH after acute stroke is considered to be a part of acute adaptive neuroprotective mechanisms during acute stroke [109]. A decrease in reduced glutathione was found to cause protein nitration, S-nitrosylation, as well as the formation of DNA strand breaks in neuronal cells [12]. Moreover, glutathione depletion is an important factor for decreased neuronal viability, apoptosis initiation, and execution [110,111,112]. Howarth and co-workers demonstrated that production of the vasodilator prostaglandin E2 in astrocytes is critically dependent on brain levels of glutathione, and decreased levels of glutathione will lead to dysfunctional cerebral blood flow regulation, resulting in subsequent neuronal damage [113]. Glutathione was found to reduce inflammation and neuronal cell death following brain injury [111]. Reduced glutathione is known to mediate antioxidant responses in the brain, providing a critical defense system for the protection of cells from oxidative stress as well as serving a central role in repairing brain damage after ischemic stroke [16,106]. Finally, it has been experimentally observed that glutathione has the potential to stimulate neurorepair and functional recovery after ischemic stroke [114].

Finally, glutathione deficiency may be the cause of impaired protein folding, leading to endoplasmic reticulum stress and the induction of neuronal cell death following ischemic stroke. The formation of disulfide bonds in maturing proteins is required for their correct tertiary structure, and this process takes place in the endoplasmic reticulum (ER) and is heavily influenced by glutathione, one of the key players in ensuring proteostasis in all types of cells [115,116]. Oxidized and reduced forms of glutathione play distinct functions in protein folding: GSSG, being an oxidant, forms disulfide bonds in a protein, whereas GSH, being a reducing agent, cleaves misbridged disulfide bonds [116,117]. Glutathione deficiency appears to reduce ischemic neurons’ ability to ensure the efficiency of the protein folding process in the endoplasmic reticulum, causing it to become overloaded with misfolded proteins. Thus, there is reason to believe that glutathione deficiency in the cell is the key factor responsible for ineffective protein folding and accumulation of unfolded or misfolded proteins in the ER, culminating in activation of the unfolded protein response and cell apoptosis. Importantly, impaired proteostasis is a major cause of neuronal cell death in ischemic stroke. Ischemic injury of the brain initiates ER stress or unfolded protein response, an adaptive process with the activation of prosurvival mechanisms for the recovery of brain damage after cerebral ischemia [118,119]. However, chronic ER stress plays a detrimental role in nerve cells via triggering diverse proapoptotic pathways [118,119]. 

There are some limitations in the present study that should be addressed. The present study examined a limited number of polymorphisms in the *GCLC* and *GCLM* genes. Therefore, further studies with a larger number of SNPs are required to assess the comprehensive contribution of the genes to the risk of ischemic stroke and postischemic damage to the brain. Furthermore, the observed associations need to be confirmed in independent populations. Low statistical power did not allow detection of reliable estimates of the effects of polymorphisms on glutathione levels because the sample size of IS patients with biochemical investigations of redox homeostasis was too small. A relatively low sample size of the studied population did not allow us to identify gene–environment interactions involving risk factors such as chronic stress, physical inactivity, alcohol abuse, and decreased intake of fresh vegetables and fruits, all of which play a role in the etiology of ischemic stroke.

## 5. Conclusions

Our study was the first to show that genetic variation in subunits of glutamate-cysteine ligase, a key and rate-limiting enzyme for glutathione biosynthesis, contributes to the risk of ischemic stroke and brain infarct size. We found that polymorphisms of the *GCLC* and *GCLM* genes are in tight epistatic interactions with known genes responsible for the risk of ischemic stroke and determining the volume of brain infarction. The multifactorial nature of ischemic stroke is supported by our findings that the synergic effects of the studied genes on disease susceptibility and brain infarct size are triggered by well-recognized environmental risk factors for cerebrovascular disease. Understanding the role of glutathione metabolism disorders caused by the interaction of environmental and genetic factors opens the door to developing new methods for the treatment and prevention of ischemic stroke and its consequences. In particular, N-acetylcysteine and glycine supplementation for the replenishment of endogenous glutathione deficiency is widely regarded as a promising approach to the primary prevention of atherosclerosis and neuroprotective therapy for ischemic stroke and its severity [82,101,120,121]. Finally, the present study supports the urgent need for clinical trials focusing on intravenous administration of reduced glutathione as an adjuvant therapy in the acute phase of ischemic stroke with the goal of protecting neurons from oxidative damage and alleviation of disease outcomes.

## Figures and Tables

**Table 1:** Baseline, clinical, and laboratory characteristics of the study participants.

Baseline and Clinical Characteristics			Controls (*n* = 688)	IS Patients (*n* = 600)	*p*-Value
Age, M ± S.D.			60.8 ± 7.5	61.1 ± 9.8	0.59
Sex, *n* (%)	Males		366 (53.2)	330 (55.0)	0.52
	Females		322 (46.8)	270 (45.0)	
BMI (kg/m^2^), M ± S.D.			24.6 ± 3.8	25.2 ± 4.2	0.11
Brain infarct size (mm in maximal diameter), Me (Q1–Q3)			-	10.8 (5.0–23.9)	-
Hypertension			-	586 (97.7)	-
Coronary artery disease			-	49 (8.2)	-
Diabetes mellitus			-	52 (8.7)	-
Smoking status *		Ever	221 (32.8)	265 (44.2)	**<0.0001**
		Never	452 (67.2)	335 (55.8)	
Alcohol intake *		Abuse	25 (10.0)	116 (19.3)	**0.001**
		Low/moderate	226 (90.0)	484 (80.7)	
Fruits/vegetables intake *		Low	100 (39.2)	283 (47.3)	0.29
		High/moderate	155 (60.8)	315 (52.7)	
Oxidized glutathione (GSSG) in plasma (μmol/L), Me (Q1–Q3) *			1.93 (0.84–5.75)	1.31 (0.46–3.52)	**0.008**
Reactive oxygen species (ROS) in plasma (μmol/L), Me (Q1–Q3) *			2.47 (1.98–3.69)	3.41 (2.43–4.21)	**0.004**

M, mean; S.D., standard deviation; *n*, number; BMI, body mass index; Me, median; Q1–Q3 quartiles; * The number of patients examined with these parameters is described in the Methods Section.

**Table 2:** Association analysis of *GCLC* and *GCLM* gene polymorphisms with ischemic stroke risk.

Gene (SNP ID)	Genotype, Allele	*n* (%)		*p*-Value	_cor_OR (95% CI) *
		Controls (*n* = 688)	IS Patients (*n* = 600)		
*GCLC* A > G (rs12524494)	A/A	543 (93.6)	550 (93.4)	0.98	1.00
	A/G	33 (5.7)	35 (5.9)		1.05 (0.64–1.72)
	G/G	4 (0.7)	4 (0.7)		1.02 (0.25–4.10)
	G	0.035	0.037	0.88	1.03 (0.67–1.59)
*GCLC* G > A (rs17883901)	G/G	585 (86.2)	493 (84.4)	0.30	1.00
	G/A	90 (13.2)	83 (14.2)		1.10 (0.80–1.52)
	A/A	4 (0.6)	8 (1.4)		2.38 (0.71–7.95)
	A	0.072	0.085	0.24	1.19 (0.89–1.59)
*GCLC* C > T (rs606548)	C/C	625 (94)	496 (93.2)	0.88	1.00
	C/T	38 (5.7)	34 (6.4)		1.12 (0.69–1.81)
	T/T	2 (0.3)	2 (0.4)		1.26 (0.18–8.97)
	T	0.032	0.036	0.58	1.14 (0.73–1.77)
*GCLC* G > A (rs636933)	G/G	421 (62.7)	370 (64.5)	0.79	1.00
	G/A	216 (32.2)	178 (31)		0.94 (0.74–1.20)
	A/A	34 (5.1)	26 (4.5)		0.87 (0.51–1.48)
	A	0.212	0.200	0.49	0.93 (0.77–1.13)
*GCLC* G > T (rs648595)	G/G	124 (18.2)	82 (14.1)	**0.049**	1.00
	G/T	335 (49.2)	323 (55.4)		1.20 (0.93–1.54)
	T/T	222 (32.6)	178 (30.5)		0.91 (0.72–1.15)
	T	0.572	0.582	0.60	1.04 (0.89–1.22)
*GCLC* A > C (rs761142)	A/A	388 (57.2)	331 (57.3)	0.92	1.00
	A/C	255 (37.6)	220 (38.1)		1.01 (0.80–1.27)
	C/C	35 (5.2)	27 (4.7)		0.90 (0.54–1.53)
	C	0.240	0.237	0.88	0.99 (0.82–1.18)
*GCLM* C > T (rs2301022)	C/C	344 (51)	286 (51)	**<0.0001**	1.00
	C/T	251 (37.2)	249 (44.4)		1.19 (0.94–1.51)
	T/T	80 (11.8)	26 (4.6)		**0.39 (0.24–0.62)**
	T	0.304	0.268	**0.048**	0.84 (0.70–1.00)
*GCLM* T > C (rs3827715)	T/T	348 (52.5)	293 (52.2)	0.31	1.00
	T/C	258 (38.9)	232 (41.4)		1.07 (0.85–1.36)
	C/C	57 (8.6)	36 (6.4)		0.76 (0.48–1.18)
	C	0.281	0.271	0.60	0.95 (0.80–1.14)
*GCLM* C > A (rs7517826)	C/C	254 (38.7)	207 (37.2)	0.62	1.00
	C/A	306 (46.6)	275 (49.4)		1.10 (0.86–1.41)
	A/A	96 (14.6)	75 (13.5)		0.96 (0.67–1.37)
	A	0.380	0.382	0.92	1.01 (0.86–1.19)

* Odds ratio adjusted for age and sex by multiple logistic regression analysis (codominant model).

**Table 3:** Associations of *GCLC* and *GCLM* haplotypes with the risk of ischemic stroke.

Haplotypes	SNPs						Frequency		*p*-Value	_adj_OR (95%CI) ^2^
	rs12524494	rs636933	rs648595	rs761142	rs606548	rs17883901	Healthy Controls	IS Patients		
***GCLC* Haplotype Frequencies (*n* = 1288)**										
*H1*	A	G	T	A	C	G	0.5383	0.5388	-	1.00
*H2*	A	A	G	C	C	G	0.1536	0.1583	0.87	1.02 (0.80–1.29)
*H3*	A	G	G	A	C	G	0.1575	0.1432	0.45	0.91 (0.72–1.16)
*H4*	G	G	G	C	T	G	0.0325	0.0369	0.63	1.11 (0.72–1.72)
*H5*	A	G	T	A	C	A	0.0278	0.0394	0.17	1.42 (0.86–2.35)
*H6*	A	A	G	C	C	A	0.0333	0.0222	0.25	0.72 (0.42–1.25)
*H7*	A	A	G	A	C	G	0.0253	0.0162	0.28	0.73 (0.42–1.29)
***H8***	**A**	**G**	**G**	**C**	**C**	**G**	0.0081	0.0152	**0.024**	**3.37 (1.18–9.62)**
*H9*	A	G	G	A	C	A	0.0060	0.0180	0.19	1.75 (0.76–4.03)
Rare ^1^	*	*	*	*	*	*	0.0176	0.0118	0.16	0.56 (0.25–1.27)
Global haplotype association *p*-value: 0.11										
***GCLM* haplotype frequencies estimation (*n* = 1285)**										
**Haplotypes**	**rs7517826**		**rs3827715**		**rs2301022**		**Healthy Controls**	**IS** **Patients**	***p*-Value**	**_adj_OR (95%CI) ^2^**
*H1*	C		T		C		0.3536	0.3982	-	1.00
*H2*	A		C		C		0.2660	0.2497	0.097	0.83 (0.67–1.03)
***H3***	**C**		**T**		**T**		0.2692	0.2294	**0.016**	**0.76 (0.61–0.95)**
*H4*	A		T		C		0.0757	0.0835	0.86	0.97 (0.68–1.37)
*H5*	A		C		T		0.0194	0.0249	0.72	1.14 (0.55–2.36)
*H6*	A		T		T		0.0153	0.0123	0.48	0.71 (0.28–1.84)
Rare ^1^	*		*		*		0.0008	0.0020	0.52	2.29 (0.18–28.8)
Global haplotype association *p*-value: 0.27										

^1^ Rare (frequency ≤ 0.01) haplotypes with a summarized frequency; ^2^ Odds ratio with 95% confidence intervals adjusted for sex and age. Statistically significant associations are bolded; haplotypes significantly associated with IS risk are highlighted by gray.

**Table 4:** Associations of *GCLM* haplotypes with ischemic stroke stratified by smoking status.

*GCLM* Haplotype Frequencies Estimation in NonSmokers (*n* = 785)							
Haplotypes	rs7517826	rs3827715	rs2301022	Healthy Controls	IS Patients	*p*-Value	_adj_OR (95%CI) ^2^
*H1*	C	T	C	0.3266	0.4104	-	1.00
*H2*	**A**	**C**	**C**	0.2852	0.2447	**0.0047**	**0.65 (0.49–0.88)**
*H3*	**C**	**T**	**T**	0.2845	0.2095	**3 × 10^−4^**	**0.58 (0.43–0.78)**
*H4*	A	T	C	0.0782	0.0897	0.59	0.89 (0.57–1.37)
*H5*	A	C	T	0.0212	0.0327	0.56	1.29 (0.56–2.96)
*H6*	A	T	T	0.0029	0.0095	0.30	2.77 (0.40–19.16)
Rare ^1^	*	*	*	0.0014	0.0035		
Global haplotype association *p*-value: **0.0027**							
***GCLM* haplotype frequencies estimation in smokers (*n* = 485)**							
*H1*	C	T	C	0.4079	0.3842	-	1.00
*H2*	A	C	C	0.2307	0.2542	0.52	1.13 (0.78–1.64)
*H3*	C	T	T	0.2384	0.2531	0.46	1.15 (0.80–1.65)
*H4*	A	T	C	0.0678	0.0767	0.59	1.19 (0.64–2.23)
*H5*	A	C	T	0.0194	0.0170	0.89	1.11 (0.27–4.48)
*H6*	A	T	T	0.0358	0.0148	0.27	0.52 (0.16–1.64)
Global haplotype association *p*-value: 0.83							

^1^ Rare (frequency ≤ 0.01) haplotypes with a summarized frequency; ^2^ Odds ratio with 95% confidence intervals adjusted for sex and age. Statistically significant associations are bolded; haplotypes significantly associated with IS risk are highlighted by gray.

**Table 5:** Linkage disequilibrium measures between SNPs of the *GCLC* gene in the Russian population and populations of the 1000 Genomes Project.

SNP	rs12524494 (A > G)	rs636933 (G > A)	rs648595 (G > T)	rs761142 C > A	rs606548 (C > T)	rs17883901 (G > A)
rs12524494 (A > G)		**−0.0073**	**0.0186**	**0.0235**	**0.0285**	−0.0018
		**0.0091**	**−0.0331**	**0.0427**	**0.0511**	0.0020
		**0.0259**	**−0.0653**	**0.0948**	**0.1103**	**0.0070**
rs636933 (G > A)			**0.1190**	**0.1358**	**−0.0060**	**0.0133**
			**0.1190**	**−0.1280**	**0.0110**	**0.0090**
			**0.0445**	**−0.0852**	**0.0355**	**0.0130**
rs648595 (G > T)	*D*			**0.1360**	**0.0192**	**0.0110**
				**−0.1453**	**−0.0321**	**0.0135**
				**−0.1458**	**−0.0817**	**0.0039**
rs761142 C > A					**0.0254**	**0.0136**
					**0.0427**	**−0.0095**
					**0.1153**	**−0.0044**
rs606548 (C > T)						**−0.0025**
						0.0020
						**0.0121**
SNP	rs12524494	rs636933	rs648595	rs761142	rs606548	rs17883901
rs12524494 (A > G)		**0.9907**	**0.8955**	**0.8603**	**0.8862**	0.6553
		**0.7522**	**1.0000**	**0.9773**	**0.9114**	0.4012
		**0.9701**	**0.8450**	**0.8361**	**0.8289**	**0.5638**
rs636933 (G > A)			**1.0000**	**0.8640**	**0.8669**	**0.2151**
			**1.0000**	**0.8655**	**0.9174**	**0.1350**
			**1.0000**	**0.9008**	**0.9269**	**0.2219**
rs648595 (G > T)	*D’*			**0.9888**	**0.9967**	**0.2439**
				**0.9799**	**0.9700**	**0.2917**
				**0.9206**	**0.7383**	**0.1301**
rs761142 C > A					**0.9975**	**0.2291**
					**0.9773**	**0.1550**
					**0.7108**	**0.1011**
rs606548 (C > T)						**0.9698**
						0.4012
						**0.6841**

Matrices show LD measures, such as a nonstandardized *D* (upper part) and a standardized *D’* (lower part). LD-values were calculated with the LDpair Tool (https://ldlink.nci.nih.gov (accessed on 2 July 2021)) using genotype data from the 1000 Genomes Project (1000G) and GRCh37 human genome assembly. Each pair of SNPs includes three LD-values calculated for the following populations: the Russian population (upper cells), the European populations of 1000G (middle cells), and a mixed population of 1000G (lower cells). Cell color depicts a sign of LD between variants: red represents positive LD; blue represents negative LD. *D*-values that differed between Russian and other populations are circulated by the blue lines. Significant LD-values (*p* < 0.05) are highlighted.

**Table 6:** Linkage disequilibrium measures between SNPs of the *GCLM* gene in the Russian population and populations of the 1000 Genomes Project.

SNP	rs7517826 (C > A)	rs3827715 (T > C)	rs2301022 (C > T)
rs7517826 (C > A)	*D*	**0.1699**	**−0.0730**
		**0.1578**	**0.0778**
		**0.1289**	−0.0035
rs3827715 (T > C)			**−0.0587**
			**0.0661**
			**0.0487**
SNP	rs7517826	rs3827715	rs2301022
rs7517826 (C > A)	*D’*	**0.9930**	**0.6658**
		**0.9937**	**0.6509**
		**0.9985**	0.0155
rs3827715 (T > C)			**0.7374**
			**0.7778**
			**0.5675**

Matrices show linkage disequilibrium (LD) measures, such as a nonstandardized *D* (upper part) and a standardized *D’* (lower part). LD-values were calculated with the LDpair Tool (https://ldlink.nci.nih.gov (accessed on 2 July 2021)) using genotype data from the 1000 Genomes Project (1000G) and GRCh37 human genome assembly. Each pair of SNPs includes three LD-values calculated for the following populations: the Russian population (upper cells), the European populations of 1000G (middle cells), and a mixed population of 1000G (lower cells). Cell color depicts a sign of LD between variants: red represents positive LD; blue represents negative LD. *D*-values that differed between Russian and other populations are circulated by the blue lines. LD-values that are significant (*p* < 0.05) are highlighted.

**Table 7:** Replication analysis for associations of *GCLC* and *GCLM* gene polymorphisms with ischemic stroke in independent cohorts.

Gene, Effective Allele	Stroke Phenotype	*p*-Value	Beta/Odds Ratio	Dataset	Sample Size
*GCLC* rs12524494-G	TOAST large artery atherosclerosis	0.006	▲ 2.9874	MEGASTROKE GWAS	230, 076
		0.049	▲ 3.0648	MEGASTROKE GWAS (EUR)	190, 513
		0.26	▲ 1.4612	CADISP 2015	9, 326
		0.92	▼ 0.9674	VHIR FMT 2018	783
	All ischemic stroke	0.38	▲ 2.7532	MEGASTROKE GWAS	481, 992
		0.13	▲ 2.8174	MEGASTROKE GWAS (EUR)	404, 881
		0.09	▲ 1.2628	CADISP 2015	9, 814
		0.70	▼ 0.9140	VHIR FMT 2018	783
	Transient cerebral ischemic attacks and related syndromes	0.136	▲ 1.10	UK BIOBANK	452, 264
	Stroke, not specified as hemorrhage or infarction	0.0017	▼ 0.745	UK BIOBANK	452, 264
*GCLC* rs17883901-A	TOAST large artery atherosclerosis	0.16	▲ 2.8613	MEGASTROKE GWAS	227, 794
		0.23	▲ 2.8871	MEGASTROKE GWAS (EUR)	192, 425
		0.08	▲ 1.7191	CADISP 2015	9, 326
		0.06	▲ 2.1453	VHIR FMT 2018	783
	All ischemic stroke	0.09	▲ 2.7946	MEGASTROKE GWAS	475, 907
		0.74	▲ 2.7366	MEGASTROKE GWAS (EUR)	403, 224
		0.23	▲ 1.1652	CADISP 2015	9, 814
		0.023	▲ 1.6958	VHIR FMT 2018	783
	Transient cerebral ischemic attacks and related syndromes	0.25	▲ 1.06 (G)	UK BIOBANK	452, 264
	Stroke, not specified as hemorrhage or infarction	0.09	▼ 0.882 (G)	UK BIOBANK	452, 264
*GCLC* rs606548-T	TOAST large artery atherosclerosis	0.055	▲ 2.8984	MEGASTROKE GWAS	229, 842
		0.036	▲ 3.1030	MEGASTROKE GWAS (EUR)	189, 632
		0.10	▲ 1.7444	CADISP 2015	9, 326
		0.39	▼ 0.7489	VHIR FMT 2018	783
	All ischemic stroke	0.35	▲ 2.7541	MEGASTROKE GWAS	472, 735
		0.012	▲ 2.8929	MEGASTROKE GWAS (EUR)	395, 530
		0.039	▲ 1.3340	CADISP 2015	9, 814
		0.42	▼ 0.8337	VHIR FMT 2018	783
	Transient cerebral ischemic attacks and related syndromes	0.34	▲ 1.06	UK BIOBANK	452, 264
	Stroke, not specified as hemorrhage or infarction	0.002	▼ 0.746	UK BIOBANK	452, 264
* *GCLC* rs636933-A	TOAST large artery atherosclerosis	0.31	▲ 2.7857	MEGASTROKE GWAS	241, 607
		0.37	▲ 2.7900	MEGASTROKE GWAS (EUR)	203, 144
		0.33	▼ 0.8116	CADISP 2015	9, 326
		0.89	▲ 1.0272	VHIR FMT 2018	783
	All ischemic stroke	0.70	▲ 2.7077	MEGASTROKE GWAS	509, 234
		0.38	▲ 2.6911	MEGASTROKE GWAS (EUR)	432, 044
		0.29	▼ 0.9216	CADISP 2015	9, 814
		0.24	▲ 1.1540	VHIR FMT 2018	783
	Transient cerebral ischemic attacks and related syndromes	0.53	▲ 1.02 (G)	UK BIOBANK	452, 264
	Stroke, not specified as hemorrhage or infarction	0.51	▼ 0.97 (G)	UK BIOBANK	452, 264
* GCLC rs648595-T	TOAST large artery atherosclerosis	0.06	▲ 2.8283	MEGASTROKE GWAS	241, 442
		0.23	▲ 2.8026	MEGASTROKE GWAS (EUR)	201, 232
		0.61	▲ 1.0952	CADISP 2015	9, 326
		0.08	▼ 0.7558	VHIR FMT 2018	783
	All ischemic stroke	0.46	▲ 2.7358	MEGASTROKE GWAS	500, 913
		0.33	▲ 2.7455	MEGASTROKE GWAS (EUR)	423, 708
		0.93	▲ 1.0056	CADISP 2015	9, 814
		0.73	▲ 1.0362	VHIR FMT 2018	783
	Transient cerebral ischemic attacks and related syndromes	0.93	▲ 1.00 (G)	UK BIOBANK	452, 264
	Stroke, not specified as hemorrhage or infarction	0.80	▲ 1.01 (G)	UK BIOBANK	452, 264
* GCLC rs761142-C	TOAST large artery atherosclerosis	0.04	▲ 2.8442	MEGASTROKE GWAS	240, 561
		0.14	▲ 2.8359	MEGASTROKE GWAS (EUR)	200, 351
		0.77	▼ 0.9415	CADISP 2015	9, 326
		0.78	▼ 0.9510	VHIR FMT 2018	783
	All ischemic stroke	0.63	▲ 2.7064	MEGASTROKE GWAS	499, 208
		0.65	▲ 2.7042	MEGASTROKE GWAS (EUR)	422, 020
		0.89	▼ 0.9899	CADISP 2015	9, 814
		0.27	▲ 1.1348	VHIR FMT 2018	783
	Transient cerebral ischemic attacks and related syndromes	0.89	▲ 1.00	UK BIOBANK	452, 264
	Stroke, not specified as hemorrhage or infarction	0.48	▼ 0.97	UK BIOBANK	452, 264
* GCLM rs2301022-T	TOAST large artery atherosclerosis	0.99	▲ 2.7172	MEGASTROKE GWAS	242, 987
		0.74	▲ 2.6948	MEGASTROKE GWAS (EUR)	203, 144
		0.96	▲ 1.0106	CADISP 2015	9, 326
		0.03	▲ 1.4255	VHIR FMT 2018	783
	All ischemic stroke	0.73	▲ 2.7099	MEGASTROKE GWAS	511, 623
		0.19	▲ 2.6808	MEGASTROKE GWAS (EUR)	434, 418
		0.07	▲ 1.1338	CADISP 2015	9, 814
		0.07	▲ 1.2157	VHIR FMT 2018	783
	Transient cerebral ischemic attacks and related syndromes	0.08	▲ 1.05 (C)	UK BIOBANK	452, 264
	Stroke, not specified as hemorrhage or infarction	0.12	▼ 0.93 (C)	UK BIOBANK	452, 264
*GCLM* rs3827715-C	TOAST large artery atherosclerosis	0.49	▲ 2.7639	MEGASTROKE GWAS	242, 987
		0.74	▲ 2.7441	MEGASTROKE GWAS (EUR)	203, 144
		0.79	▲ 1.0540	CADISP 2015	9, 326
		0.15	▲ 1.2955	VHIR FMT 2018	783
	All ischemic stroke	0.55	▲ 2.7341	MEGASTROKE GWAS	511, 561
		0.97	▲ 2.7169	MEGASTROKE GWAS (EUR)	434, 418
		0.45	▲ 1.0572	CADISP 2015	9, 814
		0.02	▲ 1.3013	VHIR FMT 2018	783
	Transient cerebral ischemic attacks and related syndromes	0.26	▲ 1.03	UK BIOBANK	452, 264
	Stroke, not specified as hemorrhage or infarction	0.32	▲ 1.05	UK BIOBANK	452, 264
*GCLM* rs7517826-A	TOAST large artery atherosclerosis	0.88	▲ 2.7091	MEGASTROKE GWAS	241, 442
		0.45	▲ 2.6655	MEGASTROKE GWAS (EUR)	201, 232
		0.65	▲ 1.0871	CADISP 2015	9, 326
		0.06	▲ 1.3540	VHIR FMT 2018	783
	All ischemic stroke	0.59	▲ 2.7053	MEGASTROKE GWAS	503, 288
		0.61	▲ 2.7040	MEGASTROKE GWAS (EUR)	426, 083
		0.98	▼ 0.9982	CADISP 2015	9, 814
		0.026	▲ 1.2603	VHIR FMT 2018	783
	Transient cerebral ischemic attacks and related syndromes	0.019	▲ 1.07	UK BIOBANK	452, 264
	Stroke, not specified as hemorrhage or infarction	0.67	▲ 1.02	UK BIOBANK	452, 264

Genomic data obtained at the Cerebrovascular Disease Knowledge Portal (https://cd.hugeamp.org (accessed on 26 March 2022)). *p*-values reached significance level (*p* ≤ 0.05) are bolded. *—SNPs that showed associations with ischemic stroke or brain infarct size in the present study. ▲ Ndepicts an increased value, ▼ Hdepicts a decreased value

**Table 8:** The best four *n*-order gene–gene and gene–environment interactions significantly associated with the risk of ischemic stroke.

Gene–Gene and Gene–Environment Interactions		NH	*β* H	WH	NL	*β* L	WL	*P*_perm_
**Two-order GxG/GxE interactions**								
1	*GCLM* rs2301022 × *RASEF* rs4322086	3	0.175	37.70	4	−0.176	29.71	**<0.001**
2	SMOKE × ALCOHOL	2	0.175	30.54	1	−0.176	32.33	**<0.001**
3	SMOKE × VEGET	2	0.176	30.79	1	−0.147	20.61	**<0.001**
4	*RASEF* rs4322086 × SMOKE	1	0.199	30.24	1	−0.141	16.50	**<0.001**
**Three-order GxG/GxE interactions**								
1	*GCLM* rs2301022 × *RASEF* rs4322086 × LOC105370913 rs899997	3	0.173	29.22	6	−0.248	45.37	**<0.001**
2	*GCLM* rs3827715 × *GCLM* rs2301022 × *RASEF* rs4322086	3	0.143	17.27	5	−0.240	45.12	**<0.001**
3	*GCLC* rs648595 × *RASEF* rs4322086 × *ZC3HC1* rs11556924	4	0.234	43.84	1	−0.170	8.47	**<0.001**
4	*GCLM* rs2301022 × *LDLR* rs6511720 × *RASEF* rs4322086	5	0.195	43.54	4	−0.195	31.64	**<0.001**
**Four-order GxG/GxE interactions**								
1	*GCLM* rs2301022 × *RASEF* rs4322086 × LOC105370913 rs899997 × SMOKE	6	0.229	36.43	9	−0.273	59.01	**<0.002**
2	*GCLM* rs3827715 × *GCLM* rs2301022 × *GCLC* rs761142 × *RASEF* rs4322086	7	0.252	46.33	10	−0.260	55.87	**<0.002**
3	*GCLM* rs2301022 × *GCLC* rs606548 × *RASEF* rs4322086 × SMOKE	6	0.257	55.05	5	−0.180	26.83	**<0.002**
4	*GCLC* rs648595 × *LDLR* rs6511720 × *RASEF* rs4322086 × *ZC3HC1* rs11556924	6	0.272	54.15	5	−0.219	24.02	**<0.002**
**Five-order GxG/GxE interactions**								
1	*GCLM* rs3827715 × *GCLC* rs17883901 × *GCLC* rs12524494 ALCOHOL × SMOKE	1	0.093	3.43	5	−0.139	19.42	**0.01**
2	*GCLC* rs636933 × *RASEF* rs4322086 × *SLCO1B1* rs2417957 × *PITX2* rs12646447 × VEGET	1	0.159	8.08	3	−0.237	19.37	**0.01**
3	*GCLM* rs2301022 × *GCLC* rs12524494 × *AIM1* rs783396 × *SLCO1B1* rs2417957 × VEGET	3	0.142	12.34	4	−0.155	19.36	**0.01**
4	*GCLM* rs7517826 × *GCLC* rs606548 × *GCLC* rs12524494 × *PEMT* rs12449964 × VEGET	1	0.237	4.65	7	−0.215	19.35	**0.01**

Models are obtained using the model-based multifactor dimensionality reduction method, MB-MDR package for R. *β* H, regression coefficient for high-risk exposition in the step 2 analysis; *β* L, regression coefficient for low-risk exposition in the step 2 analysis; NH, number of significant high-risk genotypes in the interaction; NL, number of significant low-risk genotypes in the interaction; *P*_perm_, permutation *p*-value for the interaction model. The models were adjusted for age and sex; WH, Wald statistic for the high-risk category; WL, Wald statistic for the low-risk category. Environment risk factors: SMOKE, cigarette smoking; ALCOHOL, alcohol abuse; VEGET, low vegetables/fruits intake.

**Table 9:** Post hoc analysis of associations between the risk of ischemic stroke and diplotypes of the lead SNPs presented in the two-order. GxG *mbmdr*-models.

№	Genotype Combinations	IS Patients		Controls		OR (95% CI) ^1^	*p* ^ 2^	*FDR* ^ 3^
		n	%	n	%			
1	*RASEF* rs4322086-G/G × *GCLM* rs2301022-C/C	43	7.9	56	8.7	0.89 (0.59–1.35)	0.599	0.63
2	*RASEF* rs4322086-G/G × *GCLM* rs2301022-C/T	45	8.3	31	4.8	1.77 (1.10–2.84)	**0.017**	**0.04**
3	*RASEF* rs4322086-G/G × *GCLM* rs2301022-T/T	4	0.7	16	2.5	0.29 (0.10–0.87)	**0.019**	**0.04**
4	*RASEF* rs4322086-G/A × *GCLM* rs2301022-C/C	157	28.8	137	21.4	1.49 (1.14–1.94)	**0.003**	**0.015**
5	*RASEF* rs4322086-G/A × *GCLM* rs2301022-C/T	127	23.3	106	16.5	1.53 (1.15–2.04)	**0.003**	**0.015**
6	*RASEF* rs4322086-G/A × *GCLM* rs2301022-T/T	14	2.6	38	5.9	0.42 (0.22–0.78)	**0.005**	**0.015**
7	*RASEF* rs4322086-A/A × *GCLM* rs2301022-C/C	80	14.7	135	21.1	0.64 (0.48–0.87)	**0.004**	**0.015**
8	*RASEF* rs4322086-A/A × *GCLM* rs2301022-C/T	67	12.3	97	15.1	0.79 (0.56–1.10)	0.158	0.26
9	*RASEF* rs4322086-A/A × *GCLM* rs2301022-T/T	8	1.5	25	3.9	0.38 (0.17–0.84)	**0.020**	**0.04**
10	*PEMT* rs12449964-C/C × *GCLM* rs2301022-C/C	109	20.1	124	18.7	1.10 (0.82–1.46)	0.528	0.59
11	*PEMT* rs12449964-C/C × *GCLM* rs2301022-C/T	92	17.0	87	13.1	1.36 (0.99–1.86)	0.060	0.11
12	*PEMT* rs12449964-C/C × *GCLM* rs2301022-T/T	11	2.0	34	5.1	0.38 (0.19–0.77)	0.005	0.015
13	*PEMT* rs12449964-C/T × *GCLM* rs2301022-C/C	139	25.7	164	24.7	1.05 (0.81–1.37)	0.703	0.70
14	*PEMT* rs12449964-C/T × *GCLM* rs2301022-C/T	113	20.9	125	18.9	1.14 (0.86–1.51)	0.378	0.49
15	*PEMT* rs12449964-C/T × *GCLM* rs2301022-T/T	9	1.7	42	6.3	0.26 (0.13–0.53)	**0.0001**	**0.0018**
16	*PEMT* rs12449964-T/T × *GCLM* rs2301022-C/C	32	5.9	52	7.8	0.74 (0.47–1.17)	0.191	0.29
17	*PEMT* rs12449964-T/T × *GCLM* rs2301022-C/T	30	5.5	31	4.7	1.20 (0.71–2.00)	0.494	0.59
18	*PEMT* rs12449964-T/T × *GCLM* rs2301022-T/T	6	1.1	4	0.6	1.78 (0.53–5.95)	0.336	0.47

^1^ Unadjusted odds ratio for the association between a genotype combination and the risk of ischemic stroke; ^2^ Significance level for the association between a genotype combination and the risk of ischemic stroke; Bold type indicates statistically significant differences in genotype combinations between the study groups. ^3^ FDR, false discovery rate.

**Table 10:** The best four *n*-order gene–gene and gene–environment interactions significantly associated with brain infarct size.

Gene–Gene and Gene–Environment Interactions		NH	*β* H	WH	NL	*β* L	WL	*P*_perm_
**Two-order GxG/GxE interactions**								
1	*RASEF* rs4322086 × SMOKE	1	3.968	13.93	1	−2.375	5.41	**0.002**
2	*RASEF* rs4322086 × *ZC3HC1* rs11556924	2	3.863	16.19	2	−2.952	8.11	**0.005**
3	*RASEF* rs4322086 × *GCLM* rs2301022	2	3.239	14.65	2	−2.589	5.99	**0.008**
4	*SLCO1B1* rs2417957 × *GCLC* rs648595	2	21.281	19.80	0	-	-	**0.009**
**Three-order GxG/GxE interactions**								
1	*PITX2* rs12646447 × *ZC3HC1* rs11556924 × *GCLC* rs648595	3	27.429	38.68	0	-	-	**0.001**
2	*ZC3HC1* rs11556924 × *GCLC* rs648595 × ALCOHOL	3	9.974	26.38	0	-	-	**0.001**
3	*PITX2* rs12646447 × *PEMT* rs12449964 × *GCLC* rs648595	2	44.033	30.18	0	-	-	**0.002**
4	*RASEF* rs4322086 × *PITX2* rs12646447 × SMOKE	4	6.681	32.73	2	−3.749	9.84	**0.004**
**Four-order GxG/GxE interactions**								
1	*GCLM* rs3827715 × *GCLC* rs636933 × *PITX2* rs12646447 × *ZC3HC1* rs11556924	4	28.155	71.44	0	-	-	**<0.002**
2	*GCLC* rs648595 × *SLCO1B1* rs2417957 × *PITX2* rs12646447 × *ZC3HC1* rs11556924	5	28.427	56.95	1	−7.303	2.77	**<0.002**
3	*GCLC* rs648595 × *GCLC* rs606548 × *PITX2* rs12646447 × *ZC3HC1* rs11556924	5	21.454	54.06	0	-	-	**<0.002**
4	*GCLC* rs648595 × *PEMT* rs12449964 × *ZC3HC1* rs11556924 × ALCOHOL	5	17.308	53.83	0	-	-	**<0.002**
**Five-order GxG/GxE interactions**								
1	*GCLC* rs636933 × *GCLC* rs12524494 × *AIM1* rs783396 × *SLCO1B1* rs2417957 × *PITX2* rs12646447	4	14.795	18.69	1	−3.124	2.74	**0.01**
2	*GCLC* rs606548 × *GCLC* rs648595 × *GCLC* rs17883901 × *SLCO1B1* rs2417957 × *PITX2* rs12646447	6	7.699	18.65	1	−6.380	3.41	**0.01**
3	*GCLC* rs606548 × *SLCO1B1* rs2417957 × *ZC3HC1* rs11556924 × VEGET × ALCOHOL	3	35.186	18.23	1	−2.748	2.81	**0.01**
4	*GCLC* rs606548 × *GCLC* rs12524494 × *GCLC* rs17883901 × *PEMT* rs12449964 × ALCOHOL	4	10.448	18.10	1	−3.016	4.53	**0.01**

Models are obtained using the model-based multifactor dimensionality reduction method, MB-MDR package for R. *β* H, regression coefficient for high-risk exposition in the step 2 analysis; *β* L, regression coefficient for low-risk exposition in the step 2 analysis; NH, number of significant high-risk genotypes in the interaction; NL, number of significant low-risk genotypes in the interaction; *P*_perm_, permutation *p*-value for the interaction model. The models were adjusted for age and sex; WH, Wald statistic for the high-risk category; WL, Wald statistic for the low-risk category. Environment risk factors: SMOKE, cigarette smoking; ALCOHOL, alcohol abuse; VEGET, low vegetables/fruits intake.

**Table 11:** Summarized data on functional annotations of *GCLC* and *GCLM* gene polymorphisms using various bioinformatics tools.

Gene	SNP ID	Alleles	Location in the Gene	Regulatory Potential		Expression Levels (eQTL Analysis)							Epigenetic Regulation								TFBS		
				FuncPred	Regulome Score	Blood/ Blood Cells			Arteries, Aorta		Brain Tissues		Histone Marks	Open Chromatin	CTCF Binding	Promoter	Promoter Flanking region	DNA Methylation			VEP	Transfac	atSNP
						GTEx	eQTLGen	QTLbase	GTEx	QTLbase	GTEx	QTLbase						Blood	Arteries, Aorta	Brain Tissues			
*GCLC*	rs12524494	A/G	intron	0.000	6		**√**	**√**					**√**										**√**
	rs17883901	G/A	intron	0.249	3a								**√**	**√**	**√**	**√**		**√**			**√**	**√**	**√**
	rs606548	C/T	intron	0.000	5		**√**	**√**													**√**		**√**
	rs636933	G/A	intron	-	-		**√**	**√**			**√**												**√**
	rs648595	G/T	intron	0.187	5		**√**	**√**			**√**	**√**											**√**
	rs761142	A/C	intron	0.000	5		**√**	**√**			**√**	**√**	**√**	**√**									**√**
*GCLM*	rs2301022	C/T	intron	0.000	4		**√**	**√**				**√**	**√**					**√**		**√**			**√**
	rs3827715	T/C	intron	0.000	5		**√**	**√**	**√**			**√**	**√**				**√**	**√**		**√**	**√**		**√**
	rs7517826	C/A	intron	0.000	-		**√**	**√**	**√**	**√**	**√**	**√**						**√**		**√**			**√**

Detailed information on the usage of both bioinformatics tools is described in the Methods Section. TFBS, transcription factor binding site.

## Data Availability

Data supporting reported results are available upon request.

## Supplementary Materials

The following supporting information can be downloaded at: https://www.mdpi.com/article/10.3390/life12040602/s1, Table S1: Statistics for all mbmdr-models of GxG and GxE interactions associated with the risk of ischemic stroke; Table S2: Statistics for the best mbmdr-models of GxG and GxE interactions associated with the risk of ischemic stroke; Table S3: Statistics for all mbmdr-models of GxG and GxE interactions associated with brain infarct size; Table S4: Statistics for the best mbmdr-models of GxG and GxE interactions associated with brain infarct size; Table S5: The results of cis eQTL data analysis for SNPs of *GCLC* and *GCLM* genes; Table S6: The results of various molecular trait QTL data analysis for SNPs of *GCLC* and *GCLM* genes; Table S7: Data on epigenetic regulatory potential of *GCLM* and *GCLC* gene polymorphisms; Table S8: A list of transcription factors predicted by atSNP Search whose binding sites are located in the regions around SNPs of *GCLM* and *GCLC* genes.
