# Supplementary material for: The Impact of Genetic Polymorphisms in Glutamate-Cysteine Ligase, a Key Enzyme of Glutathione Biosynthesis, on Ischemic Stroke Risk and Brain Infarct Size
Source: Life (Basel). 2022 Apr 18;12(4):602. doi: 10.3390/life12040602 (PMC9032935; doi:10.3390/life12040602)
Supplement: Supplementary file 1 [file life-12-00602-s001.zip › Supplementary table S5.pdf]

The results of *cis* eQTL data analysis for SNPs of *GCLC* and *GCLM* genes

SNP ID	Effective Allele	Effect size	Z-score	P-value	FDR	Tissue	Database
rs12524494	G	NA	-8.59	8.5023e-18	0	Blood	eQTLGen
rs12524494	NA	-0.14	NA	2.100e-6	2.640e-4	Blood	QTLbase
rs17883901	A	0.24	NA	0.000001	NA	Adipose - Visceral	GTEx
rs17883901	A	0.21	NA	0.000009	NA	Adipose - Subcutaneous	GTEx
rs17883901	G	-1.07	NA	3.840e-26	NA	Adipose-Subcutaneous	QTLbase
rs606548	T	NA	-8.04	8.7809e-16	0	Blood	eQTLGen
rs606548	T	NA	NA	8.780e-16	0	Blood	QTLbase
rs636933	NA	-0.10	NA	1.020e-7	1.620e-5	Blood	QTLbase
rs636933	A	-0.16	NA	2.3e-7	NA	Testis	GTEx
rs636933	A	-0.23	NA	9.5e-7	NA	Skin - Not Sun Exposed	GTEx
rs636933	A	-0.32	NA	0.0000016	NA	Adrenal Gland	GTEx
rs636933	A	0.24	NA	0.000036	NA	Pancreas	GTEx
rs636933	A	-0.25	NA	0.000051	NA	Brain - Caudate (basal ganglia)	GTEx
rs636933	A	NA	-10.63	2.1381e-26	0	Blood	eQTLGen
rs648595	G	NA	NA	1.160e-70	NA	Blood	QTLbase
rs648595	NA	NA	NA	2.960e-31	NA	Brain-Prefrontal Cortex	QTLbase
rs648595	G	-0.43	NA	6.020e-14	NA	Adrenal Gland	QTLbase
rs648595	NA	0.04	NA	1.280e-7	9.580e-6	Brain-Prefrontal Cortex	QTLbase
rs648595	NA	0.36	NA	1.360e-7	9.510e-5	Liver	QTLbase
rs648595	G	-0.09	NA	8.720e-7	0.0058	Blood	QTLbase
rs648595	G	-0.21	NA	4.210e-6	NA	Brain-Hippocampus	QTLbase
rs648595	G	-0.43	NA	1.6e-15	NA	Adrenal Gland	GTEx
rs648595	G	-0.18	NA	1.1e-12	NA	Testis	GTEx
rs648595	G	-0.23	NA	4.3e-9	NA	Skin - Not Sun Exposed	GTEx
rs648595	G	-0.21	NA	1.1e-7	NA	Nerve - Tibial	GTEx
rs648595	G	0.23	NA	2.5e-7	NA	Pancreas	GTEx
rs648595	G	-0.18	NA	2.8e-7	NA	Brain - Hippocampus	GTEx
rs648595	G	-0.21	NA	0.0000013	NA	Brain - Cortex	GTEx
rs648595	G	-0.11	NA	0.000010	NA	Adipose - Subcutaneous	GTEx
rs648595	G	-0.24	NA	0.000016	NA	Brain - Caudate (basal ganglia)	GTEx
rs648595	G	-0.10	NA	0.000020	NA	Thyroid	GTEx
rs648595	G	-0.15	NA	0.000035	NA	Skin - Sun Exposed	GTEx
rs648595	G	-0.13	NA	0.00012	NA	Lung	GTEx
rs648595	G	NA	-17.80	1.1646e-70	0	Blood	eQTLGen
rs761142	C	NA	-15.04	4.2399e-51	0	Blood	eQTLGen
rs761142	C	NA	NA	4.240e-51	NA	Blood	QTLbase
rs761142	NA	NA	NA	5.440e-18	NA	Brain-Prefrontal Cortex	QTLbase
rs761142	NA	0.19	NA	2.040e-9	NA	Liver	QTLbase
rs761142	C	-0.35	NA	3.970e-8	NA	Adrenal Gland	QTLbase
rs761142	C	-0.10	NA	5.210e-7	0.0038	Blood	QTLbase
rs761142	NA	-0.60	NA	5.600e-5	NA	Blood-Monocyte	QTLbase
rs761142	NA	-0.59	NA	6.440e-5	NA	Blood-T cell CD8+ naive	QTLbase
rs761142	NA	0.03	NA	5.010e-4	0.0155	Brain-Prefrontal Cortex	QTLbase
rs761142	C	-0.15	NA	5.620e-4	NA	Brain	QTLbase
rs761142	C	-0.37	NA	9.8e-10	NA	Adrenal Gland	GTEx
rs761142	C	-0.20	NA	8.8e-7	NA	Skin - Not Sun Exposed	GTEx
rs761142	C	-0.14	NA	0.0000012	NA	Testis	GTEx
rs761142	C	-0.27	NA	0.0000019	NA	Brain - Caudate (basal ganglia)	GTEx
rs761142	C	-0.19	NA	0.0000022	NA	Brain - Hippocampus	GTEx
rs761142	C	-0.23	NA	0.0000043	NA	Brain - Cortex	GTEx
rs761142	C	-0.23	NA	0.000033	NA	Brain - Putamen (basal ganglia)	GTEx
rs2301022	C	-0.20	NA	2.000e-6	NA	Lymphocyte	QTLbase

rs2301022	NA	NA	NA	2.590e-5	NA	Brain-Prefrontal Cortex	QTLbase
rs2301022	T	NA	NA	1.950e-4	NA	Blood	QTLbase
rs2301022	NA	-0.39	NA	2.120e-4	0.0019	Blood-Neutrophils CD16+	QTLbase
rs2301022	G	NA	NA	4.990e-4	NA	Blood	QTLbase
rs2301022	T	NA	NA	8.350e-4	NA	Blood	QTLbase
rs2301022	C	0.20	NA	2.1e-7	NA	Esophagus - Mucosa	GTEx
rs2301022	C	-0.15	NA	5.9e-7	NA	Cells - Cultured fibroblasts	GTEx
rs2301022	C	0.11	NA	0.00012	NA	Skin - Sun Exposed	GTEx
rs3827715	C	-0.25	NA	1.7e-20	NA	Cells - Cultured fibroblasts	GTEx
rs3827715	C	-0.17	NA	6.2e-11	NA	Adipose - Subcutaneous	GTEx
rs3827715	C	-0.21	NA	1.3e-10	NA	Testis	GTEx
rs3827715	C	-0.14	NA	1.1e-9	NA	Thyroid	GTEx
rs3827715	C	-0.19	NA	1.8e-9	NA	Nerve - Tibial	GTEx
rs3827715	C	-0.15	NA	0.0000016	NA	Muscle - Skeletal	GTEx
rs3827715	C	-0.15	NA	0.0000021	NA	Artery - Tibial	GTEx
rs3827715	C	-0.12	NA	0.000014	NA	Adipose - Visceral	GTEx
rs3827715	C	0.16	NA	0.000022	NA	Esophagus - Mucosa	GTEx
rs3827715	C	-0.16	NA	0.000033	NA	Heart - Atrial Appendage	GTEx
rs3827715	C	-0.19	NA	0.000044	NA	Pancreas	GTEx
rs3827715	C	-0.22	NA	0.000050	NA	Brain - Nucleus accumbens	GTEx
rs3827715	C	-0.16	NA	0.00011	NA	Artery - Aorta	GTEx
rs3827715	C	-0.12	NA	0.00018	NA	Esophagus - Muscularis	GTEx
rs3827715	C	NA	5.37	8.0057e-8	0.0003	Blood	eQTLGen
rs3827715	G	NA	NA	4.68E-06	NA	Blood	QTLbase
rs3827715	NA	0.26	NA	2E-07	NA	Blood	QTLbase
rs3827715	C	NA	NA	2.32E-08	NA	Blood	QTLbase
rs3827715	NA	0.52	NA	1.71E-17	NA	Blood	QTLbase
rs3827715	NA	0.14	NA	2.04E-08	6.09E-06	Brain-Prefrontal Cortex	QTLbase
rs3827715	NA	0.14	NA	5.73E-22	3.27E-19	Brain-Prefrontal Cortex	QTLbase
rs3827715	NA	-0.19	NA	0.0869	NA	Kidney	QTLbase
rs3827715	NA	0.27	NA	5.88E-05	NA	Skin	QTLbase
rs3827715	NA	0.48	NA	6.75E-11	NA	Skin	QTLbase
rs3827715	T	0.25	NA	1.67E-08	NA	Lymphocyte	QTLbase
rs3827715	T	0.41	NA	9.42E-17	NA	Lymphocyte	QTLbase
rs7517826	A	-0.24	NA	5.5e-20	NA	Cells - Cultured fibroblasts	GTEx
rs7517826	A	-0.17	NA	5.3e-12	NA	Adipose - Subcutaneous	GTEx
rs7517826	A	-0.19	NA	1.7e-10	NA	Nerve - Tibial	GTEx
rs7517826	A	-0.12	NA	1.7e-8	NA	Thyroid	GTEx
rs7517826	A	-0.17	NA	3.4e-8	NA	Artery - Tibial	GTEx
rs7517826	A	-0.16	NA	5.3e-8	NA	Muscle - Skeletal	GTEx
rs7517826	A	-0.13	NA	1.3e-7	NA	Adipose - Visceral	GTEx
rs7517826	A	-0.22	NA	1.3e-7	NA	Pancreas	GTEx
rs7517826	A	-0.20	NA	1.9e-7	NA	Heart - Atrial Appendage	GTEx
rs7517826	A	-0.26	NA	2.8e-7	NA	Brain - Nucleus accumbens	GTEx
rs7517826	A	-0.15	NA	6.7e-7	NA	Esophagus - Muscularis	GTEx
rs7517826	A	-0.15	NA	6.8e-7	NA	Testis	GTEx
rs7517826	A	-0.18	NA	0.0000086	NA	Artery - Aorta	GTEx
rs7517826	A	-0.24	NA	0.000029	NA	Brain - Putamen (basal ganglia)	GTEx
rs7517826	A	-0.19	NA	0.000031	NA	Pituitary	GTEx
rs7517826	A	-0.23	NA	0.00004	NA	Brain - Cerebellum	GTEx
rs7517826	A	0.14	NA	0.00012	NA	Esophagus - Mucosa	GTEx
rs7517826	A	NA	9.18	4.2711e-20	0	Blood	eQTLGen
rs7517826	C	0.49	NA	2.240e-29	NA	Lymphocyte	QTLbase
rs7517826	NA	NA	NA	1.890e-26	NA	Brain-Prefrontal Cortex	QTLbase
rs7517826	NA	0.55	NA	5.550e-24	NA	Blood	QTLbase

rs7517826	NA	0.13	NA	7.610e-23	4.610e-20	Brain-Prefrontal Cortex	QTLbase
rs7517826	A	NA	NA	4.270e-20	NA	Blood	QTLbase
rs7517826	A	NA	NA	2.170e-19	NA	Blood	QTLbase
rs7517826	NA	0.54	NA	1.090e-13	NA	Skin	QTLbase
rs7517826	C	0.28	NA	5.320e-12	NA	Lymphocyte	QTLbase
rs7517826	A	-0.23	NA	2.120e-11	NA	Fibroblast	QTLbase
rs7517826	NA	0.28	NA	4.360e-10	NA	Blood	QTLbase
rs7517826	NA	0.14	NA	7.940e-10	2.990e-7	Brain-Prefrontal Cortex	QTLbase
rs7517826	NA	-0.63	NA	8.830e-10	1.200e-7	Blood-Neutrophils CD16+	QTLbase
rs7517826	A	-0.19	NA	2.230e-8	NA	Peripheral Nervous System	QTLbase
rs7517826	A	0.15	NA	4.900e-8	NA	Blood	QTLbase
rs7517826	A	-0.26	NA	2.180e-7	NA	Artery-Aorta	QTLbase
rs7517826	A	-0.14	NA	2.640e-7	NA	Thyroid Gland	QTLbase
rs7517826	A	-0.14	NA	4.550e-7	NA	Adipose-Subcutaneous	QTLbase
rs7517826	NA	0.34	NA	4.590e-7	NA	Skin	QTLbase
rs7517826	A	-0.39	NA	6.340e-7	NA	Brain-Nucleus Accumbens	QTLbase
rs7517826	NA	-0.23	NA	7.200e-7	NA	Blood	QTLbase
rs7517826	A	-0.15	NA	1.220e-6	NA	Adipose-Visceral	QTLbase
rs7517826	A	-0.23	NA	2.720e-6	NA	Heart-Atrial Appendage	QTLbase
rs7517826	A	-0.17	NA	4.610e-6	NA	Muscle-Skeletal	QTLbase
rs7517826	A	-0.18	NA	9.760e-6	NA	Testis	QTLbase
rs7517826	NA	3.41	NA	7.310e-4	0.0326	Blood-Monocyte	QTLbase
rs7517826	C	-0.11	NA	8.440e-4	NA	Lymphocyte	QTLbase

NA, not available. eQTL in brain tissues and arteries are highlighted in yellow.
