# Supplementary material for: The Impact of Genetic Polymorphisms in Glutamate-Cysteine Ligase, a Key Enzyme of Glutathione Biosynthesis, on Ischemic Stroke Risk and Brain Infarct Size
Source: Life (Basel). 2022 Apr 18;12(4):602. doi: 10.3390/life12040602 (PMC9032935; doi:10.3390/life12040602)
Supplement: Supplementary file 1 [file life-12-00602-s001.zip › Supplementary table S6.pdf]

Supplementary table S6

The results of various molecular trait QTL data analysis for SNPs of *GCLC* and *GCLM* genes

SNP ID	QTL type	Molecular trait	Effective Allele	Effect size	P-value	FDR	Tissue
rs12524494	sQTL	GCLC	NA	-0.20	0.0024	NA	Kidney
rs17883901	mQTL	cg12013321	NA	0.48	4.120e-9	0.002	Blood
rs17883901	mQTL	cg12013321	NA	0.47	1.130e-8	0.004	Blood
rs636933	mQTL	cg02868790	A	-0.82	8.66E-33	NA	Prostate
rs636933	mQTL	cg02868790	A	-0.63	2.16E-17	NA	Large Intestine-Colon
rs636933	mQTL	cg02868790	NA	NA	1.96E-06	NA	Fibroblast
rs636933	sQTL	GCLC	NA	NA	3.7E-19	6.19E-17	Brain
rs636933	sQTL	GCLC	NA	-0.18	1.67E-07	NA	Breast
rs636933	sQTL	GCLC	NA	-0.32	2.28E-06	NA	Stomach
rs636933	sQTL	GCLC	NA	-0.21	4.16E-06	NA	Lung
rs636933	sQTL	GCLC	NA	-0.3	1.82E-05	NA	Esophagus
rs636933	sQTL	GCLC	NA	-0.25	6.44E-05	NA	Large Intestine-Colon
rs636933	sQTL	GCLC	NA	-0.22	0.000194	NA	Ovary
rs636933	sQTL	GCLC	NA	-0.17	0.000617	NA	Thyroid Gland
rs636933	sQTL	GCLC	NA	-0.14	0.000842	NA	Kidney
rs648595	mQTL	cg02868790	G	-0.84	6.35E-50	NA	Prostate
rs648595	mQTL	cg02868790	G	-0.46	8.92E-13	NA	Large Intestine-Colon
rs648595	mQTL	cg02868790	G	-0.3	1.63E-09	NA	Liver
rs648595	sQTL	GCLC	NA	-0.25	6.43E-19	NA	Breast
rs648595	sQTL	GCLC	NA	-0.25	1.44E-11	NA	Lung
rs648595	sQTL	GCLC	NA	-0.23	7.1E-11	NA	Kidney
rs648595	sQTL	GCLC	NA	-0.2	1.11E-09	NA	Lung
rs648595	sQTL	GCLC	NA	-0.29	2.43E-08	NA	Stomach
rs648595	sQTL	GCLC	NA	-0.21	3E-08	NA	Brain
rs648595	sQTL	GCLC	NA	-0.22	3.52E-08	NA	Thyroid Gland
rs648595	sQTL	GCLC	NA	-0.36	3.85E-07	NA	Pancreas
rs648595	sQTL	GCLC	NA	-0.28	2.06E-06	NA	Esophagus
rs648595	sQTL	GCLC	NA	-0.22	7.62E-06	NA	Ovary
rs648595	sQTL	GCLC	NA	-0.28	4.39E-05	NA	Adrenal Gland
rs761142	mQTL	cg02868790	C	-0.73	6.2E-28	NA	Prostate
rs761142	mQTL	cg02868790	C	-0.46	7.89E-11	NA	Large Intestine-Colon
rs761142	sQTL	GCLC	NA	-0.19	2.08E-09	NA	Breast
rs761142	sQTL	GCLC	NA	-0.32	3.08E-07	NA	Esophagus
rs761142	sQTL	GCLC	NA	-0.3	4.45E-07	NA	Stomach
rs761142	sQTL	GCLC	NA	-0.2	2.24E-06	NA	Thyroid Gland
rs761142	sQTL	GCLC	NA	-0.19	3.04E-06	NA	Kidney
rs761142	sQTL	GCLC	NA	-0.17	7.56E-05	NA	Lung
rs2301022	mQTL	cg16731318	C	0.77	6.63E-25	NA	Liver
rs2301022	mQTL	cg16731318	C	0.63	5.37E-22	NA	Thyroid Gland
rs2301022	mQTL	cg26688893	NA	0.285673	3.73E-13	2.75E-07	Blood
rs2301022	mQTL	cg16731318	C	0.49	5.2E-13	NA	Brain
rs2301022	mQTL	cg16731318	C	0.47	7.28E-11	NA	Stomach
rs2301022	mQTL	cg26688893	NA	0.238405	4.06E-09	0.00176	Blood
rs2301022	mQTL	cg16731318	C	0.53	4.76E-09	NA	Skin
rs2301022	mQTL	cg16731318	C	0.47	1.45E-08	NA	Kidney
rs2301022	mQTL	cg16731318	C	0.45	1.72E-07	NA	Cervix
rs2301022	mQTL	cg08237401	NA	0.5356	2.65E-07	3.39E-06	Blood-Monocytes CD14+
rs2301022	mQTL	cg16731318	C	0.64	7.91E-07	NA	Pancreas
rs2301022	mQTL	cg16731318	C	0.61	1.31E-06	NA	Esophagus
rs2301022	mQTL	cg16731318	C	0.65	6.6E-06	NA	Thymus
rs2301022	mQTL	cg22807700	NA	-0.4165	8.19E-05	0.00422	Blood-Monocytes CD14+
rs2301022	mQTL	cg16731318	NA	0.77	6.63E-25	NA	Liver

rs3827715	mQTL	cg16731318	C	-0.61	1.12E-27	NA	Breast
rs3827715	mQTL	cg16731318	C	-0.71	3.8E-23	NA	Prostate
rs3827715	mQTL	cg16731318	C	-0.58	2.05E-16	NA	Thyroid Gland
rs3827715	mQTL	cg16731318	C	-0.54	1.63E-15	NA	Brain
rs3827715	mQTL	cg16731318	C	-0.64	3.03E-15	NA	Stomach
rs3827715	mQTL	cg16731318	C	-0.70	8.26E-14	NA	Kidney
rs3827715	mQTL	cg16731318	C	-0.63	1.02E-12	NA	Kidney
rs3827715	mQTL	cg16731318	C	-0.55	2.17E-12	NA	Bladder
rs3827715	mQTL	cg13856647	C	0.01	5.77E-12	NA	Brain
rs3827715	mQTL	cg16731318	C	-0.59	1.03E-11	NA	Cervix
rs3827715	mQTL	cg16731318	C	-0.54	1.98E-11	NA	Lung
rs3827715	mQTL	cg16731318	C	-0.65	9.35E-11	NA	Skin
rs3827715	mQTL	cg13856647	C	0.44	1.06E-10	NA	Prostate
rs3827715	mQTL	cg15925478	NA	-0.29	1.93E-10	0.000101	Blood
rs3827715	mQTL	cg08237401	NA	0.31	2.47E-10	0.000111	Blood
rs3827715	mQTL	cg16731318	C	-0.56	3.5E-10	NA	Liver
rs3827715	mQTL	cg22807700	NA	-0.06	6.46E-10	NA	Brain-Cerebellum
rs3827715	mQTL	cg08237401	NA	0.32	2.38E-09	0.00109	Blood
rs3827715	mQTL	cg15925478	NA	-0.25	4.36E-09	0.00162	Blood
rs3827715	mQTL	cg16731318	C	-0.67	1.85E-08	NA	Pancreas
rs3827715	mQTL	cg08237401	NA	0.30	2.73E-08	0.0104	Blood
rs3827715	mQTL	cg22807700	NA	NA	3.71E-08	NA	Brain-Cerebellum
rs3827715	mQTL	cg16731318	C	-0.67	4.19E-08	NA	Esophagus
rs3827715	mQTL	cg15925478	NA	-0.22	7.78E-08	0.0245	Blood
rs3827715	mQTL	cg16731318	C	-0.72	8.25E-08	NA	Uterus
rs3827715	mQTL	cg08237401	NA	0.26	9.86E-08	0.0304	Blood
rs3827715	mQTL	cg16731318	C	-0.66	3.33E-07	NA	Blood
rs3827715	mQTL	cg15925478	NA	-0.55	1.69E-06	1.94E-05	Blood-Monocytes CD14+
rs3827715	mQTL	cg22807700	NA	NA	5.25E-06	0.006	Brain-Hippocampus
rs3827715	mQTL	cg08237401	NA	0.51	1.03E-05	0.000105	Blood-Monocytes CD14+
rs3827715	mQTL	cg13856647	NA	0.50	1.93E-05	0.00209	Blood-Monocytes CD14+
rs3827715	mQTL	cg08237401	C	0.10	2.38E-05	NA	Blood
rs3827715	mQTL	cg26688893	C	0.05	0.000202	NA	Blood
rs3827715	mQTL	cg22807700	NA	NA	0.000237	NA	Brain-Temporal Cortex
rs3827715	mQTL	cg13856647	C	0.06	0.000308	NA	Blood
rs3827715	mQTL	cg22807700	NA	NA	0.00035	NA	Brain-Pons
rs3827715	mQTL	cg22807700	NA	-0.40	0.000703	0.02	Blood-Monocytes CD14+
rs3827715	mQTL	cg26829310	NA	-0.39	0.000835	0.0461	Blood-Monocytes CD14+
rs3827715	mQTL	cg16731318	C	-0.61	1.12E-27	NA	Breast
rs7517826	mQTL	cg22807700	NA	-0.07	2.4E-15	NA	Brain-Cerebellum
rs7517826	mQTL	cg22807700	A	-0.02	5.15E-15	3.65E-13	Brain-Prefrontal Cortex
rs7517826	mQTL	cg22807700	A	-0.10	5.5E-15	NA	Blood
rs7517826	mQTL	cg22807700	NA	NA	1.15E-14	NA	Brain-Cerebellum
rs7517826	mQTL	cg26829310	NA	-0.27	1.41E-11	7.48E-06	Blood
rs7517826	mQTL	cg26688893	NA	0.24	7.14E-10	0.000316	Blood
rs7517826	mQTL	cg26688893	A	0.06	7.3E-10	NA	Blood
rs7517826	mQTL	cg13856647	A	0.41	8.56E-10	NA	Bladder
rs7517826	mQTL	cg08237401	NA	0.29	3.49E-09	0.00159	Blood
rs7517826	mQTL	cg22807700	NA	-0.26	4.29E-09	0.00159	Blood
rs7517826	mQTL	cg26688893	NA	0.24	8.25E-09	0.00404	Blood
rs7517826	mQTL	cg26829310	NA	-0.27	1.12E-08	0.00455	Blood
rs7517826	mQTL	cg22807700	NA	-0.30	1.2E-08	0.00574	Blood
rs7517826	mQTL	cg26829310	NA	-0.25	1.64E-08	0.00694	Blood
rs7517826	mQTL	cg26829310	A	-0.09	4.97E-08	NA	Blood
rs7517826	mQTL	cg26829310	NA	-0.22	6.2E-08	0.0201	Blood
rs7517826	mQTL	cg26688893	NA	0.21	9.03E-08	0.0335	Blood
rs7517826	mQTL	cg22807700	NA	NA	4E-07	NA	Brain-Temporal Cortex

rs7517826	mQTL	cg16731318	A	-0.67	7.46E-07	NA	Thymus
rs7517826	mQTL	cg22807700	NA	-0.51	8.57E-07	0.00022	Blood-Monocytes CD14+
rs7517826	mQTL	cg22807700	NA	NA	2.84E-06	NA	Brain-Frontal Cortex
rs7517826	mQTL	cg08237401	A	0.08	5.36E-06	NA	Blood
rs7517826	mQTL	cg22807700	NA	NA	8.15E-05	NA	Brain-Pons
rs7517826	mQTL	cg21201500	A	-0.05	0.000103	NA	Blood
rs7517826	mQTL	cg15925478	NA	-0.41	0.000111	0.000744	Blood-Monocytes CD14+
rs7517826	mQTL	cg08237401	NA	0.40	0.000127	0.000821	Blood-Monocytes CD14+
rs7517826	mQTL	cg26829310	NA	-0.38	0.000336	0.0431	Blood-Monocytes CD14+
rs7517826	mQTL	cg13502903	A	-0.06	0.000347	NA	Blood

mQTL - methylation quantitative trait locus, sQTL - splicing quantitative trait locus.
NA, not available
