# Supplementary material for: The Impact of Genetic Polymorphisms in Glutamate-Cysteine Ligase, a Key Enzyme of Glutathione Biosynthesis, on Ischemic Stroke Risk and Brain Infarct Size
Source: Life (Basel). 2022 Apr 18;12(4):602. doi: 10.3390/life12040602 (PMC9032935; doi:10.3390/life12040602)
Supplement: Supplementary file 1 [file life-12-00602-s001.zip › Supplementary table S7.pdf]

Supplementary table S7

Data on epigenetic regulatory potential of *GCLM* and *GCLC* gene polymorphisms

rs12524494

ENCODE

SNP	Chromosome	Region Start	Region End	Feature Type Class	Feature Type	Epigenome
rs12524494	chr6	53495960	53507640	Histone	H3K36me3	Monocytes-CD14+
rs12524494	chr6	53502520	53507600	Histone	H3K36me3	HMEC
rs12524494	chr6	53502860	53507640	Histone	H3K36me3	DND-41
rs12524494	chr6	53503800	53507640	Histone	H3K36me3	NHEK
rs12524494	chr6	53505300	53507660	Histone	H3K36me3	HepG2
rs12524494	chr6	53505400	53507580	Histone	H3K36me3	A549
rs12524494	chr6	53506200	53507460	Histone	H3K36me3	NH-A*

Roadmap Epigenomics

SNP	Chromosome	Region Start	Region End	Feature Type Class	Feature Type	Epigenome
rs12524494	chr6	53495880	53511800	Histone	H3K36me3	Monocytes-CD14+ (PB) Roadmap
rs12524494	chr6	53495880	53511800	Histone	H3K36me3	Monocytes-CD14+ (PB) Roadmap
rs12524494	chr6	53496460	53507660	Histone	H3K36me3	iPS-20b
rs12524494	chr6	53497640	53509680	Histone	H3K36me3	Natural Killer cells (PB)
rs12524494	chr6	53497640	53509680	Histone	H3K36me3	Natural Killer cells (PB)
rs12524494	chr6	53502080	53507460	Histone	H3K36me3	B cells (PB) Roadmap
rs12524494	chr6	53502080	53507460	Histone	H3K36me3	B cells (PB) Roadmap
rs12524494	chr6	53502860	53507700	Histone	H3K36me3	T cells (PB) Roadmap
rs12524494	chr6	53502860	53507700	Histone	H3K36me3	T cells (PB) Roadmap
rs12524494	chr6	53503720	53507640	Histone	H3K36me3	Fetal Adrenal Gland
rs12524494	chr6	53503720	53507640	Histone	H3K36me3	Fetal Adrenal Gland
rs12524494	chr6	53503760	53507660	Histone	H3K36me3	Fetal Thymus
rs12524494	chr6	53503760	53507660	Histone	H3K36me3	Fetal Thymus
rs12524494	chr6	53503800	53507580	Histone	H3K36me3	Placenta
rs12524494	chr6	53503800	53507580	Histone	H3K36me3	Placenta
rs12524494	chr6	53506640	53507560	Histone	H3K36me3	Fetal Muscle Leg
rs12524494	chr6	53506640	53507560	Histone	H3K36me3	Fetal Muscle Leg
rs12524494	chr6	53506660	53507640	Histone	H3K36me3	Fetal Intestine Large
rs12524494	chr6	53506660	53507640	Histone	H3K36me3	Fetal Intestine Large

rs17883901

ENCODE

SNP	Chromosome	Region Start	Region End	Feature Type Class	Feature Type	Epigenome
rs17883901	chr6	53539900	53545880	Histone	H3K4me1	Monocytes-CD14+
rs17883901	chr6	53540344	53545981	Histone	H3K4me3	A549
rs17883901	chr6	53541755	53545672	Histone	H3K4me3	HMEC
rs17883901	chr6	53542340	53546360	Histone	H3K4me1	K562
rs17883901	chr6	53542872	53545995	Histone	H3K4me2	DND-41
rs17883901	chr6	53542881	53545907	Histone	H3K4me2	HMEC
rs17883901	chr6	53542884	53545328	Polymerase	PolII	NHEK
rs17883901	chr6	53542887	53545770	Histone	H3K4me3	HepG2
rs17883901	chr6	53542888	53545831	Histone	H3K4me2	HepG2
rs17883901	chr6	53542892	53545800	Histone	H3K4me3	NHEK
rs17883901	chr6	53542893	53545947	Histone	H3K4me3	DND-41
rs17883901	chr6	53542899	53545656	Histone	H3K4me3	GM12878
rs17883901	chr6	53542968	53545614	Histone	H3K9ac	HepG2
rs17883901	chr6	53542990	53545505	Histone	H3K4me3	HeLa-S3
rs17883901	chr6	53543037	53545515	Histone	H3K4me3	NHLF
rs17883901	chr6	53543044	53545836	Histone	H3K27ac	HepG2
rs17883901	chr6	53543044	53545524	Histone	H3K9ac	NHEK
rs17883901	chr6	53543045	53545649	Histone	H3K4me2	Osteobl
rs17883901	chr6	53543060	53545756	Histone	H3K4me2	NHDF-AD
rs17883901	chr6	53543091	53545740	Histone	H3K4me2	NH-A

rs17883901	chr6	53543188	53545637	Histone	H3K27ac	NHEK	
rs17883901	chr6	53543259	53545690	Histone	H3K4me3	NHDF-AD	
rs17883901	chr6	53543313	53545858	Histone	H3K4me3	Monocytes-CD14+	
rs17883901	chr6	53543362	53545813	Histone	H3K9ac	DND-41	
rs17883901	chr6	53543364	53545567	Histone	H3K4me3	K562	
rs17883901	chr6	53543392	53545569	Histone	H3K4me3	H1ESC	
rs17883901	chr6	53543426	53545562	Histone	H2AZ	GM12878	
rs17883901	chr6	53543448	53545499	Histone	H3K4me3	HUVEC	
rs17883901	chr6	53543486	53545794	Histone	H3K4me2	Monocytes-CD14+	
rs17883901	chr6	53543534	53545604	Histone	H3K4me3	Osteobl	
rs17883901	chr6	53543535	53545806	Histone	H2AZ	K562	
rs17883901	chr6	53543537	53545638	Histone	H3K9ac	K562	
rs17883901	chr6	53543551	53545417	Transcription Factor	HEY1	HepG2	
rs17883901	chr6	53543643	53545434	Histone	H3K4me3	NH-A	
rs17883901	chr6	53543710	53545583	Histone	H2AZ	HSMM	
rs17883901	chr6	53543783	53545567	Histone	H3K4me3	HSMM	
rs17883901	chr6	53543953	53545511	Transcription Factor		Sin3Ak20	HepG2
rs17883901	chr6	53544032	53545336	Transcription Factor		Sin3Ak20	H1ESC
rs17883901	chr6	53544067	53545400	Polymerase	PolII	H1ESC	
rs17883901	chr6	53544079	53545311	Transcription Factor		Rad21	HepG2
rs17883901	chr6	53544085	53545525	Histone	H2AZ	HSMMtube	
rs17883901	chr6	53544118	53545400	Transcription Factor		Ini1	HeLa-S3
rs17883901	chr6	53544130	53545325	Polymerase	PolII	HeLa-S3	
rs17883901	chr6	53544142	53545338	Transcription Factor		Jund	H1ESC
rs17883901	chr6	53544165	53545391	Transcription Factor		CTCF	HepG2
rs17883901	chr6	53544195	53545339	Transcription Factor		ZBTB7A	K562
rs17883901	chr6	53544199	53545349	Transcription Factor		ETS1	K562
rs17883901	chr6	53544206	53545429	Open Chromatin	DNase1	HepG2	
rs17883901	chr6	53544306	53545398	Transcription Factor		TAF1	HepG2
rs17883901	chr6	53544314	53545431	Open Chromatin	DNase1	H1ESC	
rs17883901	chr6	53544325	53545354	Transcription Factor		ELF1	GM12878
rs17883901	chr6	53544347	53545297	Transcription Factor		Yy1	HepG2
rs17883901	chr6	53544361	53545260	Transcription Factor		TAF1	HeLa-S3
rs17883901	chr6	53544368	53545511	Transcription Factor		Pax5	GM12878
rs17883901	chr6	53544371	53545383	Open Chromatin	DNase1	HUVEC	
rs17883901	chr6	53544382	53545323	Polymerase	PolII	K562	
rs17883901	chr6	53544393	53545270	Transcription Factor		Yy1	GM12891
rs17883901	chr6	53544399	53545268	Transcription Factor		Yy1	H1ESC
rs17883901	chr6	53544413	53545267	Transcription Factor		ETS1	GM12878
rs17883901	chr6	53544428	53545337	Open Chromatin	DNase1	Monocytes-CD14+	
rs17883901	chr6	53544429	53545355	Polymerase	PolII	GM12878	
rs17883901	chr6	53544437	53545370	Transcription Factor		USF1	SKNSHRA
rs17883901	chr6	53544462	53545327	Open Chromatin	DNase1	NHDF-AD	
rs17883901	chr6	53544470	53545242	Transcription Factor		Rad21	H1ESC
rs17883901	chr6	53544471	53545372	Open Chromatin	DNase1	GM12878	
rs17883901	chr6	53544480	53545271	Transcription Factor		Sin3Ak20	K562
rs17883901	chr6	53544487	53545338	Transcription Factor		TAF7	H1ESC
rs17883901	chr6	53544493	53545403	Open Chromatin	DNase1	HSMMtube	
rs17883901	chr6	53544497	53545440	Transcription Factor		Yy1	SKNSHRA
rs17883901	chr6	53544507	53545256	Transcription Factor		p300	HepG2
rs17883901	chr6	53544512	53545336	Transcription Factor		Yy1	HCT116
rs17883901	chr6	53544515	53545366	Open Chromatin	DNase1	NH-A	
rs17883901	chr6	53544519	53545409	Open Chromatin	DNase1	K562	
rs17883901	chr6	53544531	53545394	Open Chromatin	DNase1	NHLF	
rs17883901	chr6	53544555	53545288	Transcription Factor		Rad21	SKNSHRA
rs17883901	chr6	53544579	53545291	Transcription Factor		Pax5	GM12892
rs17883901	chr6	53544611	53545367	Transcription Factor		ELF1	HepG2
rs17883901	chr6	53544617	53545339	Transcription Factor		SP1	H1ESC
rs17883901	chr6	53544622	53545267	Transcription Factor		Pax5	GM12891
rs17883901	chr6	53544630	53545380	Transcription Factor		Nrsf	Panc1
rs17883901	chr6	53544702	53545353	Transcription Factor		SP1	GM12878
rs17883901	chr6	53544761	53545797	Histone	H3K4me2	H1ESC	
rs17883901	chr6	53544804	53545282	Transcription Factor		Srf	K562

rs17883901	chr6	53544850	53545381	Transcription Factor	ZBTB33	HCT116
rs17883901	chr6	53544859	53545488	Open Chromatin	DNase1	HSMM
rs17883901	chr6	53544867	53545260	Transcription Factor	ELF1	K562
rs17883901	chr6	53544921	53545351	Open Chromatin	DNase1	A549
rs17883901	chr6	53544922	53545420	Transcription Factor	Jund	HepG2
rs17883901	chr6	53544933	53545357	Transcription Factor	HNF4G	HepG2
rs17883901	chr6	53544957	53545385	Transcription Factor	SP1	K562
rs17883901	chr6	53544970	53545513	Transcription Factor	Egr1	K562
rs17883901	chr6	53544972	53545771	Histone	H3K4me2	NHEK
rs17883901	chr6	53544972	53545379	Transcription Factor	Egr1	GM12878
rs17883901	chr6	53545040	53548040	Histone	H3K4me1	NHEK
rs17883901	chr6	53545060	53548020	Histone	H3K4me1	HMEC
rs17883901	chr6	53545060	53548040	Histone	H3K4me1	HepG2
rs17883901	chr6	53545123	53548003	Histone	H3K4me2	A549
rs17883901	chr6	53545137	53545611	Histone	H3K4me2	H1ESC
rs17883901	chr6	53545140	53545680	Histone	H3K4me1	HUVEC
rs17883901	chr6	53545140	53549040	Histone	H3K4me1	DND-41
rs17883901	chr6	53545142	53545629	Histone	H3K4me2	K562
rs17883901	chr6	53545191	53545570	Histone	H3K27ac	DND-41

Roadmap Epigenomics

SNP	Chromosome	Region Start	Region End	Feature Type Class	Feature Type	Epigenome
rs17883901	chr6	53541860	53546340	Histone	H3K4me1	T cells (PB) Roadmap
rs17883901	chr6	53541860	53546340	Histone	H3K4me1	T cells (PB) Roadmap
rs17883901	chr6	53542488	53545611	Histone	H3K4me3	IMR90
rs17883901	chr6	53542925	53545680	Histone	H3K4me3	iPS-20b
rs17883901	chr6	53543361	53545572	Histone	H3K4me3	Fetal Stomach
rs17883901	chr6	53543361	53545572	Histone	H3K4me3	Fetal Stomach
rs17883901	chr6	53543458	53545574	Histone	H3K4me3	Fetal Intestine Large
rs17883901	chr6	53543458	53545574	Histone	H3K4me3	Fetal Intestine Large
rs17883901	chr6	53543471	53545602	Histone	H3K27ac	IMR90
rs17883901	chr6	53543491	53545615	Histone	H3K4me3	Right Atrium
rs17883901	chr6	53543491	53545615	Histone	H3K4me3	Right Atrium
rs17883901	chr6	53543542	53545564	Histone	H3K4me3	Fetal Adrenal Gland
rs17883901	chr6	53543542	53545564	Histone	H3K4me3	Fetal Adrenal Gland
rs17883901	chr6	53543548	53545434	Histone	H3K4me3	Ovary
rs17883901	chr6	53543548	53545434	Histone	H3K4me3	Ovary
rs17883901	chr6	53543559	53545547	Histone	H3K4me3	Thymus
rs17883901	chr6	53543559	53545547	Histone	H3K4me3	Thymus
rs17883901	chr6	53543571	53545575	Histone	H3K4me3	Psoas Muscle
rs17883901	chr6	53543654	53545521	Histone	H3K4me3	Fetal Thymus
rs17883901	chr6	53543654	53545521	Histone	H3K4me3	Fetal Thymus
rs17883901	chr6	53543697	53545399	Histone	H3K4me3	iPS DF 19.11
rs17883901	chr6	53543697	53545399	Histone	H3K4me3	iPS DF 19.11
rs17883901	chr6	53543852	53545477	Histone	H3K4me3	Fetal Muscle Leg
rs17883901	chr6	53543852	53545477	Histone	H3K4me3	Fetal Muscle Leg
rs17883901	chr6	53543880	53546540	Histone	H3K27me3	Fetal Thymus
rs17883901	chr6	53543880	53546540	Histone	H3K27me3	Fetal Thymus
rs17883901	chr6	53543885	53545415	Histone	H3K4me3	Pancreas
rs17883901	chr6	53543885	53545415	Histone	H3K4me3	Pancreas
rs17883901	chr6	53543982	53545517	Histone	H3K4me3	B cells (PB) Roadmap
rs17883901	chr6	53543982	53545517	Histone	H3K4me3	B cells (PB) Roadmap
rs17883901	chr6	53544077	53545393	Histone	H3K4me3	H1-mesenchymal
rs17883901	chr6	53544077	53545393	Histone	H3K4me3	H1-mesenchymal
rs17883901	chr6	53544534	53545401	Open Chromatin	DNase1	IMR90
rs17883901	chr6	53544700	53545560	Histone	H3K4me1	Fetal Muscle Trunk
rs17883901	chr6	53544700	53545560	Histone	H3K4me1	Fetal Muscle Trunk
rs17883901	chr6	53544759	53545607	Histone	H3K4me3	Fetal Intestine Small
rs17883901	chr6	53544759	53545607	Histone	H3K4me3	Fetal Intestine Small
rs17883901	chr6	53544760	53547000	Histone	H2AK5ac	IMR90
rs17883901	chr6	53544764	53545618	Histone	H3K4me2	IMR90
rs17883901	chr6	53544766	53545537	Histone	H3K4me3	Natural Killer cells (PB)
rs17883901	chr6	53544766	53545537	Histone	H3K4me3	Natural Killer cells (PB)

rs17883901	chr6	53544772	53545528	Histone H3K4me3	Monocytes-CD14+ (PB) Roadmap
rs17883901	chr6	53544772	53545528	Histone H3K4me3	Monocytes-CD14+ (PB) Roadmap
rs17883901	chr6	53544792	53545502	Histone H3K4me3	T cells (PB) Roadmap
rs17883901	chr6	53544792	53545502	Histone H3K4me3	T cells (PB) Roadmap
rs17883901	chr6	53544909	53545559	Histone H3K4me3	Placenta
rs17883901	chr6	53544909	53545559	Histone H3K4me3	Placenta
rs17883901	chr6	53544960	53545740	Histone H3K4me1	Natural Killer cells (PB)
rs17883901	chr6	53544960	53545740	Histone H3K4me1	Natural Killer cells (PB)
rs17883901	chr6	53544960	53546200	Histone H3K4me1	Monocytes-CD14+ (PB) Roadmap
rs17883901	chr6	53544960	53546200	Histone H3K4me1	Monocytes-CD14+ (PB) Roadmap
rs17883901	chr6	53544960	53545960	Histone H3K4me1	Fetal Muscle Leg
rs17883901	chr6	53544960	53545960	Histone H3K4me1	Fetal Muscle Leg
rs17883901	chr6	53544960	53545320	Histone H3K9me3	Lung
rs17883901	chr6	53544960	53545320	Histone H3K9me3	Lung
rs17883901	chr6	53544980	53545580	Histone H3K4me1	Placenta
rs17883901	chr6	53544980	53545580	Histone H3K4me1	Placenta
rs17883901	chr6	53545000	53546000	Histone H3K4me1	B cells (PB) Roadmap
rs17883901	chr6	53545000	53546000	Histone H3K4me1	B cells (PB) Roadmap
rs17883901	chr6	53545000	53546780	Histone H3K4me1	Fetal Thymus
rs17883901	chr6	53545000	53546780	Histone H3K4me1	Fetal Thymus
rs17883901	chr6	53545020	53545360	Histone H3K4me1	H1-mesenchymal
rs17883901	chr6	53545020	53545360	Histone H3K4me1	H1-mesenchymal
rs17883901	chr6	53545100	53545820	Histone H3K4me1	Fetal Stomach
rs17883901	chr6	53545100	53545820	Histone H3K4me1	Fetal Stomach
rs17883901	chr6	53545120	53546480	Histone H2BK12ac	IMR90
rs17883901	chr6	53545160	53545320	Histone H3K4me1	H1-trophoblast
rs17883901	chr6	53545160	53545320	Histone H3K4me1	H1-trophoblast
rs17883901	chr6	53545160	53545640	Histone H3K14ac	IMR90
rs17883901	chr6	53545180	53546140	Histone H3K4me1	Fetal Intestine Small
rs17883901	chr6	53545180	53546140	Histone H3K4me1	Fetal Intestine Small
rs17883901	chr6	53545193	53545544	Histone H3K4me3	Aorta
rs17883901	chr6	53545193	53545544	Histone H3K4me3	Aorta
rs17883901	chr6	53545203	53546422	Histone H3K18ac	IMR90

Regulatory Build (Ensembl)

SNP	Chromosome	Region Start	Region End	Feature Type Class	Epigenome	Activity
rs17883901	chr6	53543400	53545601	Promoter	A549	POISED
rs17883901	chr6	53543400	53545601	Promoter	Aorta	ACTIVE
rs17883901	chr6	53543400	53545601	Promoter	Aorta	ACTIVE
rs17883901	chr6	53543400	53545601	Promoter	B cells (PB) Roadmap	ACTIVE
rs17883901	chr6	53543400	53545601	Promoter	B cells (PB) Roadmap	ACTIVE
rs17883901	chr6	53543400	53545601	Promoter	CD14+CD16- monocyte (CB)	ACTIVE
rs17883901	chr6	53543400	53545601	Promoter	CD14+CD16- monocyte (VB)	ACTIVE
rs17883901	chr6	53543400	53545601	Promoter	CD4+ ab T cell (VB)	ACTIVE
rs17883901	chr6	53543400	53545601	Promoter	CD8+ ab T cell (CB)	ACTIVE
rs17883901	chr6	53543400	53545601	Promoter	CM CD4+ ab T cell (VB)	ACTIVE
rs17883901	chr6	53543400	53545601	Promoter	DND-41	ACTIVE
rs17883901	chr6	53543400	53545601	Promoter	eosinophil (VB)	ACTIVE
rs17883901	chr6	53543400	53545601	Promoter	EPC (VB)	ACTIVE
rs17883901	chr6	53543400	53545601	Promoter	erythroblast (CB)	ACTIVE
rs17883901	chr6	53543400	53545601	Promoter	Fetal Adrenal Gland	ACTIVE
rs17883901	chr6	53543400	53545601	Promoter	Fetal Adrenal Gland	ACTIVE
rs17883901	chr6	53543400	53545601	Promoter	Fetal Intestine Large	ACTIVE
rs17883901	chr6	53543400	53545601	Promoter	Fetal Intestine Large	ACTIVE
rs17883901	chr6	53543400	53545601	Promoter	Fetal Intestine Small	ACTIVE
rs17883901	chr6	53543400	53545601	Promoter	Fetal Intestine Small	ACTIVE
rs17883901	chr6	53543400	53545601	Promoter	Fetal Muscle Leg	ACTIVE
rs17883901	chr6	53543400	53545601	Promoter	Fetal Muscle Leg	ACTIVE
rs17883901	chr6	53543400	53545601	Promoter	Fetal Muscle Trunk	ACTIVE
rs17883901	chr6	53543400	53545601	Promoter	Fetal Muscle Trunk	ACTIVE
rs17883901	chr6	53543400	53545601	Promoter	Fetal Stomach	ACTIVE
rs17883901	chr6	53543400	53545601	Promoter	Fetal Stomach	ACTIVE
rs17883901	chr6	53543400	53545601	Promoter	Fetal Thymus	ACTIVE

rs17883901	chr6	53543400	53545601	Promoter	Fetal Thymus	ACTIVE
rs17883901	chr6	53543400	53545601	Promoter	Gastric	ACTIVE
rs17883901	chr6	53543400	53545601	Promoter	Gastric	ACTIVE
rs17883901	chr6	53543400	53545601	Promoter	GM12878	ACTIVE
rs17883901	chr6	53543400	53545601	Promoter	H1-mesenchymal	ACTIVE
rs17883901	chr6	53543400	53545601	Promoter	H1-mesenchymal	ACTIVE
rs17883901	chr6	53543400	53545601	Promoter	H1-neuronal progenitor	POISED
rs17883901	chr6	53543400	53545601	Promoter	H1-neuronal progenitor	POISED
rs17883901	chr6	53543400	53545601	Promoter	H1-trophoblast	ACTIVE
rs17883901	chr6	53543400	53545601	Promoter	H1-trophoblast	ACTIVE
rs17883901	chr6	53543400	53545601	Promoter	H1ESC	POISED
rs17883901	chr6	53543400	53545601	Promoter	H9	POISED
rs17883901	chr6	53543400	53545601	Promoter	H9	POISED
rs17883901	chr6	53543400	53545601	Promoter	HeLa-S3	ACTIVE
rs17883901	chr6	53543400	53545601	Promoter	HepG2	ACTIVE
rs17883901	chr6	53543400	53545601	Promoter	HMEC	ACTIVE
rs17883901	chr6	53543400	53545601	Promoter	HSMM	ACTIVE
rs17883901	chr6	53543400	53545601	Promoter	HSMMtube	ACTIVE
rs17883901	chr6	53543400	53545601	Promoter	HUVEC	ACTIVE
rs17883901	chr6	53543400	53545601	Promoter	HUVEC prol (CB)	ACTIVE
rs17883901	chr6	53543400	53545601	Promoter	IMR90	ACTIVE
rs17883901	chr6	53543400	53545601	Promoter	iPS-20b	POISED
rs17883901	chr6	53543400	53545601	Promoter	iPS DF 19.11	ACTIVE
rs17883901	chr6	53543400	53545601	Promoter	iPS DF 19.11	ACTIVE
rs17883901	chr6	53543400	53545601	Promoter	iPS DF 6.9	ACTIVE
rs17883901	chr6	53543400	53545601	Promoter	iPS DF 6.9	ACTIVE
rs17883901	chr6	53543400	53545601	Promoter	K562	ACTIVE
rs17883901	chr6	53543400	53545601	Promoter	Left Ventricle	ACTIVE
rs17883901	chr6	53543400	53545601	Promoter	Left Ventricle	ACTIVE
rs17883901	chr6	53543400	53545601	Promoter	Lung	ACTIVE
rs17883901	chr6	53543400	53545601	Promoter	Lung	ACTIVE
rs17883901	chr6	53543400	53545601	Promoter	M0 macrophage (CB)	ACTIVE
rs17883901	chr6	53543400	53545601	Promoter	M0 macrophage (VB)	ACTIVE
rs17883901	chr6	53543400	53545601	Promoter	M1 macrophage (CB)	ACTIVE
rs17883901	chr6	53543400	53545601	Promoter	M1 macrophage (VB)	ACTIVE
rs17883901	chr6	53543400	53545601	Promoter	M2 macrophage (CB)	ACTIVE
rs17883901	chr6	53543400	53545601	Promoter	M2 macrophage (VB)	ACTIVE
rs17883901	chr6	53543400	53545601	Promoter	Monocytes-CD14+	POISED
rs17883901	chr6	53543400	53545601	Promoter	Monocytes-CD14+ (PB) Roadmap	ACTIVE
rs17883901	chr6	53543400	53545601	Promoter	Monocytes-CD14+ (PB) Roadmap	ACTIVE
rs17883901	chr6	53543400	53545601	Promoter	MSC (VB)	ACTIVE
rs17883901	chr6	53543400	53545601	Promoter	naive B cell (VB)	ACTIVE
rs17883901	chr6	53543400	53545601	Promoter	Natural Killer cells (PB)	ACTIVE
rs17883901	chr6	53543400	53545601	Promoter	Natural Killer cells (PB)	ACTIVE
rs17883901	chr6	53543400	53545601	Promoter	neutrophil (CB)	POISED
rs17883901	chr6	53543400	53545601	Promoter	neutrophil myelocyte (BM)	ACTIVE
rs17883901	chr6	53543400	53545601	Promoter	neutrophil (VB)	POISED
rs17883901	chr6	53543400	53545601	Promoter	NH-A	POISED
rs17883901	chr6	53543400	53545601	Promoter	NHDF-AD	ACTIVE
rs17883901	chr6	53543400	53545601	Promoter	NHEK	ACTIVE
rs17883901	chr6	53543400	53545601	Promoter	NHLF	ACTIVE
rs17883901	chr6	53543400	53545601	Promoter	Osteobl	POISED
rs17883901	chr6	53543400	53545601	Promoter	Ovary	ACTIVE
rs17883901	chr6	53543400	53545601	Promoter	Ovary	ACTIVE
rs17883901	chr6	53543400	53545601	Promoter	Pancreas	ACTIVE
rs17883901	chr6	53543400	53545601	Promoter	Pancreas	ACTIVE
rs17883901	chr6	53543400	53545601	Promoter	Placenta	ACTIVE
rs17883901	chr6	53543400	53545601	Promoter	Placenta	ACTIVE
rs17883901	chr6	53543400	53545601	Promoter	Psoas Muscle	ACTIVE
rs17883901	chr6	53543400	53545601	Promoter	Right Atrium	ACTIVE
rs17883901	chr6	53543400	53545601	Promoter	Right Atrium	ACTIVE
rs17883901	chr6	53543400	53545601	Promoter	Small Intestine	ACTIVE
rs17883901	chr6	53543400	53545601	Promoter	Small Intestine	ACTIVE

rs17883901	chr6	53543400	53545601	Promoter	Spleen	ACTIVE
rs17883901	chr6	53543400	53545601	Promoter	Spleen	ACTIVE
rs17883901	chr6	53543400	53545601	Promoter	T cells (PB) Roadmap	ACTIVE
rs17883901	chr6	53543400	53545601	Promoter	T cells (PB) Roadmap	ACTIVE
rs17883901	chr6	53543400	53545601	Promoter	Thymus	ACTIVE
rs17883901	chr6	53543400	53545601	Promoter	Thymus	ACTIVE

rs606548

ENCODE

SNP	Chromosome	Region Start	Region End	Feature Type Class	Feature Type	Epigenome
rs606548	chr6	53521880	53544680	Histone H3K79me2	HepG2	
rs606548	chr6	53532340	53544820	Histone H3K79me2	GM12878	
rs606548	chr6	53536380	53544020	Histone H3K4me1	HepG2	
rs606548	chr6	53536700	53538460	Histone H3K4me1	NHEK	
rs606548	chr6	53537600	53544540	Histone H4K20me1	Monocytes-CD14+	
rs606548	chr6	53537660	53538120	Histone H3K79me2	HeLa-S3	
rs606548	chr6	53537700	53538060	Histone H4K20me1	HepG2	
rs606548	chr6	53537740	53538060	Histone H3K4me1	A549	
rs606548	chr6	53537840	53538060	Histone H3K36me3	HepG2	
rs606548	chr6	53537860	53538080	Histone H4K20me1	A549	
rs606548	chr6	53537880	53538160	Histone H3K79me2	HSMMtube	

Roadmap Epigenomics

SNP	Chromosome	Region Start	Region End	Feature Type Class	Feature Type	Epigenome
rs606548	chr6	53537720	53537980	Histone H3K4me1	iPS-20b	

rs636933

ENCODE

SNP	Chromosome	Region Start	Region End	Feature Type Class	Feature Type	Epigenome
rs636933	chr6	53511880	53517920	Histone H3K4me1	HepG2	
rs636933	chr6	53511940	53517280	Histone H3K36me3	Monocytes-CD14+	
rs636933	chr6	53511940	53517080	Histone H3K4me1	A549	
rs636933	chr6	53512040	53517960	Histone H3K36me3	DND-41	
rs636933	chr6	53512080	53514320	Histone H3K36me3	A549	
rs636933	chr6	53512400	53515700	Histone H3K36me3	HMEC	
rs636933	chr6	53512420	53514360	Histone H3K36me3	HepG2	
rs636933	chr6	53513020	53517100	Histone H3K36me3	NHEK	
rs636933	chr6	53513080	53513680	Histone H3K36me3	GM12878	
rs636933	chr6	53513120	53513400	Histone H4K20me1	A549	
rs636933	chr6	53513200	53513740	Histone H3K36me3	HUVEC	

Roadmap Epigenomics

SNP	Chromosome	Region Start	Region End	Feature Type Class	Feature Type	Epigenome
rs636933	chr6	53511900	53517980	Histone H3K36me3	Monocytes-CD14+ (PB) Roadmap	
rs636933	chr6	53511900	53517980	Histone H3K36me3	Monocytes-CD14+ (PB) Roadmap	
rs636933	chr6	53511900	53517140	Histone H3K4me1	Fetal Intestine Small	
rs636933	chr6	53511900	53517140	Histone H3K4me1	Fetal Intestine Small	
rs636933	chr6	53511939	53513891	Histone H3K27ac	Fetal Intestine Small	
rs636933	chr6	53511939	53513891	Histone H3K27ac	Fetal Intestine Small	
rs636933	chr6	53511940	53515020	Histone H3K36me3	T cells (PB) Roadmap	
rs636933	chr6	53511940	53515020	Histone H3K36me3	T cells (PB) Roadmap	
rs636933	chr6	53511940	53517700	Histone H3K4me1	Fetal Intestine Large	
rs636933	chr6	53511940	53517700	Histone H3K4me1	Fetal Intestine Large	
rs636933	chr6	53512010	53514327	Histone H3K27ac	Fetal Intestine Large	
rs636933	chr6	53512010	53514327	Histone H3K27ac	Fetal Intestine Large	
rs636933	chr6	53512020	53517100	Histone H3K36me3	iPS-20b	
rs636933	chr6	53512060	53517100	Histone H3K36me3	B cells (PB) Roadmap	

rs636933	chr6	53512060	53517100	Histone	H3K36me3	B cells (PB) Roadmap
rs636933	chr6	53512080	53515720	Histone	H3K36me3	Natural Killer cells (PB)
rs636933	chr6	53512080	53515720	Histone	H3K36me3	Natural Killer cells (PB)
rs636933	chr6	53512120	53514900	Histone	H3K36me3	Placenta
rs636933	chr6	53512120	53514900	Histone	H3K36me3	Placenta
rs636933	chr6	53512380	53517340	Histone	H3K36me3	Fetal Adrenal Gland
rs636933	chr6	53512380	53517340	Histone	H3K36me3	Fetal Adrenal Gland
rs636933	chr6	53512380	53514680	Histone	H3K36me3	Fetal Thymus
rs636933	chr6	53512380	53514680	Histone	H3K36me3	Fetal Thymus
rs636933	chr6	53513020	53515080	Histone	H3K4me1	Fetal Stomach
rs636933	chr6	53513020	53515080	Histone	H3K4me1	Fetal Stomach
rs636933	chr6	53513020	53513800	Histone	H3K36me3	Fetal Muscle Trunk
rs636933	chr6	53513020	53513800	Histone	H3K36me3	Fetal Muscle Trunk
rs636933	chr6	53513020	53513840	Histone	H2AK5ac	IMR90
rs636933	chr6	53513040	53513980	Histone	H3K36me3	Fetal Muscle Leg
rs636933	chr6	53513040	53513980	Histone	H3K36me3	Fetal Muscle Leg
rs636933	chr6	53513040	53513440	Histone	H3K4me1	H1-mesenchymal
rs636933	chr6	53513040	53513440	Histone	H3K4me1	H1-mesenchymal
rs636933	chr6	53513060	53513880	Histone	H3K36me3	Thymus
rs636933	chr6	53513060	53513880	Histone	H3K36me3	Thymus
rs636933	chr6	53513060	53513380	Histone	H3K4me1	IMR90
rs636933	chr6	53513080	53513740	Histone	H3K36me3	Spleen
rs636933	chr6	53513080	53513740	Histone	H3K36me3	Spleen
rs636933	chr6	53513080	53513700	Histone	H3K4me1	Pancreas
rs636933	chr6	53513080	53513700	Histone	H3K4me1	Pancreas
rs636933	chr6	53513080	53513780	Histone	H3K36me3	Fetal Intestine Small
rs636933	chr6	53513080	53513780	Histone	H3K36me3	Fetal Intestine Small
rs636933	chr6	53513100	53513840	Histone	H3K36me3	Fetal Stomach
rs636933	chr6	53513100	53513840	Histone	H3K36me3	Fetal Stomach
rs636933	chr6	53513100	53513400	Histone	H3K36me3	H1-mesenchymal
rs636933	chr6	53513100	53513400	Histone	H3K36me3	H1-mesenchymal
rs636933	chr6	53513140	53513400	Histone	H3K36me3	H1-trophoblast
rs636933	chr6	53513140	53513400	Histone	H3K36me3	H1-trophoblast
rs636933	chr6	53513140	53513640	Histone	H2BK12ac	IMR90
rs636933	chr6	53513160	53513420	Histone	H3K4me1	Gastric
rs636933	chr6	53513160	53513420	Histone	H3K4me1	Gastric
rs636933	chr6	53513180	53513420	Histone	H4K8ac	IMR90
rs636933	chr6	53513220	53513820	Histone	H3K36me3	Fetal Intestine Large
rs636933	chr6	53513220	53513820	Histone	H3K36me3	Fetal Intestine Large
rs636933	chr6	53513240	53513680	Histone	H3K14ac	IMR90

rs648595

ENCODE

SNP	Chromosome	Region Start	Region End	Feature Type Class	Feature Type	Epigenome
rs648595	chr6	53511880	53517920	Histone	H3K4me1	HepG2
rs648595	chr6	53511940	53517280	Histone	H3K36me3	Monocytes-CD14+
rs648595	chr6	53511940	53517080	Histone	H3K4me1	A549
rs648595	chr6	53512040	53517960	Histone	H3K36me3	DND-41
rs648595	chr6	53512080	53514320	Histone	H3K36me3	A549
rs648595	chr6	53512400	53515700	Histone	H3K36me3	HMEC
rs648595	chr6	53512420	53514360	Histone	H3K36me3	HepG2
rs648595	chr6	53513020	53517100	Histone	H3K36me3	NHEK
rs648595	chr6	53513080	53513680	Histone	H3K36me3	GM12878
rs648595	chr6	53513200	53513740	Histone	H3K36me3	HUVEC
rs648595	chr6	53513478	53513892	Transcription Factor	FOXA1	HepG2

Roadmap Epigenomics

SNP	Chromosome	Region Start	Region End	Feature Type Class	Feature Type	Epigenome
rs648595	chr6	53511900	53517980	Histone	H3K36me3	Monocytes-CD14+ (PB) Roadmap
rs648595	chr6	53511900	53517980	Histone	H3K36me3	Monocytes-CD14+ (PB) Roadmap

rs648595	chr6	53511900	53517140	Histone	H3K4me1	Fetal Intestine Small
rs648595	chr6	53511900	53517140	Histone	H3K4me1	Fetal Intestine Small
rs648595	chr6	53511939	53513891	Histone	H3K27ac	Fetal Intestine Small
rs648595	chr6	53511939	53513891	Histone	H3K27ac	Fetal Intestine Small
rs648595	chr6	53511940	53515020	Histone	H3K36me3	T cells (PB) Roadmap
rs648595	chr6	53511940	53515020	Histone	H3K36me3	T cells (PB) Roadmap
rs648595	chr6	53511940	53517700	Histone	H3K4me1	Fetal Intestine Large
rs648595	chr6	53511940	53517700	Histone	H3K4me1	Fetal Intestine Large
rs648595	chr6	53512010	53514327	Histone	H3K27ac	Fetal Intestine Large
rs648595	chr6	53512010	53514327	Histone	H3K27ac	Fetal Intestine Large
rs648595	chr6	53512020	53517100	Histone	H3K36me3	iPS-20b
rs648595	chr6	53512060	53517100	Histone	H3K36me3	B cells (PB) Roadmap
rs648595	chr6	53512060	53517100	Histone	H3K36me3	B cells (PB) Roadmap
rs648595	chr6	53512080	53515720	Histone	H3K36me3	Natural Killer cells (PB)
rs648595	chr6	53512080	53515720	Histone	H3K36me3	Natural Killer cells (PB)
rs648595	chr6	53512120	53514900	Histone	H3K36me3	Placenta
rs648595	chr6	53512120	53514900	Histone	H3K36me3	Placenta
rs648595	chr6	53512380	53517340	Histone	H3K36me3	Fetal Adrenal Gland
rs648595	chr6	53512380	53517340	Histone	H3K36me3	Fetal Adrenal Gland
rs648595	chr6	53512380	53514680	Histone	H3K36me3	Fetal Thymus
rs648595	chr6	53512380	53514680	Histone	H3K36me3	Fetal Thymus
rs648595	chr6	53513020	53515080	Histone	H3K4me1	Fetal Stomach
rs648595	chr6	53513020	53515080	Histone	H3K4me1	Fetal Stomach
rs648595	chr6	53513020	53513800	Histone	H3K36me3	Fetal Muscle Trunk
rs648595	chr6	53513020	53513800	Histone	H3K36me3	Fetal Muscle Trunk
rs648595	chr6	53513020	53513840	Histone	H2AK5ac	IMR90
rs648595	chr6	53513040	53513980	Histone	H3K36me3	Fetal Muscle Leg
rs648595	chr6	53513040	53513980	Histone	H3K36me3	Fetal Muscle Leg
rs648595	chr6	53513060	53513880	Histone	H3K36me3	Thymus
rs648595	chr6	53513060	53513880	Histone	H3K36me3	Thymus
rs648595	chr6	53513080	53513740	Histone	H3K36me3	Spleen
rs648595	chr6	53513080	53513740	Histone	H3K36me3	Spleen
rs648595	chr6	53513080	53513700	Histone	H3K4me1	Pancreas
rs648595	chr6	53513080	53513700	Histone	H3K4me1	Pancreas
rs648595	chr6	53513080	53513780	Histone	H3K36me3	Fetal Intestine Small
rs648595	chr6	53513080	53513780	Histone	H3K36me3	Fetal Intestine Small
rs648595	chr6	53513100	53513840	Histone	H3K36me3	Fetal Stomach
rs648595	chr6	53513100	53513840	Histone	H3K36me3	Fetal Stomach
rs648595	chr6	53513140	53513640	Histone	H2BK12ac	IMR90
rs648595	chr6	53513220	53513820	Histone	H3K36me3	Fetal Intestine Large
rs648595	chr6	53513220	53513820	Histone	H3K36me3	Fetal Intestine Large
rs648595	chr6	53513240	53513680	Histone	H3K14ac	IMR90
rs648595	chr6	53513480	53513700	Histone	H3K4me1	Gastric
rs648595	chr6	53513480	53513700	Histone	H3K4me1	Gastric
rs648595	chr6	53513480	53513800	Histone	H3K36me3	Left Ventricle
rs648595	chr6	53513480	53513800	Histone	H3K36me3	Left Ventricle
rs648595	chr6	53513480	53513680	Histone	H3K36me3	H1-mesenchymal
rs648595	chr6	53513480	53513680	Histone	H3K36me3	H1-mesenchymal
rs648595	chr6	53513520	53513680	Histone	H3K36me3	H1-trophoblast
rs648595	chr6	53513520	53513680	Histone	H3K36me3	H1-trophoblast

rs761142

ENCODE

SNP	Chromosome	Region Start	Region End	Feature Type Class	Feature Type	Epigenome
rs761142	chr6	53521880	53544680	Histone	H3K79me2	HepG2
rs761142	chr6	53522220	53528440	Histone	H3K4me1	NHEK
rs761142	chr6	53522440	53528560	Histone	H3K4me1	HepG2
rs761142	chr6	53523863	53528261	Histone	H3K4me2	A549
rs761142	chr6	53525520	53527840	Histone	H3K4me1	HMEC
rs761142	chr6	53525960	53528020	Histone	H3K4me1	Monocytes-CD14+
rs761142	chr6	53526720	53528480	Histone	H4K20me1	Monocytes-CD14+

rs761142	chr6	53526720	53528440	Histone	H3K4me1	A549
rs761142	chr6	53526800	53527940	Histone	H3K79me2	GM12878
rs761142	chr6	53526820	53528100	Histone	H3K79me2	HeLa-S3
rs761142	chr6	53526980	53528020	Histone	H4K20me1	DND-41
rs761142	chr6	53527000	53528040	Histone	H4K20me1	A549
rs761142	chr6	53527080	53527680	Histone	H3K36me3	NHEK
rs761142	chr6	53527100	53527340	Histone	H4K20me1	NH-A

Roadmap Epigenomics

SNP	Chromosome	Region Start	Region End	Feature Type Class	Feature Type	Epigenome
rs761142	chr6	53523140	53527420	Histone	H3K4me1	Fetal Adrenal Gland
rs761142	chr6	53523140	53527420	Histone	H3K4me1	Fetal Adrenal Gland
rs761142	chr6	53525660	53528080	Histone	H2AK5ac	IMR90
rs761142	chr6	53526080	53527940	Histone	H3K4me1	Monocytes-CD14+ (PB) Roadmap
rs761142	chr6	53526080	53527940	Histone	H3K4me1	Monocytes-CD14+ (PB) Roadmap
rs761142	chr6	53526720	53528420	Histone	H3K4me1	iPS-20b
rs761142	chr6	53526800	53527520	Histone	H3K4me1	Fetal Intestine Small
rs761142	chr6	53526800	53527520	Histone	H3K4me1	Fetal Intestine Small
rs761142	chr6	53526920	53528120	Histone	H3K79me2	IMR90
rs761142	chr6	53526940	53528040	Histone	H3K4me1	iPS DF 19.11
rs761142	chr6	53526940	53528040	Histone	H3K4me1	iPS DF 19.11
rs761142	chr6	53526940	53527340	Histone	H2BK12ac	IMR90
rs761142	chr6	53526960	53528180	Histone	H3K4me1	H9
rs761142	chr6	53526960	53528180	Histone	H3K4me1	H9
rs761142	chr6	53526960	53528080	Histone	H3K4me1	H1-neuronal progenitor
rs761142	chr6	53526960	53528080	Histone	H3K4me1	H1-neuronal progenitor
rs761142	chr6	53526960	53528060	Histone	H3K14ac	IMR90
rs761142	chr6	53526980	53527420	Histone	H4K8ac	IMR90
rs761142	chr6	53527000	53528060	Histone	H3K4me1	Right Atrium
rs761142	chr6	53527000	53528060	Histone	H3K4me1	Right Atrium
rs761142	chr6	53527000	53527360	Histone	H3K4me1	H1-mesenchymal
rs761142	chr6	53527000	53527360	Histone	H3K4me1	H1-mesenchymal
rs761142	chr6	53527000	53528020	Histone	H3K4me1	Psoas Muscle
rs761142	chr6	53527000	53527380	Histone	H3K4me1	H1-trophoblast
rs761142	chr6	53527000	53527380	Histone	H3K4me1	H1-trophoblast
rs761142	chr6	53527020	53527960	Histone	H3K4me1	Pancreas
rs761142	chr6	53527020	53527960	Histone	H3K4me1	Pancreas
rs761142	chr6	53527020	53527660	Histone	H3K4me1	Gastric
rs761142	chr6	53527020	53527660	Histone	H3K4me1	Gastric
rs761142	chr6	53527040	53527360	Histone	H3K4me1	iPS DF 6.9
rs761142	chr6	53527040	53527360	Histone	H3K4me1	iPS DF 6.9
rs761142	chr6	53527060	53527860	Histone	H3K4me1	IMR90
rs761142	chr6	53527100	53528020	Histone	H3K4me1	Left Ventricle
rs761142	chr6	53527100	53528020	Histone	H3K4me1	Left Ventricle
rs761142	chr6	53527120	53527360	Histone	H3K9me3	H9
rs761142	chr6	53527120	53527360	Histone	H3K9me3	H9

rs2301022

ENCODE

SNP	Chromosome	Region Start	Region End	Feature Type Class	Feature Type	Epigenome
rs2301022	chr1	93901500	93909200	Histone	H3K79me2	HSMMtube
rs2301022	chr1	93902480	93909540	Histone	H3K79me2	K562
rs2301022	chr1	93903040	93909080	Histone	H3K79me2	HeLa-S3
rs2301022	chr1	93903360	93909100	Histone	H3K79me2	HSMM
rs2301022	chr1	93903540	93909560	Histone	H3K79me2	GM12878
rs2301022	chr1	93903660	93908920	Histone	H3K79me2	HepG2
rs2301022	chr1	93903780	93909060	Histone	H3K4me1	DND-41
rs2301022	chr1	93903800	93908800	Histone	H3K4me1	HUVEC
rs2301022	chr1	93903800	93908900	Histone	H3K4me1	HepG2
rs2301022	chr1	93903847	93908853	Histone	H3K4me2	A549

rs2301022	chr1	93904000	93908660	Histone H3K4me1	HeLa-S3
rs2301022	chr1	93904100	93908880	Histone H3K4me1	Osteobl
rs2301022	chr1	93904160	93908900	Histone H3K4me1	NHEK
rs2301022	chr1	93904675	93909591	Histone H3K4me2	HMEC
rs2301022	chr1	93904920	93907380	Histone H3K4me1	NHDF-AD
rs2301022	chr1	93905003	93909485	Histone H3K4me3	A549
rs2301022	chr1	93905195	93909175	Histone H3K4me2	HUVEC
rs2301022	chr1	93905485	93908978	Histone H3K4me2	HeLa-S3
rs2301022	chr1	93905729	93910151	Histone H3K4me3	HMEC
rs2301022	chr1	93905729	93909488	Histone H3K9ac	A549
rs2301022	chr1	93906027	93909512	Histone H3K27ac	HMEC
rs2301022	chr1	93906136	93910658	Histone H3K4me2	DND-41
rs2301022	chr1	93906200	93908200	Histone H4K20me1	NH-A
rs2301022	chr1	93906219	93909606	Histone H3K27ac	A549
rs2301022	chr1	93906238	93910361	Histone H3K4me2	NH-A
rs2301022	chr1	93906648	93909504	Histone H3K4me2	HSMM
rs2301022	chr1	93906680	93909561	Histone H3K4me2	HepG2
rs2301022	chr1	93906700	93908340	Histone H4K20me1	Osteobl
rs2301022	chr1	93906746	93909213	Histone H3K4me2	NHEK
rs2301022	chr1	93906797	93909178	Histone H3K4me2	Osteobl
rs2301022	chr1	93906840	93907560	Histone H3K36me3	HSMM
rs2301022	chr1	93906960	93908420	Histone H3K4me1	NH-A
rs2301022	chr1	93906960	93907400	Histone H3K36me3	A549
rs2301022	chr1	93906972	93910335	Histone H3K4me3	K562
rs2301022	chr1	93907020	93907460	Histone H3K4me1	NHLF
rs2301022	chr1	93907025	93909163	Histone H3K4me2	NHDF-AD
rs2301022	chr1	93907044	93909639	Histone H3K4me3	GM12878
rs2301022	chr1	93907112	93910196	Histone H3K4me3	Osteobl
rs2301022	chr1	93907171	93910515	Histone H3K4me3	DND-41
rs2301022	chr1	93907283	93909538	Histone H3K4me2	K562

Roadmap Epigenomics

SNP	Chromosome	Region Start	Region End	Feature Type Class	Feature Type	Epigenome
rs2301022	chr1	93906769	93910261	Histone H3K4me2	IMR90	
rs2301022	chr1	93906951	93910116	Histone H3K4me3	IMR90	
rs2301022	chr1	93907020	93907400	Histone H3K14ac	IMR90	
rs2301022	chr1	93907180	93908320	Histone H3K4me1	iPS-20b	

rs3827715

ENCODE

SNP	Chromosome	Region Start	Region End	Feature Type Class	Feature Type	Epigenome
rs3827715	chr1	93901500	93909200	Histone H3K79me2	HSMMtube	
rs3827715	chr1	93902480	93909540	Histone H3K79me2	K562	
rs3827715	chr1	93903040	93909080	Histone H3K79me2	HeLa-S3	
rs3827715	chr1	93903360	93909100	Histone H3K79me2	HSMM	
rs3827715	chr1	93903400	93905820	Histone H3K4me1	Monocytes-CD14+	
rs3827715	chr1	93903400	93904700	Histone H3K36me3	DND-41	
rs3827715	chr1	93903440	93905580	Histone H3K4me1	A549	
rs3827715	chr1	93903500	93904480	Histone H3K36me3	HMEC	
rs3827715	chr1	93903540	93909560	Histone H3K79me2	GM12878	
rs3827715	chr1	93903580	93904100	Histone H3K36me3	Osteobl	
rs3827715	chr1	93903660	93908920	Histone H3K79me2	HepG2	
rs3827715	chr1	93903700	93906580	Histone H3K4me1	HMEC	
rs3827715	chr1	93903700	93904540	Histone H3K36me3	NHEK	
rs3827715	chr1	93903718	93904844	Histone H2AZ	GM12878	
rs3827715	chr1	93903760	93904900	Histone H3K36me3	A549	
rs3827715	chr1	93903780	93909060	Histone H3K4me1	DND-41	
rs3827715	chr1	93903780	93904380	Histone H4K20me1	NH-A	
rs3827715	chr1	93903800	93908800	Histone H3K4me1	HUVEC	
rs3827715	chr1	93903800	93908900	Histone H3K4me1	HepG2	

rs3827715	chr1	93903800	93904480	Histone H4K20me1	A549
rs3827715	chr1	93903847	93908853	Histone H3K4me2	A549
rs3827715	chr1	93903899	93904381	Histone H3K27ac	Monocytes-CD14+
rs3827715	chr1	93903900	93904580	Histone H3K36me3	HSMM
rs3827715	chr1	93903900	93904360	Histone H3K4me1	GM12878
rs3827715	chr1	93904000	93908660	Histone H3K4me1	HeLa-S3

Roadmap Epigenomics

SNP	Chromosome	Region Start	Region End	Feature Type Class	Feature Type	Epigenome
rs3827715	chr1	93903300	93905340	Histone	H3K4me1	Natural Killer cells (PB)
rs3827715	chr1	93903300	93905340	Histone	H3K4me1	Natural Killer cells (PB)
rs3827715	chr1	93903420	93905640	Histone	H3K4me1	Fetal Adrenal Gland
rs3827715	chr1	93903420	93905640	Histone	H3K4me1	Fetal Adrenal Gland
rs3827715	chr1	93903540	93905560	Histone	H3K4me1	B cells (PB) Roadmap
rs3827715	chr1	93903540	93905560	Histone	H3K4me1	B cells (PB) Roadmap
rs3827715	chr1	93903660	93905620	Histone	H3K4me1	Monocytes-CD14+ (PB) Roadmap
rs3827715	chr1	93903660	93905620	Histone	H3K4me1	Monocytes-CD14+ (PB) Roadmap
rs3827715	chr1	93903780	93904860	Histone	H3K36me3	iPS-20b
rs3827715	chr1	93903798	93904922	Histone	H3K27ac	T cells (PB) Roadmap
rs3827715	chr1	93903798	93904922	Histone	H3K27ac	T cells (PB) Roadmap
rs3827715	chr1	93903845	93905338	Histone	H3K27ac	Natural Killer cells (PB)
rs3827715	chr1	93903845	93905338	Histone	H3K27ac	Natural Killer cells (PB)
rs3827715	chr1	93903860	93904620	Histone	H3K14ac	IMR90

Regulatory Build (Ensembl)

SNP	Chromosome	Region Start	Region End	Feature Type Class	Epigenome	Activity
rs3827715	chr1	93904001	93905200	Promoter Flanking Region	A549	POISED
rs3827715	chr1	93904001	93905200	Promoter Flanking Region	Aorta	INACTIVE
rs3827715	chr1	93904001	93905200	Promoter Flanking Region	Aorta	INACTIVE
rs3827715	chr1	93904001	93905200	Promoter Flanking Region	B cells (PB) Roadmap	INACTIVE
rs3827715	chr1	93904001	93905200	Promoter Flanking Region	B cells (PB) Roadmap	INACTIVE
rs3827715	chr1	93904001	93905200	Promoter Flanking Region	CD14+CD16- monocyte (CB)	ACTIVE
rs3827715	chr1	93904001	93905200	Promoter Flanking Region	CD14+CD16- monocyte (VB)	INACTIVE
rs3827715	chr1	93904001	93905200	Promoter Flanking Region	CD4+ ab T cell (VB)	INACTIVE
rs3827715	chr1	93904001	93905200	Promoter Flanking Region	CD8+ ab T cell (CB)	INACTIVE
rs3827715	chr1	93904001	93905200	Promoter Flanking Region	CM CD4+ ab T cell (VB)	INACTIVE
rs3827715	chr1	93904001	93905200	Promoter Flanking Region	DND-41	ACTIVE
rs3827715	chr1	93904001	93905200	Promoter Flanking Region	eosinophil (VB)	INACTIVE
rs3827715	chr1	93904001	93905200	Promoter Flanking Region	EPC (VB)	INACTIVE
rs3827715	chr1	93904001	93905200	Promoter Flanking Region	erythroblast (CB)	INACTIVE
rs3827715	chr1	93904001	93905200	Promoter Flanking Region	Fetal Adrenal Gland	INACTIVE
rs3827715	chr1	93904001	93905200	Promoter Flanking Region	Fetal Adrenal Gland	INACTIVE
rs3827715	chr1	93904001	93905200	Promoter Flanking Region	Fetal Intestine Large	INACTIVE
rs3827715	chr1	93904001	93905200	Promoter Flanking Region	Fetal Intestine Large	INACTIVE
rs3827715	chr1	93904001	93905200	Promoter Flanking Region	Fetal Intestine Small	INACTIVE
rs3827715	chr1	93904001	93905200	Promoter Flanking Region	Fetal Intestine Small	INACTIVE
rs3827715	chr1	93904001	93905200	Promoter Flanking Region	Fetal Muscle Leg	INACTIVE
rs3827715	chr1	93904001	93905200	Promoter Flanking Region	Fetal Muscle Leg	INACTIVE
rs3827715	chr1	93904001	93905200	Promoter Flanking Region	Fetal Muscle Trunk	INACTIVE
rs3827715	chr1	93904001	93905200	Promoter Flanking Region	Fetal Muscle Trunk	INACTIVE
rs3827715	chr1	93904001	93905200	Promoter Flanking Region	Fetal Stomach	INACTIVE
rs3827715	chr1	93904001	93905200	Promoter Flanking Region	Fetal Stomach	INACTIVE
rs3827715	chr1	93904001	93905200	Promoter Flanking Region	Fetal Thymus	INACTIVE
rs3827715	chr1	93904001	93905200	Promoter Flanking Region	Fetal Thymus	INACTIVE
rs3827715	chr1	93904001	93905200	Promoter Flanking Region	Gastric	INACTIVE
rs3827715	chr1	93904001	93905200	Promoter Flanking Region	Gastric	INACTIVE
rs3827715	chr1	93904001	93905200	Promoter Flanking Region	GM12878	ACTIVE
rs3827715	chr1	93904001	93905200	Promoter Flanking Region	H1-mesenchymal	INACTIVE
rs3827715	chr1	93904001	93905200	Promoter Flanking Region	H1-mesenchymal	INACTIVE
rs3827715	chr1	93904001	93905200	Promoter Flanking Region	H1-neuronal progenitor	INACTIVE
rs3827715	chr1	93904001	93905200	Promoter Flanking Region	H1-neuronal progenitor	INACTIVE
rs3827715	chr1	93904001	93905200	Promoter Flanking Region	H1-trophoblast	INACTIVE
rs3827715	chr1	93904001	93905200	Promoter Flanking Region	H1-trophoblast	INACTIVE
rs3827715	chr1	93904001	93905200	Promoter Flanking Region	H1ESC	INACTIVE

rs3827715	chr1	93904001	93905200	Promoter Flanking Region	H9	INACTIVE
rs3827715	chr1	93904001	93905200	Promoter Flanking Region	H9	INACTIVE
rs3827715	chr1	93904001	93905200	Promoter Flanking Region	HeLa-S3	INACTIVE
rs3827715	chr1	93904001	93905200	Promoter Flanking Region	HepG2	INACTIVE
rs3827715	chr1	93904001	93905200	Promoter Flanking Region	HMEC	ACTIVE
rs3827715	chr1	93904001	93905200	Promoter Flanking Region	HSMM	INACTIVE
rs3827715	chr1	93904001	93905200	Promoter Flanking Region	HSMMtube	INACTIVE
rs3827715	chr1	93904001	93905200	Promoter Flanking Region	HUVEC	ACTIVE
rs3827715	chr1	93904001	93905200	Promoter Flanking Region	HUVEC prol (CB)	INACTIVE
rs3827715	chr1	93904001	93905200	Promoter Flanking Region	IMR90	POISED
rs3827715	chr1	93904001	93905200	Promoter Flanking Region	iPS-20b	INACTIVE
rs3827715	chr1	93904001	93905200	Promoter Flanking Region	iPS DF 19.11	INACTIVE
rs3827715	chr1	93904001	93905200	Promoter Flanking Region	iPS DF 19.11	INACTIVE
rs3827715	chr1	93904001	93905200	Promoter Flanking Region	iPS DF 6.9	INACTIVE
rs3827715	chr1	93904001	93905200	Promoter Flanking Region	iPS DF 6.9	INACTIVE
rs3827715	chr1	93904001	93905200	Promoter Flanking Region	K562	REPRESSED
rs3827715	chr1	93904001	93905200	Promoter Flanking Region	Left Ventricle	INACTIVE
rs3827715	chr1	93904001	93905200	Promoter Flanking Region	Left Ventricle	INACTIVE
rs3827715	chr1	93904001	93905200	Promoter Flanking Region	Lung	INACTIVE
rs3827715	chr1	93904001	93905200	Promoter Flanking Region	Lung	INACTIVE
rs3827715	chr1	93904001	93905200	Promoter Flanking Region	M0 macrophage (CB)	INACTIVE
rs3827715	chr1	93904001	93905200	Promoter Flanking Region	M0 macrophage (VB)	ACTIVE
rs3827715	chr1	93904001	93905200	Promoter Flanking Region	M1 macrophage (CB)	INACTIVE
rs3827715	chr1	93904001	93905200	Promoter Flanking Region	M1 macrophage (VB)	ACTIVE
rs3827715	chr1	93904001	93905200	Promoter Flanking Region	M2 macrophage (CB)	INACTIVE
rs3827715	chr1	93904001	93905200	Promoter Flanking Region	M2 macrophage (VB)	INACTIVE
rs3827715	chr1	93904001	93905200	Promoter Flanking Region	Monocytes-CD14+	ACTIVE
rs3827715	chr1	93904001	93905200	Promoter Flanking Region	Monocytes-CD14+ (PB) Roadmap	INACTIVE
rs3827715	chr1	93904001	93905200	Promoter Flanking Region	Monocytes-CD14+ (PB) Roadmap	INACTIVE
rs3827715	chr1	93904001	93905200	Promoter Flanking Region	MSC (VB)	INACTIVE
rs3827715	chr1	93904001	93905200	Promoter Flanking Region	naive B cell (VB)	ACTIVE
rs3827715	chr1	93904001	93905200	Promoter Flanking Region	Natural Killer cells (PB)	INACTIVE
rs3827715	chr1	93904001	93905200	Promoter Flanking Region	Natural Killer cells (PB)	INACTIVE
rs3827715	chr1	93904001	93905200	Promoter Flanking Region	neutrophil (CB)	INACTIVE
rs3827715	chr1	93904001	93905200	Promoter Flanking Region	neutrophil myelocyte (BM)	INACTIVE
rs3827715	chr1	93904001	93905200	Promoter Flanking Region	neutrophil (VB)	ACTIVE
rs3827715	chr1	93904001	93905200	Promoter Flanking Region	NH-A	REPRESSED
rs3827715	chr1	93904001	93905200	Promoter Flanking Region	NHDF-AD	INACTIVE
rs3827715	chr1	93904001	93905200	Promoter Flanking Region	NHEK	INACTIVE
rs3827715	chr1	93904001	93905200	Promoter Flanking Region	NHLF	INACTIVE
rs3827715	chr1	93904001	93905200	Promoter Flanking Region	Osteobl	INACTIVE
rs3827715	chr1	93904001	93905200	Promoter Flanking Region	Ovary	INACTIVE
rs3827715	chr1	93904001	93905200	Promoter Flanking Region	Ovary	INACTIVE
rs3827715	chr1	93904001	93905200	Promoter Flanking Region	Pancreas	INACTIVE
rs3827715	chr1	93904001	93905200	Promoter Flanking Region	Pancreas	INACTIVE
rs3827715	chr1	93904001	93905200	Promoter Flanking Region	Placenta	INACTIVE
rs3827715	chr1	93904001	93905200	Promoter Flanking Region	Placenta	INACTIVE
rs3827715	chr1	93904001	93905200	Promoter Flanking Region	Psoas Muscle	INACTIVE
rs3827715	chr1	93904001	93905200	Promoter Flanking Region	Right Atrium	INACTIVE
rs3827715	chr1	93904001	93905200	Promoter Flanking Region	Right Atrium	INACTIVE
rs3827715	chr1	93904001	93905200	Promoter Flanking Region	Small Intestine	INACTIVE
rs3827715	chr1	93904001	93905200	Promoter Flanking Region	Small Intestine	INACTIVE
rs3827715	chr1	93904001	93905200	Promoter Flanking Region	Spleen	INACTIVE
rs3827715	chr1	93904001	93905200	Promoter Flanking Region	Spleen	INACTIVE
rs3827715	chr1	93904001	93905200	Promoter Flanking Region	T cells (PB) Roadmap	INACTIVE
rs3827715	chr1	93904001	93905200	Promoter Flanking Region	T cells (PB) Roadmap	INACTIVE
rs3827715	chr1	93904001	93905200	Promoter Flanking Region	Thymus	INACTIVE
rs3827715	chr1	93904001	93905200	Promoter Flanking Region	Thymus	INACTIVE

rs7517826

ENCODE

SNP	Chromosome	Region Start	Region End	Feature Type Class	Feature Type	Epigenome
rs7517826	chr1	93892080	93901680	Histone	H3K36me3	Osteobl
rs7517826	chr1	93898500	93902040	Histone	H3K36me3	DND-41
rs7517826	chr1	93898500	93901700	Histone	H3K36me3	A549
rs7517826	chr1	93899200	93899900	Histone	H3K36me3	HMEC
rs7517826	chr1	93899720	93900260	Histone	H3K36me3	HepG2

Roadmap Epigenomics

SNP	Chromosome	Region Start	Region End	Feature Type Class	Feature Type	Epigenome
rs7517826	chr1	93898420	93899820	Histone	H3K36me3	Fetal Adrenal Gland
rs7517826	chr1	93898420	93899820	Histone	H3K36me3	Fetal Adrenal Gland
rs7517826	chr1	93899280	93899920	Histone	H3K36me3	iPS-20b
rs7517826	chr1	93899500	93901940	Histone	H3K36me3	Placenta
rs7517826	chr1	93899500	93901940	Histone	H3K36me3	Placenta

* NH-A normal human astrocytes

Epigenetic signatures in brain tissues or aorta are highlighted by yellow
