# Supplementary material for: The Impact of Genetic Polymorphisms in Glutamate-Cysteine Ligase, a Key Enzyme of Glutathione Biosynthesis, on Ischemic Stroke Risk and Brain Infarct Size
Source: Life (Basel). 2022 Apr 18;12(4):602. doi: 10.3390/life12040602 (PMC9032935; doi:10.3390/life12040602)
Supplement: Supplementary file 1 [file life-12-00602-s001.zip › Supplementary table S8.pdf]

Supplementary table S8

A list of transcription factors predicted by atSNP Search whose binding sites are located in the regions around SNPs of *GCLM* and *GCLC* genes

Rs ID	Alleles	TFBS	*P*-value for SNP impact on TF binding	Transcription factor/protein name	Effect on transcription
A>G rs12524494 of *GCLC*					
rs12524494	A	HOXC12	0,00688448	homeobox C12	activator
rs12524494	A	MYEF2	0,00754157	myelin expression factor 2	repressor
rs12524494	A	FOXD3	0,00973114	forkhead box D3	repressor
rs12524494	A	RREB1	0,01310995	ras responsive element binding protein 1	activator
rs12524494	A	HOXD12	0,0131632	homeobox D12	unknown
rs12524494	A	FOXD3	0,0154714	forkhead box D3	repressor
rs12524494	A	POU6F1	0,01690969	POU class 6 homeobox 1	unknown
rs12524494	A	PROP1	0,02243648	PROP paired-like homeobox 1	activator
rs12524494	A	HOXC11	0,02277948	homeobox C11	unknown
rs12524494	A	HOXD11	0,02540463	homeobox D11	unknown
rs12524494	A	HOXC11	0,02617546	homeobox C11	unknown
rs12524494	A	HOXC10	0,02658399	homeobox C10	unknown
rs12524494	A	AP1	0,02963262	adaptor related protein complex 1	activator
rs12524494	A	PROP1	0,03052568	PROP paired-like homeobox 1	activator
rs12524494	A	HOXD11	0,03462219	homeobox D11	unknown
rs12524494	A	MEF2C	0,03470955	myocyte enhancer factor 2D	activator
rs12524494	A	HOXA10	0,03523246	homeobox A10	unknown
rs12524494	A	HOXC10	0,03816001	homeobox C10	unknown
rs12524494	A	MEF2A	0,03998132	myocyte enhancer factor 2D	activator
rs12524494	A	Hoxa9	0,04083132	homeobox A9	unknown
rs12524494	A	RREB1	0,04166861	ras responsive element binding protein 1	activator
rs12524494	A	ALX4	0,04883897	ALX homeobox 4	activator

rs12524494	A	MEF2D	0,04945944	myocyte enhancer factor 2D	activator
rs12524494	A	ARX	0,04988756	aristaless related homeobox	unknown
rs12524494	G	SRY	0,00009106	sex determining region Y	activator
rs12524494	G	HSFY2	0,00122658	heat shock transcription factor Y-linked 2	activator
rs12524494	G	SPDEF	0,00207513	SAM pointed domain containing ETS transcription factor	activator
rs12524494	G	PRDM1	0,00930715	PR/SET domain 1	repressor
rs12524494	G	NFAT	0,00979334	Nuclear factor of activated T-cells	activator
rs12524494	G	IRF1	0,00981276	interferon regulatory factor 1	activator
rs12524494	G	HSFY2	0,0117527	heat shock transcription factor Y-linked 2	activator
rs12524494	G	IRF	0,0118172	tripartite motif containing 63	unknown
rs12524494	G	PRDM1	0,01586607	PR/SET domain 1	repressor
rs12524494	G	IRF8	0,02036629	interferon regulatory factor 8	dual
rs12524494	G	MEF2A	0,02089385	myocyte enhancer factor 2A	activator
rs12524494	G	Spi1	0,0254237	Spi-1 proto-oncogene	activator
rs12524494	G	STAT5A	0,02677579	signal transducer and activator of transcription 5A	activator
rs12524494	G	FOXA	0,03034514	Forkhead box protein A	activator
rs12524494	G	AR	0,03196412	androgen receptor	dual
rs12524494	G	MYC	0,03319387	MYC proto-oncogene, bHLH transcription factor	dual
rs12524494	G	SRF	0,03389341	serum response factor	activator
rs12524494	G	SPIC	0,03634069	Spi-C transcription factor	activator
rs12524494	G	REST	0,03803775	RE1 silencing transcription factor	dual
rs12524494	G	ARHGEF12	0,04307261	Rho guanine nucleotide exchange factor 12	activator
rs12524494	G	GATA	0,04564428	glutaminyl-tRNA amidotransferase subunit QRSL1	unknown
rs12524494	G	AR	0,0475197	androgen receptor	dual
G>A rs17883901 of *GCLC*					
rs17883901	G	REST	0,00460551	RE1 silencing transcription factor	dual
rs17883901	G	SIX5	0,00885429	SIX homeobox 5	activator
rs17883901	G	CTCF	0,01011891	CCCTC-binding factor	repressor
rs17883901	G	MYC	0,01122463	MYC proto-oncogene, bHLH transcription factor	dual
rs17883901	G	SMARCC1	0,01759352	SWI/SNF related, matrix associated, actin dependent regulator of chromatin subfamily c member 1	activator

rs17883901	G	CENPB	0,01925176	centromere protein B	repressor
rs17883901	G	PAX5	0,02215997	paired box 5	activator
rs17883901	G	ZIC1	0,02436389	Zic family member 1	repressor
rs17883901	G	RAD21	0,03175998	RAD21 cohesin complex component	activator
rs17883901	G	EGR4	0,03403306	early growth response 4	unknown
rs17883901	G	RAD21	0,03594907	RAD21 cohesin complex component	activator
rs17883901	G	NR1H	0,04649142	nuclear receptor subfamily 1 group H	activator
rs17883901	A	PPARA	0,00000001	peroxisome proliferator activated receptor alpha	dual
rs17883901	A	CEBPB	0,00000031	CCAAT enhancer binding protein beta	dual
rs17883901	A	ESR1	0,0000009	estrogen receptor 1	activator
rs17883901	A	AP1	0,00000122	adaptor related protein complex 1	activator
rs17883901	A	NFE2L1	0,00000453	nuclear factor, erythroid 2 like 1	activator
rs17883901	A	RARA	0,0004223	retinoic acid receptor alpha	dual
rs17883901	A	HNF4	0,00133245	hepatocyte nuclear factor 4 alpha	activator
rs17883901	A	RXRG	0,00188399	retinoid X receptor gamma	unknown
rs17883901	A	RORA	0,00352615	RAR related orphan receptor A	activator
rs17883901	A	PPARG::RXRA	0,00438426	peroxisome proliferator activated receptor gamma_retinoid X receptor alpha	activator
rs17883901	A	PPARG	0,00447461	peroxisome proliferator activated receptor gamma	activator
rs17883901	A	HNF4A	0,00470891	hepatocyte nuclear factor 4 alpha	activator
rs17883901	A	CEBPG	0,00485005	CCAAT enhancer binding protein gamma	activator
rs17883901	A	JUND	0,00597178	JunB proto-oncogene, AP-1 transcription factor subunit	activator
rs17883901	A	CEBPG	0,00668313	CCAAT enhancer binding protein gamma	activator
rs17883901	A	VDR	0,00674023	vitamin D receptor	activator
rs17883901	A	JUN	0,00743989	Jun proto-oncogene, AP-1 transcription factor subunit	activator
rs17883901	A	HLF	0,00745564	HLF transcription factor, PAR bZIP family member	activator
rs17883901	A	CREB1	0,00748847	cAMP responsive element binding protein 1	dual
rs17883901	A	CEBPB	0,00755512	CCAAT enhancer binding protein beta	dual
rs17883901	A	CEBPE	0,00768707	CCAAT enhancer binding protein epsilon	activator
rs17883901	A	AP1	0,00787946	adaptor related protein complex 1	activator
rs17883901	A	CEBPD	0,00797456	CCAAT enhancer binding protein delta	activator

rs17883901	A	FOSL2	0,00797862	FOS like 2, AP-1 transcription factor subunit	unknown
rs17883901	A	HNF4A	0,00835495	hepatocyte nuclear factor 4 alpha	activator
rs17883901	A	CREB1	0,00955513	cAMP responsive element binding protein 1	dual
rs17883901	A	DBP	0,00993451	D-box binding PAR bZIP transcription factor	unknown
rs17883901	A	CEBPB	0,01011265	CCAAT enhancer binding protein beta	dual
rs17883901	A	HLF	0,01033	HLF transcription factor, PAR bZIP family member	activator
rs17883901	A	RXRA	0,01071759	retinoid X receptor alpha	activator
rs17883901	A	CREB1	0,01071957	cAMP responsive element binding protein 1	dual
rs17883901	A	HNF4A	0,01138496	hepatocyte nuclear factor 4 alpha	activator
rs17883901	A	ATF7	0,01159616	activating transcription factor 7	activator
rs17883901	A	NANOG	0,01198007	Nanog homeobox	dual
rs17883901	A	CEBPA	0,01212469	CCAAT enhancer binding protein alpha	activator
rs17883901	A	ALX4	0,01213862	ALX homeobox 4	activator
rs17883901	A	NFIL3	0,01217346	nuclear factor, interleukin 3 regulated	repressor
rs17883901	A	HNF4A	0,0125721	hepatocyte nuclear factor 4 alpha	activator
rs17883901	A	NR4A2	0,01318777	nuclear receptor subfamily 4 group A member 2	activator
rs17883901	A	RXRA	0,01322997	retinoid X receptor alpha	activator
rs17883901	A	CREB1	0,0147806	cAMP responsive element binding protein 1	dual
rs17883901	A	CREB1	0,01562041	cAMP responsive element binding protein 1	dual
rs17883901	A	NR2E1	0,01567254	nuclear receptor subfamily 2 group E member 1	dual
rs17883901	A	HNF4A	0,01601905	hepatocyte nuclear factor 4 alpha	activator
rs17883901	A	GATA	0,01622611	glutaminyl-tRNA amidotransferase subunit QRSL1	unknown
rs17883901	A	JUN::FOS	0,0165114	Jun proto-oncogene, AP-1 transcription factor subunit	activator
rs17883901	A	CREB1	0,01658897	cAMP responsive element binding protein 1	dual
rs17883901	A	NR1H2::RXRA	0,01807333	nuclear receptor subfamily 1 group H member 2:retinoid X receptor alpha	activator
rs17883901	A	TRIM28	0,01824363	tripartite motif containing 28	activator
rs17883901	A	JDP2	0,0190496	Jun dimerization protein 2	repressor
rs17883901	A	RORA	0,0191615	RAR related orphan receptor A	activator
rs17883901	A	HSF	0,02007655	interleukin 6	activator
rs17883901	A	USF	0,02165686	Upstream stimulatory factors	activator

rs17883901	A	PAX6	0,02172257	paired box 6	activator
rs17883901	A	TBX21	0,02315743	T-box transcription factor 21	repressor
rs17883901	A	PAX5	0,02409916	paired box 5	activator
rs17883901	A	JDP2	0,02453651	Jun dimerization protein 2	repressor
rs17883901	A	NFE2L1::MAFG	0,02499941	nuclear factor, erythroid 2 like 1:MAF bZIP transcription factor G	activator
rs17883901	A	FOXJ3	0,02539159	forkhead box J3	activator
rs17883901	A	MYC	0,02599091	MYC proto-oncogene, bHLH transcription factor	dual
rs17883901	A	ARX	0,02700054	aristaless related homeobox	unknown
rs17883901	A	CEBPB	0,02702804	CCAAT enhancer binding protein beta	dual
rs17883901	A	BATF	0,02756623	basic leucine zipper ATF-like transcription factor	activator
rs17883901	A	HMGN3	0,02826825	high mobility group nucleosomal binding domain 3	unknown
rs17883901	A	ATF1	0,02899337	activating transcription factor 1 / glial cell derived neurotrophic factor	activator
rs17883901	A	NR4A2	0,03043427	nuclear receptor subfamily 4 group A member 2	activator
rs17883901	A	PAX6	0,03096494	paired box 6	activator
rs17883901	A	PRDM1	0,03174036	PR/SET domain 1	repressor
rs17883901	A	CEBP	0,03206944	CCAAT enhancer binding protein alpha	activator
rs17883901	A	Pax6	0,03213223	paired box 6	activator
rs17883901	A	EP300	0,03236763	E1A binding protein p300	activator
rs17883901	A	SOX17	0,03313213	SRY-box transcription factor 17	activator
rs17883901	A	JUNB	0,03423789	JunB proto-oncogene, AP-1 transcription factor subunit	activator
rs17883901	A	JDP2	0,03428448	Jun dimerization protein 2	repressor
rs17883901	A	NR1H4	0,03589925	nuclear receptor subfamily 1 group H member 4	repressor
rs17883901	A	ZNF282	0,03594888	zinc finger protein 282	repressor
rs17883901	A	SREBF1	0,03654425	sterol regulatory element binding transcription factor 1	activator
rs17883901	A	TEF	0,03717118	TEF transcription factor, PAR bZIP family member	activator
rs17883901	A	TCF7L2	0,04198696	transcription factor 7 like 2	dual
rs17883901	A	FOSL1	0,04299858	FOS like 1, AP-1 transcription factor subunit	unknown
rs17883901	A	BATF::JUN	0,04409598	basic leucine zipper ATF-like transcription factor	activator
rs17883901	A	NR2F1	0,04463238	nuclear receptor subfamily 2 group F member 1	dual
rs17883901	A	NFE2L1::MafG	0,04604332	nuclear factor, erythroid 2 like 1	activator

rs17883901	A	SREBF2	0,04733187	sterol regulatory element binding transcription factor 2	activator
rs17883901	A	ATF1	0,04748018	activating transcription factor 1 / glial cell derived neurotrophic factor	activator
rs17883901	A	ESR2	0,04834271	estrogen receptor 2	activator
rs17883901	A	JDP2	0,04934466	Jun dimerization protein 2	repressor
rs17883901	A	FOS	0,04988163	Fos proto-oncogene, AP-1 transcription factor subunit	unknown
C>T rs606548 of *GCLC*					
rs606548	C	WT1	0,00000038	WT1 transcription factor	unknown
rs606548	C	YY1	0,00000115	YY1 transcription factor	dual
rs606548	C	ATF3	0,00002455	activating transcription factor 3	dual
rs606548	C	MYC	0,00003182	MYC proto-oncogene, bHLH transcription factor	dual
rs606548	C	TCF3	0,00015253	transcription factor 3 / transcription factor 7 like 1	dual
rs606548	C	TCF3	0,00083532	transcription factor 3 / transcription factor 7 like 1	dual
rs606548	C	TCF4	0,00108915	transcription factor 4 / transcription factor 7 like 2	activator
rs606548	C	MESP1	0,00168894	mesoderm posterior bHLH transcription factor 1	activator
rs606548	C	BHLHE41	0,00196477	basic helix-loop-helix family member e41	repressor
rs606548	C	BHLHE40	0,00237336	basic helix-loop-helix family member e40	dual
rs606548	C	TFCP2	0,00262335	transcription factor CP2	activator
rs606548	C	USF2	0,00323042	upstream transcription factor 2, c-fos interacting	activator
rs606548	C	REST	0,00323336	RE1 silencing transcription factor	dual
rs606548	C	ASCL2	0,00337564	achaete-scute family bHLH transcription factor 2	unknown
rs606548	C	BHLHE40	0,00348578	basic helix-loop-helix family member e40	dual
rs606548	C	MYCN	0,00368425	MYCN proto-oncogene, bHLH transcription factor	activator
rs606548	C	Mycn	0,00392639	MYCN proto-oncogene, bHLH transcription factor	activator
rs606548	C	TCF3	0,00399713	transcription factor 3 / transcription factor 7 like 1	dual
rs606548	C	MNT	0,00405737	MAX network transcriptional repressor	dual
rs606548	C	MYC	0,00421842	MYC proto-oncogene, bHLH transcription factor	dual
rs606548	C	TFAP2	0,00472683	transcription factor AP-2 alpha	activator
rs606548	C	AP1	0,00479182	adaptor related protein complex 1	activator
rs606548	C	EGR3	0,00484729	early growth response 3	unknown
rs606548	C	MYOD1	0,00485676	myogenic differentiation 1	activator

rs606548	C	USF	0,00503902	Upstream stimulatory factors	activator
rs606548	C	REST	0,00558022	RE1 silencing transcription factor	dual
rs606548	C	EGR1	0,00558286	early growth response 1	activator
rs606548	C	ARNTL	0,00571248	aryl hydrocarbon receptor nuclear translocator like	repressor
rs606548	C	SIN3A	0,00576975	SIN3 transcription regulator family member A	dual
rs606548	C	Myog	0,00579294	myogenin	activator
rs606548	C	Tcf12	0,00594296	transcription factor 12	activator
rs606548	C	EBOX	0,00598713	enhancer box	activator
rs606548	C	MXI1	0,00609672	MAX interactor 1, dimerization protein	repressor
rs606548	C	TFCP2	0,00633846	transcription factor CP2	activator
rs606548	C	BHLHE41	0,00660087	basic helix-loop-helix family member e41	repressor
rs606548	C	MYC	0,00665261	MYC proto-oncogene, bHLH transcription factor	dual
rs606548	C	ZEB1	0,00666924	zinc finger E-box binding homeobox 1	dual
rs606548	C	MYC::MAX	0,00667017	MYC proto-oncogene, bHLH transcription factor _ MYC associated factor X	dual
rs606548	C	Myod1	0,00669435	myogenic differentiation 1	activator
rs606548	C	EGR4	0,0067055	early growth response 4	unknown
rs606548	C	MYC	0,00711481	MYC proto-oncogene, bHLH transcription factor	dual
rs606548	C	ARNT	0,00724873	aryl hydrocarbon receptor nuclear translocator	activator
rs606548	C	MLXIPL	0,00759103	MLX interacting protein like	repressor
rs606548	C	MAX	0,00823346	MYC associated factor X	dual
rs606548	C	E2F1	0,00840379	E2F transcription factor 1	repressor
rs606548	C	TBX5	0,00863614	T-box transcription factor 5	activator
rs606548	C	TAL1	0,00882408	TAL bHLH transcription factor 1, erythroid differentiation factor	activator
rs606548	C	HES5	0,00907627	hes family bHLH transcription factor 5	repressor
rs606548	C	Arnt	0,00908941	aryl hydrocarbon receptor nuclear translocator	activator
rs606548	C	ARNT	0,00928532	aryl hydrocarbon receptor nuclear translocator	activator
rs606548	C	EGR1	0,00935062	early growth response 1	activator
rs606548	C	NR2C2	0,00960724	nuclear receptor subfamily 2 group C member 2	activator
rs606548	C	NRF1	0,00969985	nuclear respiratory factor 1_nuclear factor, erythroid 2 like 1	activator
rs606548	C	MYC	0,0104547	MYC proto-oncogene, bHLH transcription factor	dual

rs606548	C	MYF6	0,01058168	myogenic factor 6	activator
rs606548	C	MLX	0,01145177	MAX dimerization protein MLX	repressor
rs606548	C	MAX	0,01181282	MYC associated factor X	dual
rs606548	C	EGR1	0,01192492	early growth response 1	activator
rs606548	C	HINFP	0,01199455	histone H4 transcription factor	activator
rs606548	C	E2F1	0,01199655	E2F transcription factor 1	repressor
rs606548	C	BHLHE40	0,01216586	basic helix-loop-helix family member e40	dual
rs606548	C	TFE3	0,01254485	transcription factor binding to IGHM enhancer 3	activator
rs606548	C	RREB1	0,01293411	ras responsive element binding protein 1	activator
rs606548	C	ARNT	0,01298143	aryl hydrocarbon receptor nuclear translocator	activator
rs606548	C	TCF3	0,01331265	transcription factor 3 / transcription factor 7 like 1	dual
rs606548	C	E2F	0,01335948	E2F transcription factors	activator
rs606548	C	POU2F2	0,01392187	POU class 2 homeobox 2	activator
rs606548	C	TAL1	0,01446152	TAL bHLH transcription factor 1, erythroid differentiation factor	activator
rs606548	C	MYF	0,01471224	Myogenic regulatory family	activator
rs606548	C	Myc	0,01515093	MYC proto-oncogene, bHLH transcription factor	dual
rs606548	C	MAX	0,01614799	MYC associated factor X	dual
rs606548	C	YY1	0,01697466	YY1 transcription factor	dual
rs606548	C	EGR2	0,01728248	early growth response 2	activator
rs606548	C	NRF1	0,01770854	nuclear respiratory factor 1_nuclear factor, erythroid 2 like 1	activator
rs606548	C	TBX4	0,01780036	T-box transcription factor 4	activator
rs606548	C	MEIS3	0,01785668	Meis homeobox 3 pseudogene 1	activator
rs606548	C	NHLH1	0,01833971	nescient helix-loop-helix 1	activator
rs606548	C	NHLH1	0,01840164	nescient helix-loop-helix 1	activator
rs606548	C	TBX5	0,01858407	T-box transcription factor 5	activator
rs606548	C	GLIS3	0,0189369	GLIS family zinc finger 3	dual
rs606548	C	TFE3	0,01926256	transcription factor binding to IGHM enhancer 3	activator
rs606548	C	GATA	0,01936247	glutaminyl-tRNA amidotransferase subunit QRSL1	unknown
rs606548	C	E2F1	0,01953315	E2F transcription factor 1	repressor
rs606548	C	MAX	0,02008856	MYC associated factor X	dual

rs606548	C	USF1	0,02023199	upstream transcription factor 1	activator
rs606548	C	SIN3A	0,02094519	SIN3 transcription regulator family member A	dual
rs606548	C	TGIF2	0,02108174	TGFB induced factor homeobox 2	repressor
rs606548	C	MYOD1	0,02135359	myogenic differentiation 1	activator
rs606548	C	NR1H4	0,0213629	nuclear receptor subfamily 1 group H member 4	repressor
rs606548	C	RREB1	0,02168069	ras responsive element binding protein 1	activator
rs606548	C	SETDB1	0,02187887	SET domain bifurcated histone lysine methyltransferase 1	repressor
rs606548	C	USF	0,02286497	Upstream stimulatory factors	activator
rs606548	C	PTEN	0,02290576	phosphatase and tensin homolog	unknown
rs606548	C	MSC	0,02298758	musculin	unknown
rs606548	C	TCF3	0,02317515	transcription factor 3 / transcription factor 7 like 1	dual
rs606548	C	TCF12	0,02334591	transcription factor 12	activator
rs606548	C	SMC3	0,02334965	structural maintenance of chromosomes 3	activator
rs606548	C	EGR3	0,02373409	early growth response 3	unknown
rs606548	C	MYC::MAX	0,0237419	MYC proto-oncogene, bHLH transcription factor _ MYC associated factor X	dual
rs606548	C	TFAP4	0,02406278	transcription factor AP-4	activator
rs606548	C	BHLHE40	0,02447855	basic helix-loop-helix family member e40	dual
rs606548	C	TFEB	0,02522784	transcription factor EB	activator
rs606548	C	MYC::MAX	0,0254963	MYC proto-oncogene, bHLH transcription factor _ MYC associated factor X	dual
rs606548	C	SPI1	0,02558332	Spi-1 proto-oncogene	activator
rs606548	C	RAD21	0,02579872	RAD21 cohesin complex component	activator
rs606548	C	TFAP2A	0,02593552	transcription factor AP-2 alpha	dual
rs606548	C	GLIS2	0,02638018	GLIS family zinc finger 2	dual
rs606548	C	E2F1	0,02786001	E2F transcription factor 1	repressor
rs606548	C	Atoh1	0,02806959	atonal bHLH transcription factor 1	dual
rs606548	C	ZBTB18	0,02815547	zinc finger and BTB domain containing 18	activator
rs606548	C	E2F1	0,02819874	E2F transcription factor 1	repressor
rs606548	C	CLOCK::ARNTL	0,02830329	clock circadian regulator:aryl hydrocarbon receptor nuclear translocator like	dual
rs606548	C	TFAP2C	0,02855949	transcription factor AP-2 gamma	activator
rs606548	C	EGR1	0,02857161	early growth response 1	activator

rs606548	C	NFE2	0,02869241	nuclear factor, erythroid 2	activator
rs606548	C	PKNOX2	0,02874821	PBX/knotted 1 homeobox 2	dual
rs606548	C	SIN3A	0,02988588	SIN3 transcription regulator family member A	dual
rs606548	C	TAL1	0,0299515	TAL bHLH transcription factor 1, erythroid differentiation factor	activator
rs606548	C	TGIF1	0,0312851	TGFB induced factor homeobox 1	repressor
rs606548	C	TFAP4	0,0328394	transcription factor AP-4	activator
rs606548	C	E2F1	0,03671325	E2F transcription factor 1	repressor
rs606548	C	MYC::MAX	0,03705931	MYC proto-oncogene, bHLH transcription factor _ MYC associated factor X	dual
rs606548	C	ESRRA	0,03771439	estrogen related receptor alpha	activator
rs606548	C	CTCF	0,03783071	CCCTC-binding factor	repressor
rs606548	C	TBX15	0,03793956	T-box transcription factor 15	repressor
rs606548	C	EGR1	0,04012118	early growth response 1	activator
rs606548	C	VDR	0,04065613	vitamin D receptor	activator
rs606548	C	TFAP2	0,0412301	transcription factor AP-2 alpha	activator
rs606548	C	MYF6	0,04190285	myogenic factor 6	activator
rs606548	C	ESRRA	0,04217532	estrogen related receptor alpha	activator
rs606548	C	TFAP4	0,04327519	transcription factor AP-4	activator
rs606548	C	SREBF1	0,04345022	sterol regulatory element binding transcription factor 1	activator
rs606548	C	ZBTB7B	0,04459099	zinc finger and BTB domain containing 7B	repressor
rs606548	C	ZBTB18	0,04524501	zinc finger and BTB domain containing 18	activator
rs606548	C	MAX	0,04711606	MYC associated factor X	dual
rs606548	C	MYOD1	0,04723757	myogenic differentiation 1	activator
rs606548	C	TCF12	0,04744339	transcription factor 12	activator
rs606548	C	MEIS3	0,04828668	Meis homeobox 3 pseudogene 1	activator
rs606548	C	TBX2	0,04971994	T-box transcription factor 2	repressor
rs606548	T	CTCF	0,00655383	CCCTC-binding factor	repressor
rs606548	T	RAD21	0,00759339	RAD21 cohesin complex component	activator
rs606548	T	ZBTB33	0,02007987	zinc finger and BTB domain containing 33	activator
rs606548	T	ZBTB7A	0,02410717	zinc finger and BTB domain containing 7A	repressor
rs606548	T	FOXC1	0,02563964	forkhead box C1	activator

rs606548	T	PAX1	0,0357732	paired box 1	activator
rs606548	T	PPARG	0,04137177	peroxisome proliferator activated receptor gamma	activator
rs606548	T	NR2C2	0,04343217	nuclear receptor subfamily 2 group C member 2	activator
rs606548	T	STAT5A	0,04515471	signal transducer and activator of transcription 5A	activator
rs606548	T	HMBOX1	0,04902403	homeobox containing 1	unknown
G>A rs636933 of *GCLC*					
rs636933	G	ESRRA	0	estrogen related receptor alpha	activator
rs636933	G	REST	0,00000001	RE1 silencing transcription factor	dual
rs636933	G	EP300	0,00012083	E1A binding protein p300	activator
rs636933	G	RAD21	0,00065368	RAD21 cohesin complex component	activator
rs636933	G	EGR1	0,00224434	early growth response 1	activator
rs636933	G	VDR	0,00315262	vitamin D receptor	activator
rs636933	G	SIN3A	0,00844079	SIN3 transcription regulator family member A	dual
rs636933	G	GLI2	0,00866745	GLI family zinc finger 2	dual
rs636933	G	RARG	0,00954046	retinoic acid receptor gamma	activator
rs636933	G	SMAD	0,01291437	SMAD family	activator
rs636933	G	RARA	0,01488799	retinoic acid receptor alpha	dual
rs636933	G	NFKB	0,01612059	nuclear factor kappa-light-chain-enhancer of activated B cells	activator
rs636933	G	SOX15	0,01677425	SRY-box transcription factor 15	repressor
rs636933	G	PROX1	0,01885267	prospero homeobox 1	repressor
rs636933	G	ZIC2	0,02170427	Zic family member 2	activator
rs636933	G	RARG	0,02283718	retinoic acid receptor gamma	activator
rs636933	G	GLIS1	0,02399974	GLIS family zinc finger 1	dual
rs636933	G	NR2C2	0,0250681	nuclear receptor subfamily 2 group C member 2	activator
rs636933	G	RARG	0,0251907	retinoic acid receptor gamma	activator
rs636933	G	ZNF219	0,02747611	zinc finger protein 219	dual
rs636933	G	VDR	0,02832608	vitamin D receptor	activator
rs636933	G	SREBF1	0,03057097	sterol regulatory element binding transcription factor 1	activator
rs636933	G	CHD2	0,03200899	chromodomain helicase DNA binding protein 2	activator
rs636933	G	ZBTB7B	0,03256491	zinc finger and BTB domain containing 7B	repressor

rs636933	G	RXRG	0,03287829	retinoid X receptor gamma	unknown
rs636933	G	TBX5	0,03376663	T-box transcription factor 5	activator
rs636933	G	TBX21	0,03387379	T-box transcription factor 21	repressor
rs636933	G	SOX14	0,03528567	SRY-box transcription factor 14	repressor
rs636933	G	GLIS2	0,0358471	GLIS family zinc finger 2	dual
rs636933	G	HINFP	0,03695242	histone H4 transcription factor	activator
rs636933	G	PPARA	0,03761804	peroxisome proliferator activated receptor alpha	dual
rs636933	G	HINFP	0,04393317	histone H4 transcription factor	activator
rs636933	G	TBR1	0,04599496	T-box brain transcription factor 1	dual
rs636933	G	E2F3	0,04896531	E2F transcription factor 3	activator
rs636933	A	PBX	0,0071141	PBX homeobox	activator
rs636933	A	RHOXF1	0,00795352	Rhox homeobox family member 1	repressor
rs636933	A	CTCFL	0,00804316	CCCTC-binding factor like	activator
rs636933	A	RHOXF1	0,00926763	Rhox homeobox family member 1	repressor
rs636933	A	NFKB	0,00953596	nuclear factor kappa-light-chain-enhancer of activated B cells	activator
rs636933	A	ESR2	0,01347985	estrogen receptor 2	activator
rs636933	A	NR3C1	0,01437735	nuclear receptor subfamily 3 group C member 1	activator
rs636933	A	HDAC2	0,01734651	histone deacetylase 2	dual
rs636933	A	IKZF2	0,01965367	IKAROS family zinc finger 2	dual
rs636933	A	SETDB1	0,02460507	SET domain bifurcated histone lysine methyltransferase 1	repressor
rs636933	A	SIX6	0,030484	SIX homeobox 6	unknown
rs636933	A	RFX1	0,03185975	regulatory factor X1	activator
rs636933	A	SIX1	0,03257415	SIX homeobox 1	repressor
rs636933	A	ESR1	0,03292515	estrogen receptor 1	activator
rs636933	A	SPI1	0,03640234	Spi-1 proto-oncogene	activator
rs636933	A	ELK1	0,03768681	ETS transcription factor ELK1	activator
rs636933	A	ESR1	0,03890678	estrogen receptor 1	activator
rs636933	A	IKZF2	0,0432231	IKAROS family zinc finger 2	dual
rs636933	A	IKZF1	0,04506305	IKAROS family zinc finger 1	repressor
rs636933	A	REST	0,04732747	RE1 silencing transcription factor	dual

rs636933	A	TEAD1	0,04818332	TEA domain transcription factor 1	activator
T>G rs648595 *GCLC*					
rs648595	T	AP1	0,00061688	adaptor related protein complex 1	activator
rs648595	T	NANOG	0,00439521	Nanog homeobox	dual
rs648595	T	CHD2	0,01014112	chromodomain helicase DNA binding protein 2	activator
rs648595	T	OTX	0,01326595	orthodenticle homeobox	activator
rs648595	T	TFAP4	0,01521282	transcription factor AP-4	activator
rs648595	T	SOX9	0,01623398	SRY-box transcription factor 9	activator
rs648595	T	SOX10	0,01828762	SRY-box transcription factor 10	activator
rs648595	T	Sox6	0,01985427	SRY-box transcription factor 6	dual
rs648595	T	SOX4	0,0225504	SRY-box transcription factor 4	activator
rs648595	T	TCF12	0,02415139	transcription factor 12	activator
rs648595	T	SOX17	0,02745142	SRY-box transcription factor 17	activator
rs648595	T	PAX2	0,02851283	paired box 2	activator
rs648595	T	RHOXF1	0,02950364	Rhox homeobox family member 1	repressor
rs648595	T	NFKB	0,02962136	nuclear factor kappa-light-chain-enhancer of activated B cells	activator
rs648595	T	SOX11	0,03307767	SRY-box transcription factor 11	activator
rs648595	T	SRY	0,03319703	sex determining region Y	activator
rs648595	T	Sox2	0,03409888	SRY-box transcription factor 2	dual
rs648595	T	SOX2	0,03636354	SRY-box transcription factor 2	dual
rs648595	T	HOXD13	0,04241829	homeobox D13	unknown
rs648595	T	E2F2	0,0430016	E2F transcription factor 2	activator
rs648595	T	RFX5	0,04302185	regulatory factor X5	activator
rs648595	T	SRF	0,04317877	serum response factor	activator
rs648595	T	SOX9	0,04340642	SRY-box transcription factor 9	activator
rs648595	T	SREBF	0,04659847	SREBF chaperone	activator
rs648595	T	SOX9	0,04858002	SRY-box transcription factor 9	activator
rs648595	G	HES1	0,00177254	hes family bHLH transcription factor 1	activator
rs648595	G	TFCP2	0,00298368	transcription factor CP2	activator
rs648595	G	Klf1	0,0039168	Kruppel like factor 1	activator

rs648595	G	ZSCAN4	0,00468854	zinc finger and SCAN domain containing 4	unknown
rs648595	G	KLF4	0,00762738	Kruppel like factor 4	activator
rs648595	G	Klf4	0,00837755	Kruppel like factor 4	activator
rs648595	G	SP3	0,00877507	Sp3 transcription factor	dual
rs648595	G	TP53	0,01500937	tumor protein p53	activator
rs648595	G	NRF1	0,01511558	nuclear respiratory factor 1_nuclear factor, erythroid 2 like 1	activator
rs648595	G	KLF5	0,01706954	Kruppel like factor 5	activator
rs648595	G	MYC::MAX	0,01736381	MYC proto-oncogene, bHLH transcription factor _ MYC associated factor X	dual
rs648595	G	HIF1A::ARNT	0,01827785	aryl hydrocarbon receptor nuclear translocator	activator
rs648595	G	NRF1	0,01847777	nuclear respiratory factor 1_nuclear factor, erythroid 2 like 1	activator
rs648595	G	SP4	0,01896841	Sp4 transcription factor	dual
rs648595	G	NANOG	0,0200426	Nanog homeobox	dual
rs648595	G	EGR1	0,02101839	early growth response 1	activator
rs648595	G	TFE3	0,02163426	transcription factor binding to IGHM enhancer 3	activator
rs648595	G	GLIS2	0,02235389	GLIS family zinc finger 2	dual
rs648595	G	GATA	0,02246203	glutaminyl-tRNA amidotransferase subunit QRSL1	unknown
rs648595	G	AHR::ARNT	0,02323213	aryl hydrocarbon receptor nuclear translocator	activator
rs648595	G	HES7	0,02436479	hes family bHLH transcription factor 7	repressor
rs648595	G	ZNF143	0,02500005	zinc finger protein 143	activator
rs648595	G	EGR2	0,02500072	early growth response 2	activator
rs648595	G	TFEB	0,02552882	transcription factor EB	activator
rs648595	G	USF	0,02556531	Upstream stimulatory factors	activator
rs648595	G	TP53	0,02566996	tumor protein p53	activator
rs648595	G	BHLHE41	0,02762474	basic helix-loop-helix family member e41	repressor
rs648595	G	TFEC	0,02823141	transcription factor EC	activator
rs648595	G	SP1	0,02830371	Sp1 transcription factor	repressor
rs648595	G	Bhlhe40	0,02907825	basic helix-loop-helix family member e40	dual
rs648595	G	MYC::MAX	0,03031536	MYC proto-oncogene, bHLH transcription factor _ MYC associated factor X	dual
rs648595	G	MAX	0,03050051	MYC associated factor X	dual
rs648595	G	WT1	0,03103435	WT1 transcription factor	unknown

rs648595	G	BHLHE40	0,03197154	basic helix-loop-helix family member e40	dual
rs648595	G	MYC	0,03240011	MYC proto-oncogene, bHLH transcription factor	dual
rs648595	G	TP53	0,03347032	tumor protein p53	activator
rs648595	G	FOXO6	0,03520702	forkhead box O6	activator
rs648595	G	FOXJ3	0,0352405	forkhead box J3	activator
rs648595	G	NKX2-1	0,03691174	NK2 homeobox 1	activator
rs648595	G	PBX1	0,03835999	PBX homeobox 1	activator
rs648595	G	KLF7	0,03836844	Kruppel like factor 7	activator
rs648595	G	RBPJ	0,04418103	recombination signal binding protein for immunoglobulin kappa J region	activator
A>C rs761142 of *GCLC*					
rs761142	A	YY1	0,00234753	YY1 transcription factor	dual
rs761142	A	HOXB6	0,00275678	homeobox B6	unknown
rs761142	A	Ddit3::Cebpa	0,00851731	_CCAAT enhancer binding protein alpha	activator
rs761142	A	CEBPA	0,00881561	CCAAT enhancer binding protein alpha	activator
rs761142	A	STAT	0,00918312	sterol O-acyltransferase 1	unknown
rs761142	A	MYB	0,0098942	MYB proto-oncogene, transcription factor	activator
rs761142	A	RFX2	0,01074207	regulatory factor X2	activator
rs761142	A	YY1	0,01097337	YY1 transcription factor	dual
rs761142	A	AHR	0,01163674	aryl hydrocarbon receptor	dual
rs761142	A	NR3C1	0,01245523	nuclear receptor subfamily 3 group C member 1	activator
rs761142	A	E2F1	0,01432682	E2F transcription factor 1	repressor
rs761142	A	EN1	0,01620891	engrailed homeobox 1	activator
rs761142	A	ATF4	0,01658051	activating transcription factor 4	activator
rs761142	A	CUX1	0,01952144	cut like homeobox 1	activator
rs761142	A	CHD2	0,02006561	chromodomain helicase DNA binding protein 2	activator
rs761142	A	NFY	0,02107803	nuclear transcription factor Y subunit	dual
rs761142	A	HLF	0,02173271	HLF transcription factor, PAR bZIP family member	activator
rs761142	A	BCL6B	0,0221881	BCL6B transcription repressor	dual
rs761142	A	GBX2	0,02491298	gastrulation brain homeobox 2	unknown
rs761142	A	YY2	0,02548136	YY2 transcription factor	dual

rs761142	A	CEBPB	0,02658066	CCAAT enhancer binding protein beta	dual
rs761142	A	MEOX1	0,02813979	mesenchyme homeobox 1	dual
rs761142	A	TEAD1	0,02814403	TEA domain transcription factor 1	activator
rs761142	A	BARX1	0,02843163	BARX homeobox 1	unknown
rs761142	A	BCL6B	0,03189653	BCL6B transcription repressor	dual
rs761142	A	ZBTB33	0,03205876	zinc finger and BTB domain containing 33	activator
rs761142	A	E2F4	0,03247437	E2F transcription factor 4	activator
rs761142	A	MXI1	0,03331496	MAX interactor 1, dimerization protein	repressor
rs761142	A	EP300	0,03332363	E1A binding protein p300	activator
rs761142	A	RFX5	0,03354332	regulatory factor X5	activator
rs761142	A	HOXD3	0,03506551	homeobox D3	unknown
rs761142	A	CEBP	0,03516007	CCAAT enhancer binding protein alpha	activator
rs761142	A	NKX6-1	0,04395918	NK6 homeobox 1	dual
rs761142	A	NOBOX	0,04438225	NOBOX oogenesis homeobox	activator
rs761142	A	SP1	0,04583886	Sp1 transcription factor	repressor
rs761142	A	HOXA6	0,04814392	homeobox A6	unknown
rs761142	A	YY1	0,04914022	YY1 transcription factor	dual
rs761142	A	MEOX1	0,0491462	mesenchyme homeobox 1	dual
rs761142	A	LHX8	0,04960732	LIM homeobox 8	unknown
rs761142	A	MEOX2	0,0498538	mesenchyme homeobox 2	activator
rs761142	C	HNF4A	0,00002893	hepatocyte nuclear factor 4 alpha	activator
rs761142	C	NRF1	0,00378918	nuclear respiratory factor 1_nuclear factor, erythroid 2 like 1	activator
rs761142	C	SOX14	0,00608772	SRY-box transcription factor 14	repressor
rs761142	C	REST	0,00635676	RE1 silencing transcription factor	dual
rs761142	C	AR	0,00780215	androgen receptor	dual
rs761142	C	TP53	0,01129834	tumor protein p53	activator
rs761142	C	HNF4	0,01226678	hepatocyte nuclear factor 4 alpha	activator
rs761142	C	NRF1	0,01515224	nuclear respiratory factor 1_nuclear factor, erythroid 2 like 1	activator
rs761142	C	PRDM1	0,02163379	PR/SET domain 1	repressor
rs761142	C	NFY	0,02216072	nuclear transcription factor Y subunit	dual

rs761142	C	EBF1	0,02326326	EBF transcription factor 1	activator
rs761142	C	VDR	0,02414417	vitamin D receptor	activator
rs761142	C	PBX3	0,0246509	PBX homeobox 3	activator
rs761142	C	MYC	0,02533566	MYC proto-oncogene, bHLH transcription factor	dual
rs761142	C	EGR1	0,03049554	early growth response 1	activator
rs761142	C	HES7	0,03073847	hes family bHLH transcription factor 7	repressor
rs761142	C	NR1H	0,03645429	nuclear receptor subfamily 1 group H	activator
rs761142	C	TP53	0,04041564	tumor protein p53	activator
rs761142	C	TFCP2	0,04080412	transcription factor CP2	activator
rs761142	C	MYB	0,045021	MYB proto-oncogene, transcription factor	activator
rs761142	C	ATF3	0,04750237	activating transcription factor 3	dual
rs761142	C	RFX5	0,04869586	regulatory factor X5	activator
rs761142	C	PTEN	0,04935093	phosphatase and tensin homolog	unknown
rs761142	C	E2F1	0,0494137	E2F transcription factor 1	repressor
C>T rs2301022 of *GCLM*					
rs2301022	T	PDX1	0,004476	Pancreatic and duodenal homeobox 1	activator
rs2301022	T	ZSCAN26	0,0089533	Zinc finger and SCAN domain containing 26	activator
rs2301022	T	ELF3	0,0090783	E74 like ETS transcription factor 3	activator
rs2301022	T	ZNF652	0,00947956	zinc finger protein 652	repressor
rs2301022	T	PAX4	0,01151472	paired box 4	repressor
rs2301022	T	SMARCC1	0,01393287	SWI/SNF related, matrix associated, actin dependent regulator of chromatin subfamily c member 1	activator
rs2301022	T	JDP2	0,01933904	Jun dimerization protein 2	repressor
rs2301022	T	FOXP3	0,01974083	forkhead box P3	dual
rs2301022	T	ZBTB33	0,02059947	zinc finger and BTB domain containing 33	activator
rs2301022	T	PRRX2	0,02139004	paired related homeobox 2	unknown
rs2301022	T	MEF2A	0,02506893	myocyte enhancer factor 2A	activator
rs2301022	T	OTX2	0,02549287	orthodenticle homeobox 2	activator
rs2301022	T	DMBX1	0,02724183	diencephalon/mesencephalon homeobox 1	activator
rs2301022	T	RHOXF2	0,0277196	Rhox homeobox family member 2	unknown
rs2301022	T	JUND	0,02883945	JunB proto-oncogene, AP-1 transcription factor subunit	activator

rs2301022	T	PITX1	0,03254459	paired like homeodomain 1	activator
rs2301022	T	JUNB	0,03254707	JunB proto-oncogene, AP-1 transcription factor subunit	activator
rs2301022	T	CUX1	0,03256081	cut like homeobox 1	activator
rs2301022	T	HNF4A	0,03335128	hepatocyte nuclear factor 4 alpha	activator
rs2301022	T	UNCX	0,03362804	UNC homeobox	unknown
rs2301022	T	PAX5	0,03459243	paired box 5	activator
rs2301022	T	FOSL2	0,03746132	FOS like 2, AP-1 transcription factor subunit	unknown
rs2301022	T	HOXB5	0,0396166	homeobox B5	unknown
rs2301022	T	BARX1	0,03988725	BARX homeobox 1	unknown
rs2301022	T	TFAP2A	0,04041147	transcription factor AP-2 alpha	dual
rs2301022	T	SRF	0,04192369	serum response factor	activator
rs2301022	T	VSX2	0,04753928	visual system homeobox 2	dual
rs2301022	T	FOS	0,04858529	Fos proto-oncogene, AP-1 transcription factor subunit	unknown
rs2301022	T	POU6F2	0,04872158	POU class 6 homeobox 2	activator
rs2301022	C	SPIC	0,00106353	Spi-C transcription factor	activator
rs2301022	C	TP53	0,00378427	tumor protein p53	activator
rs2301022	C	STAT2::STAT1	0,00687506	signal transducer and activator of transcription 2::1	activator
rs2301022	C	RUNX2	0,00954222	RUNX family transcription factor 2	activator
rs2301022	C	SPI1	0,01051148	Spi-1 proto-oncogene	activator
rs2301022	C	IRF3	0,01250049	interferon regulatory factor 3	activator
rs2301022	C	SPI1	0,01390572	Spi-1 proto-oncogene	activator
rs2301022	C	PAX3	0,01695678	paired box 3	activator
rs2301022	C	GABPA	0,0175597	GA binding protein transcription factor subunit alpha	activator
rs2301022	C	IRF1	0,01998595	interferon regulatory factor 1	activator
rs2301022	C	HDAC2	0,02096926	histone deacetylase 2	dual
rs2301022	C	IRF5	0,02496072	interferon regulatory factor 5	activator
rs2301022	C	MYB	0,02652524	MYB proto-oncogene, transcription factor	activator
rs2301022	C	IRF1	0,0277301	interferon regulatory factor 1	activator
rs2301022	C	HDAC2	0,02775999	histone deacetylase 2	dual
rs2301022	C	IRF8	0,03824357	interferon regulatory factor 8	dual

rs2301022	C	HNF4A	0,03829104	hepatocyte nuclear factor 4 alpha	activator
rs2301022	C	TFCP2	0,03985543	transcription factor CP2	activator
rs2301022	C	SETDB1	0,04329857	SET domain bifurcated histone lysine methyltransferase 1	repressor
rs2301022	C	IRF5	0,04754444	interferon regulatory factor 5	activator
T>C rs3827715 of *GCLM*					
rs3827715	T	AP1	0,00335889	adaptor related protein complex 1	activator
rs3827715	T	NEUROD2	0,00927587	neuronal differentiation 2	activator
rs3827715	T	TAL1	0,00961019	TAL bHLH transcription factor 1, erythroid differentiation factor	activator
rs3827715	T	ZBTB18	0,01034002	zinc finger and BTB domain containing 18	activator
rs3827715	T	RUNX1	0,01313097	RUNX family transcription factor 1	activator
rs3827715	T	FOXJ3	0,01791793	forkhead box J3	activator
rs3827715	T	FOXM1	0,02014479	forkhead box M1	repressor
rs3827715	T	SRY	0,02922733	sex determining region Y	activator
rs3827715	T	FOXH1	0,03090103	forkhead box H1	activator
rs3827715	T	ZBTB18	0,03263217	zinc finger and BTB domain containing 18	activator
rs3827715	T	SOX8	0,03353594	SRY-box transcription factor 8	unknown
rs3827715	T	FOXO3	0,03447913	forkhead box O3	activator
rs3827715	T	NR3C1	0,03476121	nuclear receptor subfamily 3 group C member 1	activator
rs3827715	T	RUNX1	0,03556729	RUNX family transcription factor 1	activator
rs3827715	T	RXRG	0,03721051	retinoid X receptor gamma	unknown
rs3827715	T	TCF21	0,04100362	transcription factor 21	dual
rs3827715	T	IRF7	0,0422366	interferon regulatory factor 7	activator
rs3827715	T	FOXO1	0,04366622	forkhead box O1	activator
rs3827715	T	ATOH1	0,0489216	atonal bHLH transcription factor 1	dual
rs3827715	C	XBP1	0,00081675	X-box binding protein 1 pseudogene 1	activator
rs3827715	C	MLX	0,00093065	MAX dimerization protein MLX	repressor
rs3827715	C	HIF1A::ARNT	0,00124462	aryl hydrocarbon receptor nuclear translocator	activator
rs3827715	C	HIF1A	0,00137194	hypoxia inducible factor 1 subunit alpha	activator
rs3827715	C	AHR::ARNT::HIF1A	0,00148157	aryl hydrocarbon receptor nuclear translocator	activator
rs3827715	C	GRHL1	0,00149854	grainyhead like transcription factor 1	activator

rs3827715	C	HEY1	0,00150794	hes related family bHLH transcription factor with YRPW motif 1	
rs3827715	C	XBP1	0,00201296	X-box binding protein 1 pseudogene 1	activator
rs3827715	C	MYC	0,00234251	MYC proto-oncogene, bHLH transcription factor	dual
rs3827715	C	XBP1	0,00276718	X-box binding protein 1 pseudogene 1	activator
rs3827715	C	GRHL1	0,00370821	grainyhead like transcription factor 1	activator
rs3827715	C	MYC::MAX	0,00401597	MYC proto-oncogene, bHLH transcription factor _ MYC associated factor X	dual
rs3827715	C	MYC	0,00464712	MYC proto-oncogene, bHLH transcription factor	dual
rs3827715	C	CREB3L1	0,00548545	cAMP responsive element binding protein 3 like 1	activator
rs3827715	C	BHLHE40	0,00550655	basic helix-loop-helix family member e40	dual
rs3827715	C	CREB3L2	0,00561675	cAMP responsive element binding protein 3 like 2	activator
rs3827715	C	BHLHE40	0,00784495	basic helix-loop-helix family member e40	dual
rs3827715	C	MYC::MAX	0,00801343	MYC proto-oncogene, bHLH transcription factor _ MYC associated factor X	dual
rs3827715	C	SREBF2	0,00826871	sterol regulatory element binding transcription factor 2	activator
rs3827715	C	TP53	0,0092016	tumor protein p53	activator
rs3827715	C	HES7	0,00931655	hes family bHLH transcription factor 7	repressor
rs3827715	C	CLOCK::ARNTL	0,01058606	clock circadian regulator:aryl hydrocarbon receptor nuclear translocator like	dual
rs3827715	C	GMEB2	0,01105621	glucocorticoid modulatory element binding protein 2	activator
rs3827715	C	PROX1	0,01110257	prospero homeobox 1	repressor
rs3827715	C	CREB3L1	0,0116786	cAMP responsive element binding protein 3 like 1	activator
rs3827715	C	TFCP2	0,01276672	transcription factor CP2	activator
rs3827715	C	ARNT	0,01283822	aryl hydrocarbon receptor nuclear translocator	activator
rs3827715	C	USF2	0,01468488	upstream transcription factor 2, c-fos interacting	activator
rs3827715	C	MYC::MAX	0,01471497	MYC proto-oncogene, bHLH transcription factor _ MYC associated factor X	dual
rs3827715	C	HIF1A	0,0155005	hypoxia inducible factor 1 subunit alpha	activator
rs3827715	C	FOXN1	0,01588139	forkhead box N1	activator
rs3827715	C	MYC	0,01634499	MYC proto-oncogene, bHLH transcription factor	dual
rs3827715	C	ZEB1	0,01696481	zinc finger E-box binding homeobox 1	dual
rs3827715	C	CREB3	0,01931951	cAMP responsive element binding protein 3 like 4	activator
rs3827715	C	BRCA1	0,02090425	BRCA1 DNA repair associated	activator
rs3827715	C	ZBTB14	0,02388803	zinc finger and BTB domain containing 14	activator

rs3827715	C	BHLHE41	0,02417896	basic helix-loop-helix family member e41	repressor
rs3827715	C	BRCA1	0,02501203	BRCA1 DNA repair associated	activator
rs3827715	C	XBP1	0,02642666	X-box binding protein 1 pseudogene 1	activator
rs3827715	C	NANOG	0,0310723	Nanog homeobox	dual
rs3827715	C	TCF3	0,03189894	transcription factor 3 / transcription factor 7 like 1	dual
rs3827715	C	TP53	0,03215823	tumor protein p53	activator
rs3827715	C	MYB	0,03539749	MYB proto-oncogene, transcription factor	activator
rs3827715	C	VDR	0,03559699	vitamin D receptor	activator
rs3827715	C	BHLHE40	0,04000795	basic helix-loop-helix family member e40	dual
rs3827715	C	GMEB2	0,04261066	glucocorticoid modulatory element binding protein 2	activator
C>A rs7517826 of *GCLM*					
rs7517826	C	NFE2L1	0,00000024	nuclear factor, erythroid 2 like 1	activator
rs7517826	C	SOX17	0,00023461	SRY-box transcription factor 17	activator
rs7517826	C	IRF	0,00068809	tripartite motif containing 63	unknown
rs7517826	C	TGIF1	0,00210978	TGFB induced factor homeobox 1	repressor
rs7517826	C	CREB1	0,00319809	cAMP responsive element binding protein 1	dual
rs7517826	C	MEIS1	0,00391969	Meis homeobox 1	activator
rs7517826	C	IRF	0,00435958	tripartite motif containing 63	unknown
rs7517826	C	STAT4	0,00438168	signal transducer and activator of transcription 4	activator
rs7517826	C	NFE2L1::MAFG	0,00486267	nuclear factor, erythroid 2 like 1	activator
rs7517826	C	TEAD1	0,00487045	TEA domain transcription factor 1	activator
rs7517826	C	ESRRA	0,00508248	estrogen related receptor alpha	activator
rs7517826	C	MEIS2	0,00566992	Meis homeobox 2	activator
rs7517826	C	MEIS3	0,00613075	Meis homeobox 3 pseudogene 1	activator
rs7517826	C	Sox17	0,00616632	SRY-box transcription factor 17	activator
rs7517826	C	NFE2L1::MafG	0,00764634	nuclear factor, erythroid 2 like 1	activator
rs7517826	C	PAX2	0,00796219	paired box 2	activator
rs7517826	C	Pax2	0,00814851	paired box 2	activator
rs7517826	C	IRF1	0,00889233	interferon regulatory factor 1	activator
rs7517826	C	CREB1	0,00898061	cAMP responsive element binding protein 1	dual

rs7517826	C	HESX1	0,01077647	HESX homeobox 1	unknown
rs7517826	C	MEIS3	0,0112138	Meis homeobox 3 pseudogene 1	activator
rs7517826	C	AP1	0,01288358	adaptor related protein complex 1	activator
rs7517826	C	EP300	0,01335874	E1A binding protein p300	activator
rs7517826	C	VSX1	0,0147064	visual system homeobox 1	unknown
rs7517826	C	RUNX1	0,01511712	RUNX family transcription factor 1	activator
rs7517826	C	MEIS3	0,015509	Meis homeobox 3 pseudogene 1	activator
rs7517826	C	POU2F1	0,015944	POU class 2 homeobox 1	activator
rs7517826	C	NR4A2	0,01656418	nuclear receptor subfamily 4 group A member 2	activator
rs7517826	C	TGIF2	0,01804187	TGFB induced factor homeobox 2	repressor
rs7517826	C	ARHGEF12	0,0184403	Rho guanine nucleotide exchange factor 12	activator
rs7517826	C	Mecom	0,01870954	MDS1 and EVI1 complex locus	unknown
rs7517826	C	CREB1	0,01906193	cAMP responsive element binding protein 1	dual
rs7517826	C	PKNOX1	0,02099474	PBX/knotted 1 homeobox 1	unknown
rs7517826	C	LHX9	0,02257196	LIM homeobox 9	unknown
rs7517826	C	MEIS2	0,02277129	Meis homeobox 2	activator
rs7517826	C	MEIS1	0,02299105	Meis homeobox 1	activator
rs7517826	C	MEIS1	0,02539711	Meis homeobox 1	activator
rs7517826	C	RUNX1	0,02706844	RUNX family transcription factor 1	activator
rs7517826	C	PKNOX2	0,02816623	PBX/knotted 1 homeobox 2	dual
rs7517826	C	MEIS1	0,02908212	Meis homeobox 1	activator
rs7517826	C	TGIF1	0,0300918	TGFB induced factor homeobox 1	repressor
rs7517826	C	PAX5	0,03057097	paired box 5	activator
rs7517826	C	AP1	0,03205362	adaptor related protein complex 1	activator
rs7517826	C	Meis1	0,03400455	Meis homeobox 1	activator
rs7517826	C	SRY	0,03409278	sex determining region Y	activator
rs7517826	C	YY1	0,03444788	YY1 transcription factor	dual
rs7517826	C	SIX4	0,03490717	SIX homeobox 4	dual
rs7517826	C	CREB3	0,03516947	cAMP responsive element binding protein 3 like 4	activator
rs7517826	C	HNF4	0,03588184	hepatocyte nuclear factor 4 alpha	activator

rs7517826	C	SPI1	0,03781513	Spi-1 proto-oncogene	activator
rs7517826	C	STAT	0,03810443	sterol O-acyltransferase 1	unknown
rs7517826	C	TP53	0,04000995	tumor protein p53	activator
rs7517826	C	ESRRA	0,04481598	estrogen related receptor alpha	activator
rs7517826	C	Sox3	0,04669724	SRY-box transcription factor 3	activator
rs7517826	C	ZNF713	0,04730671	zinc finger protein 713	unknown
rs7517826	A	CCDC6	0,00000001	coiled-coil domain containing 6	unknown
rs7517826	A	POU2F1	0,00228069	POU class 2 homeobox 1	activator
rs7517826	A	HMGA1	0,0033464	high mobility group AT-hook 1	unknown
rs7517826	A	SOX14	0,00406735	SRY-box transcription factor 14	repressor
rs7517826	A	SOX21	0,00657245	SRY-box transcription factor 21	repressor
rs7517826	A	SRY	0,01077225	sex determining region Y	activator
rs7517826	A	EN1	0,01158819	engrailed homeobox 1	repressor
rs7517826	A	TLX2	0,01277172	T cell leukemia homeobox 2	activator
rs7517826	A	HMGA1	0,01862471	high mobility group AT-hook 1	unknown
rs7517826	A	DLX1	0,01995175	distal-less homeobox 1	dual
rs7517826	A	PDX1	0,02008051	Pancreatic and duodenal homeobox 1	activator
rs7517826	A	DLX4	0,020445	distal-less homeobox 4	unknown
rs7517826	A	CENPB	0,02056078	centromere protein B	repressor
rs7517826	A	FOXJ1	0,02101787	forkhead box J1	activator
rs7517826	A	PRRX2	0,02139004	paired related homeobox 2	unknown
rs7517826	A	HINFP	0,02140895	histone H4 transcription factor	activator
rs7517826	A	DLX3	0,02151809	distal-less homeobox 3	unknown
rs7517826	A	DLX2	0,02151908	distal-less homeobox 2	unknown
rs7517826	A	DLX2	0,02222268	distal-less homeobox 2	unknown
rs7517826	A	POU3F3	0,02392953	POU class 3 homeobox 3	activator
rs7517826	A	DLX5	0,02412036	distal-less homeobox 5	activator
rs7517826	A	RELA	0,02493694	RELA proto-oncogene, NF-kB subunit	dual
rs7517826	A	DLX6	0,0255299	distal-less homeobox 6	unknown
rs7517826	A	MSX2	0,02565044	msh homeobox 2	repressor

rs7517826	A	Prrx2	0,02727507	paired related homeobox 2	unknown
rs7517826	A	RELA	0,02898625	RELA proto-oncogene, NF-kB subunit	dual
rs7517826	A	Pdx1	0,02975664	Pancreatic and duodenal homeobox 1	activator
rs7517826	A	DDIT3::CEBPA	0,02985602	_CCAAT enhancer binding protein alpha	activator
rs7517826	A	MSX1	0,03064996	msh homeobox 1	repressor
rs7517826	A	POU3F1	0,03152657	POU class 3 homeobox 1	unknown
rs7517826	A	NFKB	0,03230733	nuclear factor kappa-light-chain-enhancer of activated B cells	activator
rs7517826	A	PROP1	0,03234605	PROP paired-like homeobox 1	activator
rs7517826	A	SOX3	0,03529995	SRY-box transcription factor 3	activator
rs7517826	A	ARID3A	0,03595632	AT-rich interaction domain 3A	activator
rs7517826	A	BBX	0,03922261	BBX high mobility group box domain containing	unknown
rs7517826	A	ALX4	0,03980972	ALX homeobox 4	activator
rs7517826	A	IRF7	0,04032572	interferon regulatory factor 7	activator
rs7517826	A	BSX	0,04186821	brain specific homeobox	activator
rs7517826	A	EP300	0,04276296	E1A binding protein p300	activator
rs7517826	A	PHOX2B	0,04298898	paired like homeobox 2B	activator
rs7517826	A	PDX1	0,04366881	Pancreatic and duodenal homeobox 1	activator
rs7517826	A	PHOX2B	0,04445184	paired like homeobox 2B	activator
rs7517826	A	STAT	0,04843201	sterol O-acyltransferase 1	unknown
rs7517826	A	FOXD3	0,04900784	forkhead box D3	repressor
