# Supplementary material for: The Impact of Genetic Polymorphisms in Glutamate-Cysteine Ligase, a Key Enzyme of Glutathione Biosynthesis, on Ischemic Stroke Risk and Brain Infarct Size
Source: Life (Basel). 2022 Apr 18;12(4):602. doi: 10.3390/life12040602 (PMC9032935; doi:10.3390/life12040602)
Supplement: Supplementary file 1 [file life-12-00602-s001.zip › Supplementary_table_S1.pdf]

Supplementary table S1

Statistics for all *mbmdr*-models of G×G and G×E interactions associated with the risk of ischemic stroke*

Risk factors/SNPs	Number of *n*-order models, n (%)							
	2n (75)	%	3n (492)	%	4n (1999)	%	5n (8541)	%
Smoking	19	12,7	154	10,4	709	8,9	2949	6,9
Alcohol	15	10,0	69	4,7	270	3,4	1657	3,9
Fruit/Vegetable	7	4,7	73	4,9	293	3,7	1747	4,1
rs12524494	4	2,7	56	3,8	294	3,7	2195	5,1
rs17883901	5	3,3	69	4,7	387	4,8	2152	5,0
rs606548	5	3,3	66	4,5	372	4,7	2203	5,2
rs636933	3	2,0	51	3,5	328	4,1	1849	4,3
rs648595	7	4,7	71	4,8	412	5,2	2188	5,1
rs761142	4	2,7	55	3,7	345	4,3	1912	4,5
rs2301022	19	12,7	147	10,0	723	9,0	2853	6,7
rs3827715	5	3,3	65	4,4	368	4,6	1983	4,6
rs7517826	5	3,3	56	3,8	351	4,4	1920	4,5
rs11556924	6	4,0	68	4,6	416	5,2	2123	5,0
rs12449964	5	3,3	46	3,1	317	4,0	1900	4,4
rs12646447	4	2,7	56	3,8	378	4,7	2212	5,2
rs2417957	4	2,7	55	3,7	265	3,3	1865	4,4
rs4322086	19	12,7	155	10,5	761	9,5	3159	7,4
rs6511720	6	4,0	61	4,1	375	4,7	2219	5,2
rs783396	4	2,7	53	3,6	326	4,1	1839	4,3
rs899997	4	2,7	50	3,4	306	3,8	1787	4,2
Summary statistics:								
Risk factors	41	27,3	296	20,1	1272	15,9	6353	14,9
GCLM/*GCLC* SNPs	57	38,0	636	43,1	3580	44,8	19255	45,1
GWAS SNPs	52	34,7	544	36,9	3144	39,3	17104	40,0

*G×G (SNP×SNP) and G×E (SNP×risk factor) interactions were analyzed by the model-based multifactor dimensionality reduction (mbmdr) method (Calle et al, 2010).
