# Supplementary material for: The Impact of Genetic Polymorphisms in Glutamate-Cysteine Ligase, a Key Enzyme of Glutathione Biosynthesis, on Ischemic Stroke Risk and Brain Infarct Size
Source: Life (Basel). 2022 Apr 18;12(4):602. doi: 10.3390/life12040602 (PMC9032935; doi:10.3390/life12040602)
Supplement: Supplementary file 1 [file life-12-00602-s001.zip › Supplementary_table_S2.pdf]

Statistics for the best *mbmdr*-models of G×G and G×E interactions associated with the risk of ischemic stroke*

Risk factors/SNPs	Number of *n*-order models, n (%)							
	2n (19)	%	3n (123)	%	4n (499)	%	5n (2135)	%
Smoking	7	18,4	62	16,8	233	11,7	752	7,0
Alcohol	1	2,6	17	4,6	56	2,8	275	2,6
Fruit/Vegetable	1	2,6	16	4,3	58	2,9	296	2,8
rs12524494	0	0,0	7	1,9	47	2,4	462	4,3
rs17883901	0	0,0	9	2,4	74	3,7	429	4,0
rs606548	1	2,6	12	3,3	87	4,4	475	4,4
rs636933	1	2,6	7	1,9	52	2,6	323	3,0
rs648595	1	2,6	14	3,8	98	4,9	461	4,3
rs761142	1	2,6	9	2,4	77	3,9	322	3,0
rs2301022	8	21,1	47	12,7	222	11,1	844	7,9
rs3827715	1	2,6	10	2,7	83	4,2	384	3,6
rs7517826	0	0,0	11	3,0	95	4,8	349	3,3
rs11556924	3	7,9	21	5,7	102	5,1	464	4,3
rs12449964	1	2,6	13	3,5	80	4,0	397	3,7
rs12646447	1	2,6	12	3,3	82	4,1	513	4,8
rs2417957	1	2,6	5	1,4	37	1,9	397	3,7
rs4322086	7	18,4	57	15,4	282	14,1	1034	9,7
rs6511720	2	5,3	19	5,1	89	4,5	1042	9,8
rs783396	1	2,6	16	4,3	74	3,7	794	7,4
rs899997	0	0,0	5	1,4	68	3,4	662	6,2
Summary statistics:								
Risk factors	9	23,7	95	25,7	347	17,4	1323	12,4
GCLM/*GCLC* SNPs	13	34,2	126	34,1	835	41,8	4049	37,9
GWAS SNPs	16	42,1	148	40,1	814	40,8	5303	49,7

*G×G (SNP×SNP) and G×E (SNP×risk factor) interactions were analyzed by the model-based multifactor dimensionality reduction (mbmdr) method (Calle et al, 2010).
The best mbmdr-models include the 25% of models with the lowest permutation *P*-values.
