# Supplementary material for: The Impact of Genetic Polymorphisms in Glutamate-Cysteine Ligase, a Key Enzyme of Glutathione Biosynthesis, on Ischemic Stroke Risk and Brain Infarct Size
Source: Life (Basel). 2022 Apr 18;12(4):602. doi: 10.3390/life12040602 (PMC9032935; doi:10.3390/life12040602)
Supplement: Supplementary file 1 [file life-12-00602-s001.zip › Supplementary_table_S3.pdf]

Supplementary table S3

Statistics for all *mbmdr*-models of G×G and G×E interactions associated with brain infarct size*

Risk factors/SNPs	Number of *n*-order models, n (%)							
	2n (25)	%	3n (109)	%	4n (524)	%	5n (2143)	%
Smoking	4	8,0	34	10,4	128	6,1	565	5,3
Alcohol	1	2,0	13	4,0	66	3,1	380	3,5
Fruit/Vegetable	0	0,0	12	3,7	66	3,1	369	3,4
rs12524494	1	2,0	8	2,4	54	2,6	315	2,9
rs17883901	3	6,0	19	5,8	159	7,6	828	7,7
rs606548	2	4,0	16	4,9	100	4,8	562	5,2
rs636933	2	4,0	14	4,3	123	5,9	612	5,7
rs648595	2	4,0	22	6,7	118	5,6	565	5,3
rs761142	3	6,0	8	2,4	80	3,8	433	4,0
rs2301022	3	6,0	19	5,8	144	6,9	727	6,8
rs3827715	2	4,0	17	5,2	103	4,9	581	5,4
rs7517826	0	0,0	7	2,1	72	3,4	444	4,1
rs11556924	4	8,0	22	6,7	178	8,5	898	8,4
rs12449964	1	2,0	10	3,1	105	5,0	536	5,0
rs12646447	5	10,0	37	11,3	207	9,9	815	7,6
rs2417957	6	12,0	33	10,1	182	8,7	830	7,7
rs4322086	9	18,0	17	5,2	59	2,8	265	2,5
rs6511720	0	0,0	2	0,6	42	2,0	306	2,9
rs783396	1	2,0	8	2,4	44	2,1	353	3,3
rs899997	1	2,0	9	2,8	66	3,1	331	3,1
Summary statistics:								
Risk factors	5	10,0	59	18,0	260	12,4	1314	12,3
GCLM/*GCLC* SNPs	18	36,0	130	39,8	953	45,5	5067	47,3
GWAS SNPs	27	54,0	138	42,2	883	42,1	4334	40,4

*G×G (SNP×SNP) and G×E (SNP×risk factor) interactions were analyzed by the model-based multifactor dimensionality reduction (mbmdr) method (Calle et al, 2010).
