# Supplementary material for: The Impact of Genetic Polymorphisms in Glutamate-Cysteine Ligase, a Key Enzyme of Glutathione Biosynthesis, on Ischemic Stroke Risk and Brain Infarct Size
Source: Life (Basel). 2022 Apr 18;12(4):602. doi: 10.3390/life12040602 (PMC9032935; doi:10.3390/life12040602)
Supplement: Supplementary file 1 [file life-12-00602-s001.zip › Supplementary_table_S4.pdf]

Statistics for the best *mbmdr*-models of G×G and G×E interactions associated with brain infarct size*

Risk factors/SNPs	Number of *n*-order models, n (%)							
	2n (7)	%	3n (27)	%	4n (131)	%	5n (536)	%
Smoking	2	14,3	7	8,6	26	5,0	565	5,3
Alcohol	0	0,0	3	3,7	13	2,5	380	3,5
Fruit/Vegetable	0	0,0	2	2,5	16	3,1	369	3,4
rs12524494	0	0,0	0	0,0	12	2,3	315	2,9
rs17883901	0	0,0	4	4,9	33	6,3	828	7,7
rs606548	1	7,1	1	1,2	26	5,0	562	5,2
rs636933	0	0,0	5	6,2	30	5,7	612	5,7
rs648595	1	7,1	9	11,1	36	6,9	565	5,3
rs761142	0	0,0	2	2,5	18	3,4	433	4,0
rs2301022	1	7,1	4	4,9	35	6,7	727	6,8
rs3827715	0	0,0	4	4,9	33	6,3	581	5,4
rs7517826	0	0,0	1	1,2	16	3,1	444	4,1
rs11556924	1	7,1	5	6,2	52	9,9	898	8,4
rs12449964	0	0,0	4	4,9	27	5,2	536	5,0
rs12646447	2	14,3	17	21,0	63	12,0	815	7,6
rs2417957	2	14,3	9	11,1	46	8,8	830	7,7
rs4322086	4	28,6	3	3,7	19	3,6	265	2,5
rs6511720	0	0,0	1	1,2	8	1,5	306	2,9
rs783396	0	0,0	0	0,0	5	1,0	353	3,3
rs899997	0	0,0	0	0,0	10	1,9	331	3,1
Summary statistics:								
Risk factors	2	14,3	12	14,8	55	10,5	1314	12,3
GCLM/*GCLC* SNPs	3	21,4	30	37,0	239	45,6	5067	47,3
GWAS SNPs	9	64,3	39	48,1	230	43,9	4334	40,4

*G×G (SNP×SNP) and G×E (SNP×risk factor) interactions were analyzed by the model-based multifactor dimensionality reduction (mbmdr) method (Calle et al, 2010).
The best mbmdr-models include the 25% of models with the lowest permutation *P*-values.
